# Dialogues Across Time? Conceptualising the Temporal Relationships of Palimpsests in the Upper Palaeolithic Cave Art of El Castillo (Cantabria, Spain)

**DOI:** 10.1007/s10816-025-09717-5

**Published:** 2025-06-12

**Authors:** Izzy Wisher, Eduardo Palacio-Pérez

**Affiliations:** 1 Department of Archaeology and Heritage Studies, Aarhus University, Aarhus, Denmark; 2 Department of Linguistics, Cognitive Science and Semiotics, Aarhus University, Aarhus, Denmark; 3 Center of Prehistoric Caves, Government of Cantabria, Cantabria, Spain

**Keywords:** Upper Palaeolithic art, Palimpsests, Harris Matrix, Relational Approaches, Cave art

## Abstract

Cave sites were frequently reused throughout the Upper Palaeolithic, with many sites within south-western Europe having deep chronologies of activity. The repeated engagement with the same caves, or spaces within caves, is evident in superimpositions of cave art depictions within these sites. Whilst these palimpsests in Upper Palaeolithic cave art have been extensively studied with regard to understanding the relative chronology of art within a particular region or site, they have not been understood from an ontological perspective. Upper Palaeolithic artist’s engagement with motifs produced by their predecessors, regardless of cultural continuity, may indicate dialogical interactions occurring across time between culturally and temporally distinct groups of hunter-gatherers. In this paper, we propose a conceptual framework—inspired by relationality and contemporary rock art production—for understanding these temporal interactions. Focusing on the case study of El Castillo, we argue that these engagements across time may tentatively indicate aspects of long-term continuity in the ontology of Upper Palaeolithic hunter-gatherers, reflected in cave art palimpsests.

**Supplementary Information:**

The online version contains supplementary material available at 10.1007/s10816-025-09717-5.

## Introduction

Caves are attractive spaces for symbolic activities. The dynamism of cave environments and the sensorial experience evoked in caves has been conceptually and empirically demonstrated to be amongst the main attractors for human cultural behaviours occurring within these spaces (Fazenda et al., 2017; Hodgson, 2008; Pastoors & Weninger, 2011; Pettitt, 2016; Prijatelj & Skeates, 2018; Till, 2014; Wisher et al., 2024). The use of caves for symbolic purposes appears to be a cross-cultural phenomenon with roots deep in our evolutionary past (Moyes, 2012). In recent years, it has been demonstrated that caves were utilised by distant hominin species such as *H. naledi* (Randolph-Quinney, 2015; Pettitt, 2022; cf: Martinón-Torres et al., 2024), possible artistic motifs produced by Neanderthals (Hoffmann et al., 2018; Marquet et al., 2023; cf: White et al., 2020) and unusual structures were produced in the encompassing darkness of caves by Neanderthals (Jaubert et al., 2016), and diverse examples of burials (Pettitt, 2002), ritual practices (Jaubert et al., 2016), and parietal and portable art were produced in caves by *H. sapiens* in a breadth of different geographical areas (Clottes, 2001; 2012). It is perhaps no surprise, therefore, that palimpsests are a common feature of cave sites. The repeated reuse and engagement with the same caves, or indeed the same spaces within caves, reflects these deep temporal relationships with places. This is particularly evident in cave art, with many cave sites in northern Spain reflecting palimpsests of artistic behaviours, produced by different hunter-gatherer groups painting and engraving on the same walls as their predecessors (Garate et al., 2021; Ibero et al., 2024).

The palimpsest nature of Palaeolithic cave art has been the focus of academic attention since the inception of Palaeolithic art as a discipline. During the early twentieth century, an important question of Palaeolithic art research was understanding its chronology. Cave art palimpsests, particularly in the caves of Altamira, La Pasiega, and El Castillo, were studied with the aim of establishing a relative chronology of Palaeolithic art from the superimpositions of depictions (*i.e*. Cartailhac & Breuil, 1906; Alcalde et al., 1911; Breuil et al., 1913; Breuil, 1952). In the mid-late twentieth century, Laming-Emperaire (1962) and Leroi-Gourhan (1965, 1982) continued these approaches, but incorporated the idea that, in some cases, there could be synchronous associations of motifs. Since the end of the twentieth century, the introduction of AMS radiocarbon and U-Th dating methods has made it possible to establish a long tradition of decoration at sites such as Cougnac and Cosquer in France (Lorblanchet, 1994; Valladas et al., 2017), and Peña Candamo, Tito Bustillo, Llonín, Altamira, El Castillo, La Pasiega, and La Garma in Spain (Balbín & Alcolea, 2013; Corchón et al., 2017; Fortea et al., 2004; González Sáinz, 2003; 2007; Pike et al., 2012; Valladas et al., 2001). Only recently has this been addressed in terms of repeated frequentation and symbolic reappropriation by different human groups of the decorated spaces of the caves (Garate et al., 2021; Lorblanchet, 2010; Ochoa, 2017; Ortega et al., 2020).

Whilst cave art palimpsests have been extensively studied in the context of understanding chronologies of artistic behaviour within the European Upper Palaeolithic, the particular behaviours of interaction captured within the superimpositions of different motifs have not been explored in detail. Cave art palimpsests attest to temporal interactions occurring between distinct Upper Palaeolithic cultures, reflected in one artist placing a motif within the same cave or even the same rock surface as their distant predecessor. There has been an increasing appreciation in recent years for how different dimensions of cave art, such as tactility, technique, gestures, psychological responses, acoustics, and lighting technologies, may have structured or constrained the production of motifs within caves (e.g., Fazenda et al., 2017; Fritz & Tosello, 2007; Hodgson, 2008; Medina-Alcaide et al., 2021; Pettitt, 2016; Till, 2014; Wisher & Needham, 2023; Wisher et al., 2023, 2024). However, the dimension of temporal interactions has not yet been considered with regard to how the presence of older motifs within a cave could have been perceived, engaged with, or ontologically understood by subsequent Upper Palaeolithic visitors or artists.

In this paper, we intend to advance conceptual understandings of how temporal relationships may be observable in the palimpsests of decorated panels, particularly those which have long chronologies of interaction. We intend to answer two key questions: (i) is it possible to access the socio-cultural meaning of these interactions? and (ii) could dialogical interactions across time indicate continuities in aspects of Upper Palaeolithic ontologies in south-western Europe, despite changing techno-cultural practices? We develop a theoretical framework for understanding these interactions, drawing from previous theoretical discussions of palimpsests in the archaeological record and anthropological insights of rock art production in contemporary societies that have long traditions of rock art production. We present different categories for understanding temporal relationships in Upper Palaeolithic cave art and suggest the ontological implications these relationships may have had for Upper Palaeolithic artists. Our conceptual discussion is grounded within a case study, focusing on two panels from El Castillo cave (Cantabria, Spain) that evidence the superimpositions of depictions with significant time depth. We selected El Castillo for four key reasons: (i) it is located in a region in which the reuse of caves is a recurring behaviour throughout the Upper Palaeolithic, with a very marked interaction between different phases of decoration (Garate et al., 2021); (ii) it has panels with clear examples of superimpositions that have been previously studied (González Sáinz & Ruiz-Redondo 2010); (iii) its parietal art has several direct U-Th and AMS radiocarbon dates that can facilitate the “anchoring” of different phases of the palimpsests to specific chronological periods (Pike et al., 2012; Valladas et al., 2001); and (iv) the long stratigraphy within its entrance, which yielded some portable art examples, supports the attribution of parietal depictions to Upper Palaeolithic chrono-cultural periods (Cabrera et al., 2005). We propose that within the art of El Castillo, different dialogical interactions between artists across time are present and tentatively may reflect some ontological continuities between hunter-gatherer groups who occupied and decorated the cave at different moments in time.

## Site Background

El Castillo cave is situated within Monte Castillo, a karstic conical-shaped mountain in the Pas River Basin and strategically located to observe migrating animal herds (Ortega-Martínez & Ruiz-Rednondo, 2008). Monte Castillo includes more than 40 caves; six contain Palaeolithic cave art and four (El Castillo, Las Chimeneas, La Pasiega and Las Monedas) have been included in the World Heritage list (Palacio-Pérez, 2024). El Castillo was discovered in 1903 by Hermilio Alcalde del Rio. The art was subsequently studied by Alcalde del Rio and Breuil (Alcalde del Río et al., 1911), whilst the excavation of the rock-shelter entrance was directed by Obermaier between 1910 and 1914. The excavations revealed an extensive archaeological sequence that yielded several portable art objects (Breuil & Obermaier, 1912a, 1912b, 1913, 1914). More recent excavations have furthered the understanding of this archaeological sequence, demonstrating the oldest layers date back to the early Middle Palaeolithic with repeated, and near continuous, occupation throughout the Upper Palaeolithic (Cabrera, 1984; Bernaldo de Quirós et al., 2015, Maillo et al., 2023).

Since the initial study of the parietal art, several researchers have unveiled new figures in different areas of El Castillo (Ripoll, 1956; González Echegaray, 1964; García Guinea & González Echegaray, 1966; González Echegaray & Moure, 1970; Ripoll, 1972; González García, 1999; 2001) and only recently has a complete monograph of the art been produced (Groenen & Groenen, *in press*). In total, there are 2964 depictions in El Castillo, with non-figurative depictions (*e.g*. hand-stencils, discs, abstract signs) dominating the assemblage (Groenen et al., 2013; Groenen & Groenen 2019; González Sainz et al., 2013). There has also been significant academic attention focused on understanding the chronology, composition, content, and distribution of the motifs. Through new digital imaging techniques, Groenen and Groenen (and colleagues) have comprehensively evaluated the depictions within El Castillo, demonstrating that there were multiple phases of artistic activity represented spatially throughout the cave (Warzée et al., 2007; Groenen, 2008, 2014; Groenen & Groenen, 2017; 2019), including in later prehistoric periods (Ontañón & Teira, 2016). For the Upper Palaeolithic art, U-Th and AMS radiocarbon dating supports that the phases of this art occurred across a large temporal breadth, with the oldest art phase represented by the production of red discs and hand-stencils (Pike et al., 2012; García-Diez et al., 2015), and the youngest phases featuring naturalistic animal depictions in charcoal (Moure et al., 1996; Valladas et al., 2001; Table 1).

**Table 1:** AMS radiocarbon and U-Th dates from the depictions of El Castillo. Radiocarbon dates have been recalibrated in OxCal 4.3 with 95.4% probability. U-Th dates derive from overlying calcite and represent minimum ages for the depictions. See cited references for further information

Depiction	Panel/gallery	Dating method	Date (cal. B.P.)	References
Red circle	Ceiling of the hands	U-Th	40,800	Pike et al., 2012
Handprint	Panel of the hands	U-Th	37,300	Pike et al., 2012
Stippled negative hand stencil	Ceiling of the hands	U-Th	24,340	Pike et al., 2012
Indeterminate animal	Corridor of the ceiling of the hands	U-Th	22,880	Pike et al., 2012
Large red stippled disc	Gallery of the discs	U-Th	41,400	Pike et al., 2012
Bison 18a	Polychrome panel	AMS radiocarbon	15,332–14,352	Valladas et al., 2001
		AMS radiocarbon	16,237–15,098	Valladas et al., 2001
		AMS radiocarbon	16,721–15,898	Valladas et al., 2001
Bison 18b	Polychrome panel	AMS radiocarbon	16,020–14,827	Valladas et al., 2001
Bison 18c	Polychrome panel	AMS radiocarbon	12,690–12,080	Valladas et al., 2001
		AMS radiocarbon	13,296–12,996	Valladas et al., 2001
		AMS radiocarbon	12,790–12,518	Valladas et al., 2001
Bison 19	Polychrome panel	AMS radiocarbon	16,695–15,920	Valladas et al., 2001
		AMS radiocarbon	16,810–15,981	Valladas et al., 2001
		AMS radiocarbon	17,016–16,163	Valladas et al., 2001
		AMS radiocarbon	17,557–16,635	Valladas et al., 2001
Horse 27/28	Unknown	AMS radiocarbon	20,949–20,030	Valladas et al., 2001
		AMS radiocarbon	23,614–22,510	Valladas et al., 2001
Ibex 56	Unknown	AMS radiocarbon	17,261–16,387	Valladas et al., 2001
		AMS radiocarbon	18,174–17,475	Valladas et al., 2001

## A Framework for Conceptualising Temporal Relationships

### Defining Palimpsests in Parietal Art

Palimpsests are an intrinsic feature of the archaeological record. Traditionally defined, a palimpsest refers to the process of erasing past traces on a material (*e.g*. parchment) and adding new traces onto the same material (Bailey, 2007; Colwell, 2022). In archaeology, the term is more generally used to refer to the superimposition of material traces as a result of past activities undertaken in the same area. There have been several approaches towards defining the characteristics of palimpsests, particularly those that consider the conceptual constraints of palimpsests in understanding past behaviour in the archaeological record. Bailey (2007), most notably, frames one of the challenges of approaching palimpsests as “time perspectivism”. This perspective emphasises that palimpsests, regardless of whether successive layers erase and/or are directly or partially superimposed on past activities, average out behavioural patterns; they do not truly reflect the pattern of individual actions. For Bailey (2007), the question of how to approach palimpsests is one of scale, from the micro-scale, where one may attempt to understand each individual action that formed a palimpsest, to the macro-scale, where the palimpsest is treated as one unit that represents a long temporal pattern of behaviour. In the context of site formation, identifying the former has been framed as a fallacy with strong advocacy for using palimpsests only to understand coarser scales of behaviour (Dibble et al., 2017).

However, palimpsests in parietal art are uniquely positioned to facilitate both the identification of the micro- and macro-scale of past behaviours. Unlike an archaeological site, where an object deposited in one action may be removed, modified, redeposited, or otherwise manipulated by subsequent actions, rock art is fixed in place after its production. Intimate moments of individual actions are preserved on the panel in the form of the material traces of pigment application, but the broader pattern of repeated behaviour over extended temporal periods can also be understood when perceiving the entire panel. Interactions between different scales of behaviour are thus preserved in rock art palimpsests, reflecting how a subsequent individual action may have been informed by the existing material traces of past actions and, in turn, how these successive individual actions reflect broader temporal patterns of engagements with rock art making. The potential of parietal art palimpsests to illuminate different scales of interaction has been previously recognised within the context of interactions in contemporary graffiti, where the presence of existing motifs may encourage the addition of the same motif, as a continuation of meaning, or the addition of motifs that engage in a dialogue with existing motifs, defined by Sapwell (2017) as singular and multiple palimpsests, respectively. In both cases, the palimpsest reflects interactions occurring at different moments in time, with past actions actively shaping and influencing artists.

Despite the significant potential of rock art palimpsests to inform about “the past in the past”—how previous behavioural traces were understood and engaged with by past societies—there has yet to be an approach that attempts to understand the temporal interactions that are embedded within rock art palimpsests. Approaches towards palimpsests in Palaeolithic cave art instead primarily focus on issues of chronology; superimpositions of motifs within a palimpsest are perceived passively, as merely a consequence of the extensive chronology of Upper Palaeolithic art. Some attempts have been made to more actively evaluate cave art palimpsests, predominantly for engraved panels, that have sought to determine whether the palimpsest was produced synchronously to intentionally create visual complexity or was the product of incidental superimpositions over time, where the artist did not perceive faded engraved figures as they produced a fresh engraving (Fritz & Tosello, 2007; Feruglio et al., 2019). However, it is important to note that despite the deep time dimension of Palaeolithic cave art, particularly within the same site, superimpositions are surprisingly rare. Where they do exist, they appear to be deliberate rather than incidental; superimpositions do not merely appear to be a result of a lack of suitable surfaces to depict on. This raises an important question about *why* Upper Palaeolithic artists decided to produce depictions in relation to existing depictions in a cave art site.

### Anthropological Insights

Insights from the production of rock art by contemporary societies with long traditions of rock art production can thus enrich the conceptual discussions in archaeology (Domingo et al., 2017; Lorblanchet, 2010), particularly concerning the nature of these palimpsests: how they were formed by, negotiated, or contributed to social, cultural, and ontological dimensions. Many contemporary societies can often attest to hundreds, thousands, or even tens of thousands of years of rock art production, supported by their oral traditions and archaeological evidence, within which temporally distant motifs are continuously engaged with through dialogical interactions that reinvigorate and renew the depictions.

In Aboriginal Australian rock art, particularly in the Kimberley and Arnhem Land regions, panels often have a deep chronology; oral traditions and direct archaeological evidence demonstrate this rock art dates back to at least 15,000 B.P. and are perhaps as old as c.40,000 B.P. (Bednarik, 2010, 2014; Langley & Taçon, 2010). These panels also exhibit repeated refreshing of depictions and the persistence of specific motifs across several thousand years (Langley & Taçon, 2010). A common theme in Aboriginal rock art is that depictions are imbued with ancestral power, with depictions understood to have been originally produced by *Wandjina* (ancestral spirit) that transformed themselves into images that remain sentient and powerful (Blundell et al., 2018). Engaging with temporally distant depictions thus facilitates the recreation and continuity of ancestral events; rock art depictions are reanimated, and the events they represent are brought into the present (Morphy, 1999). Depictions are active within this ontological conception and understood to be alive with their own energies that must be maintained (Morphy, 1991, 2003; O’Connor et al., 2008; Porr, 2018). Old depictions are therefore refreshed to maintain both their ancestral power and spiritual connections and to ensure people and land remain healthy (Blundell et al., 2018; Morphy, 1991, 2003; Porr, 2018). For Yanguwa rock art making in the Gulf of Carpentaria, for example, the erosion or fading of depictions is understood as the ancestral spirits “taking back” knowledge, indicating that the land or community is in poor health (Brady, 2016). Newer depictions were occasionally placed in relation to existing depictions as a way to incorporate new knowledge into the Dreaming, such as colonial contact, or to enrich storytelling by the active engagement with existing depictions (Brady et al., 2016; Frieman & May, 2020; O’Connor et al., 2008). Thus, the palimpsest of depictions that emerge through these actions reflects the accumulation of active cultural behaviours that engaged with pre-existing depictions in different contexts. Importantly, different types of relationships within the palimpsest (*e.g*. repainting vs. close spatial associations) reflect different kinds of engagements (*e.g*. maintaining ancestral connections vs. storytelling) that are intrinsically associated with cultural ontologies. Similar examples of engagements with pre-existing rock art panels are present elsewhere. In North America, for example, Indigenous groups often stress the importance of maintaining active relationships with the rock art panels through the maintenance of panels and/or the addition of new motifs to existing compositions that reinforce and harness the potency of the original image (Keyser & Whitley, 2006). Rock art in the Negev Desert (Israel) has a relatively complete chronology of making from 5000 B.P. to the present day, and newer engraved motifs, identified by their fresh colouring, are often superimposed on older motifs (Anati, 1999; Eisenberg-Degen et al., 2016). In doing this, present-day groups producing the motifs are directly linking their presence in an area to the past, simultaneously signing the landscape and communicating ownership (Eisenberg-Degen et al., 2016). The recent phase of rock art production also appears to include acts of refreshing and integrating newer motifs alongside existing motifs, consciously bringing the past into a present context and, in doing so, intentionally insinuating a historical continuum to the art (Eisenberg-Degen et al., 2016).

Tensions are also evident within rock art palimpsests. Schaafsma and Tsosie (2009; 2014) demonstrate that rock art can be perceived as having dangerous, potent power that provokes specific responses. They describe how during a flu epidemic, the Navajo deliberately defaced motifs present on their reservation that were believed to have been created by witches. Such engagements demonstrate that dialogical relationships in rock art palimpsests can intend to “silence” or erase the voices of the past, rather than being collaborative conversations that maintain or engage with ancestral motifs. The rock art site of Áísínai’pi (Writing-on-Stone) in southern Alberta, Canada, similarly reflects such dialogues. The site contains several Indigenous rock art motifs, attributed as messages from ancestral spirits that were historically revered by the Niitsítapi before European colonisation (Klassen, 2005). In the early-mid twentieth century, Euro-Canadian settlers inscribed their names and initials into the site, a deliberate act of control and erasure of Indigenous culture (Klassen, 2005). The deliberate superimposition of motifs by people culturally separated from the pre-existing motifs can thus reflect certain tensions of control or power within a palimpsest. It is also interesting to note that conceptual palimpsests of rock art may reflect the reconception of historical motifs to reclaim such acts of cultural erasure or loss. Within contemporary San art, historical rock art motifs that were temporally and culturally severed from their original context have been reused and reconceived in recent decades (Baracchini & Monney, 2018). San artists recognise the rock art as produced from their ancestors and create new cultural contexts for the motifs by incorporating them into their own art (Baracchini & Monney, 2018). In doing so, a conceptual “palimpsest of meaning” (Bailey, 2007) is created, where temporally separated depictions are attributed new symbolism.

Although these examples are diverse and derive from significantly distinct cultural contexts, they serve to emphasise that relationships between motifs in a rock art palimpsest are rarely unintentional. They often reflect deliberate engagements with the existing motifs that facilitate the integration of older motifs within new contexts, whether this is through maintaining the original depiction and reinforcing its historical meaning, creating new meanings through the addition of new motifs, or the reproduction of motifs that are temporally and culturally divorced from the present context. Whilst dialogical interactions with existing motifs have specific ontological and cultural significance, in the context of the aforementioned rock art examples, the broader theme of dialogues through palimpsests of motifs appears to be pervasive. As Sapwell (2017) emphasises, modern Western societies also frequently engage in dialogues with parietal motifs; literal examples of these kinds of dialogues are evident in what Sapwell refers to as “latrinalia” or “toilet graffiti”. Here, the presence of one type of motif (*e.g*. a lipstick mark, phone number, or short provocative phrase) will encourage repeated engagements that “respond” to the original motif. This serves to emphasise that dialogical interactions—although inherently having diverse cultural and ontological permutations—are ubiquitous across distinct forms of parietal art.

### Categorising Dialogical Interactions

The insights from anthropological accounts of engagements with art palimpsests in contemporary societies encourage a new conception of the relationships that may be represented in rock art palimpsests. The relationships between depictions in a palimpsest are rarely passive; they are intentional and active, infusing the palimpsest with new meaning. This resonates with relational conceptions of the art making process, that serve to emphasise that producing marks is a *lively* act, where relations unfold between different human and non-human agents rather than agency being solely within the human artist (Gell, 1998; Gormley, 2004). This perspective is succinctly captured by Gormley (2004): “art is not a noun; it’s a verb, a process”. In this sense, to appreciate the multitude of interactions and meanings locked within palimpsests, they must be understood through the unfolding relations that categorise their making. In recent years, there have been significant efforts more generally to de-centre the final form of rock art motifs to allow space for appreciating the fluid relations embedded within the making of depictions (Díaz-Guardamino, 2020; Merion Jones, 2017; Moro Abadía & Porr, 2022 and references therein; Wisher & Needham, 2023; Wisher et al., 2024). These have emphasised the importance of understanding Palaeolithic art and rock art more broadly as not just the passive final form, but rather as the dynamics between human and non-human agents that emerge from—and are embedded within—cultural and ontological milieus (Davidson, 2023; Moro Abadía & Porr, 2022). In appreciating these dynamics, parietal art can be understood in an agential way as directly influencing and being responsive to human engagements.

To appreciate the distributed agency embedded in art making and infused with the insights from anthropological examples of rock art production, the following three categories can be defined to describe the relationships which may exist between depictions in rock art palimpsests. All three categories may exist within the same palimpsest or indeed may characterise how one depiction relates to other motifs on the panel, and each category has theoretical implications for understanding the dialogical and ontological relationships of the depictions.

1. Intentional additive. Intentional additive relationships are defined here as the deliberate engagement with pre-existing depictions on a panel through the addition of a new depiction. This resonates with the anthropological examples of dialogues that occur when artists add motifs to existing rock art panels. Within this category are two distinct ways of intentionally engaging with existing depictions: (1.1) repainting and (1.2) modifying to integrate the depiction into a new context (*e.g*. through the addition of new motifs). In the former, existing depictions may be directly traced to draw the image into the present context and/or to ensure its longevity. In the latter, existing depictions may become part of a new ensemble of depictions, *e.g*. through the partial superimposition of a new depiction or the addition of features to a pre-existing motif. In both cases, the older depiction becomes integrated into a new context: presencing the past (Chapman, 1994).

2. Intentional obscuration. Intentional obscuration refers to the deliberate obscuring or obliteration of existing depictions in a rock art palimpsest, for example through superimposing a new depiction onto an existing one so that the original form can no longer be distinguished. This resonates with potent, reactionary engagements with rock art attested to within anthropological accounts, but also reflects a kind of dialogue that intends to enhance, control, or “silence” the agency of existing depictions. The obscuration of existing motifs can either be complete, where a new depiction almost entirely obscures an existing depiction through directly being superimposed over the top, or partial, where a new depiction mostly obscures an existing depiction. The former may be difficult to discern, but may be revealed through the use of different imaging techniques that can help reveal the presence of underlying depictions, *e.g*. multispectral imaging (Bayarri et al., 2021).

3. Incidental. It is also important to stress that the association between depictions in a palimpsest may also be incidental, particularly for palimpsests that have deep chronologies. Older depictions may merely be significantly faded and difficult to distinguish with the naked eye, particularly under dim firelight within cave environments. A newer depiction thus may be superimposed without the direct intention to associate the new depiction with an existing one, *i.e*. the depiction is accidentally placed over an older depiction, due to the older depiction being too faded to distinguish under dim firelight (but possible to see under modern lighting). This may be difficult to discern archaeologically, particularly where chronologies are ambiguous, but must be considered as the base assumption when assessing relationships between depictions. Older depictions that are under calcite flowstone and/or can only be distinguished through modern lighting and the digital enhancement of images (DStretch: Harman, 2008) must be assumed to have been indistinguishable to the artist that produced a superimposed depiction.

## Materials and Methods

### Fieldwork

**Fig. 1:**
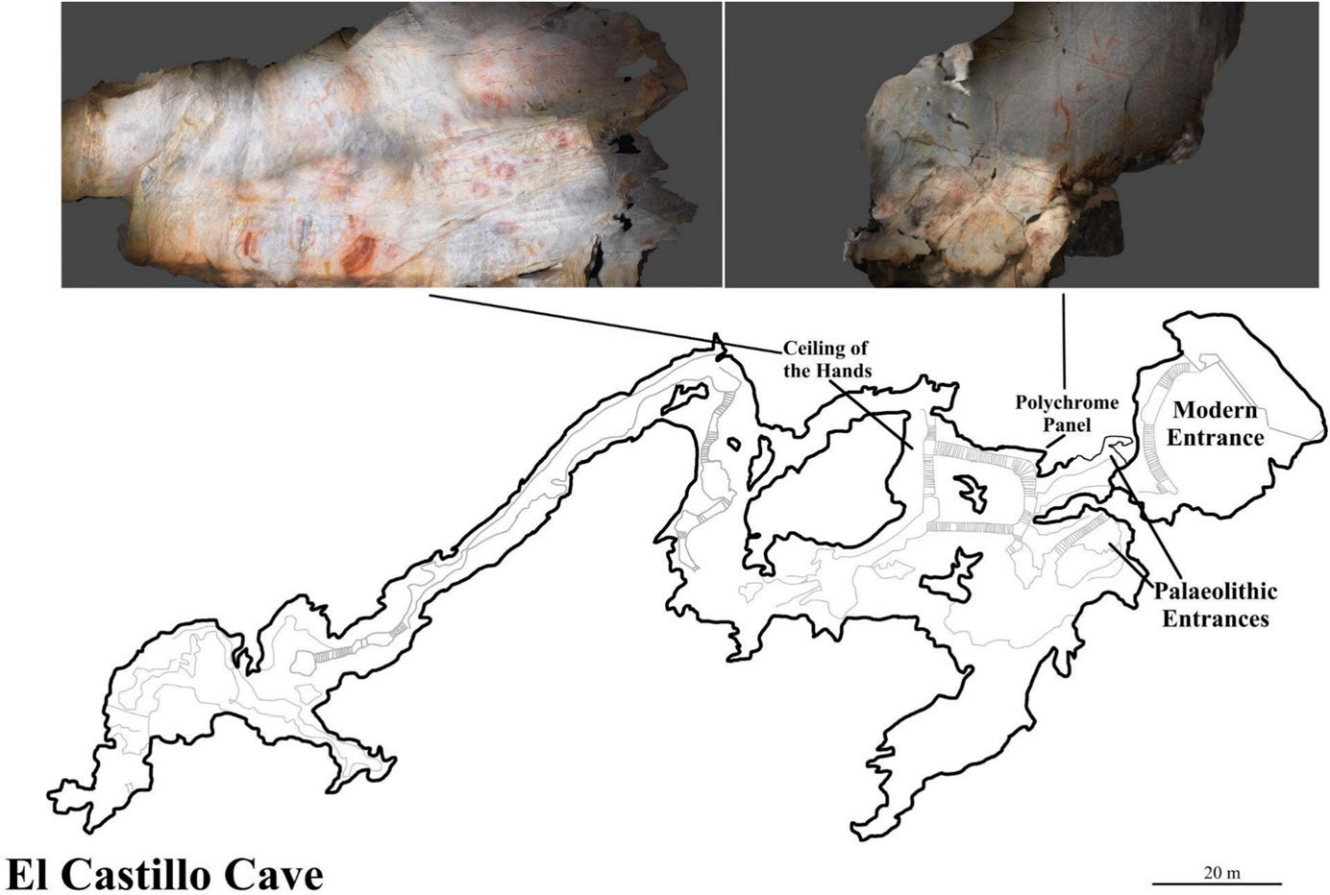
Map of El Castillo cave with location of the two panels focused on for this study

To identify suitable panels with palimpsests of depictions in El Castillo, fieldwork was conducted during May 2023. Panels were selected that conformed to the following basic criteria: (a) superimpositions of multiple motifs, suggesting several periods of interactions; (b) different techniques or artistic mediums for depiction, to help support identifying different phases within the palimpsest; and (c) a significant time depth, to ensure the palimpsest was not the product of one short phase of artistic behaviour. Two panels within El Castillo clearly satisfied these criteria: the *Ceiling of the Hands* and the *Polychrome Panel* (Fig. 1). The superimpositions of these panels have been extensively researched to help establish a relative chronology for the art of El Castillo and inform understandings of the chronology of Upper Palaeolithic art more generally (Alcalde del Río et al., 1911; Breuil & Obermaier, 1935; Breuil, 1952; Leroi-Gourhan, 1965; González Echegaray, 1972; González García, 2001) and to document the precise number of motifs on the panel (*e.g*. Collado Giraldo & Julio García Arranz 2018). For this study, attention was focused on superimpositions within each panel rather than a comprehensive analysis of the number of depictions on the panel. Each motif on the panel was photographed using a Nikon D3500 DSLR camera with an 18–55 mm AF-P VR lens under a cold LED light source. In addition, macro-photos were taken of any areas of clear superimposition on the panel, for example an intersection of several lines from different motifs, to support the clear identification of the order of superimpositions of motifs. Photogrammetric models were produced to capture the dimensionality of the panels and create a high-resolution record of each panel. Photogrammetric images were taken with a Nikon D3500 DSLR camera with an 18–55 mm AF-P VR lens and imported into Agisoft Metashape to produce the photogrammetry models.

### Harris Matrix

In recent years, the Harris Matrix method has been deployed within rock art contexts as one way to evaluate palimpsests of depictions within panels and understand relative chronological phases within the production of the motifs (Harris & Gunn, 2018; Hernando Álvarez, 2010). This approach relies on understanding the relationships between different motifs through evaluating the superimpositions, utilising any available direct dates, and identifying motifs as belonging to the same phase through stylistic and technical similarities (*i.e*. same pigment/tools used to produce the motif) (Harris & Gunn, 2018). This has successfully been used to evaluate chronological phases within a breadth of different rock art contexts (*e.g*. Carden & Miotti, 2020; Feruglio et al., 2019; Gay et al., 2020; Santos et al., 2021; Swart, 2004) and has significant potential for understanding different dialogical relationships that may be present within a palimpsest. Whilst this approach has been critiqued when used to distinguish a sequence within a single rock art tradition (Pearce, 2008), its application here is coarser, used only to distinguish between groups of motifs separated by tens of thousands of years—supported by indirect and direct dating—rather than to suggest a development in a rock art tradition within a few centuries. Particular attention will be paid to stratigraphic relationships in an art palimpsest where depictions from separate phases are directly superimposed.

For the two panels in El Castillo, Harris matrices were produced for groups of superimposed depictions on a panel during fieldwork. These were subsequently analysed, documented with high-resolution and macro-photographs, and, where required, more clearly visualised using DStretch (Harman, 2008) modified images (see supplementary information for full documentation). Depictions were identified as corresponding to the same phase in the Harris Matrix primarily through the order of superimpositions and secondly according to the general colour (*e.g*. black vs. red vs. yellow) of the depiction. Hues within these general colour categories were not evaluated further, as different factors—both taphonomic (*e.g*. overlying calcite) and anthropogenic (*e.g*. binders, pigment processing method)—can affect the hue of pigments on the cave wall (Rifkin, 2012; Velliky et al., 2018). Whilst there are examples of more than one pigment colour possibly being used contemporaneously to produce depictions elsewhere in El Castillo, all depictions that were documented as part of this study appeared to only be produced with one colour, with one exception (a bison on the *Polychrome Panel* that was produced using charcoal and a brown-hued ochre). The general colour therefore served to separate phases; for example, if red depictions consistently underlie yellow depictions on a panel, it was assumed these were two separate phases. The phases identified, unless associated with existing U-Th or AMS radiocarbon dates, were not used to make a chronological assessment beyond what has already been proposed for the chronology of depictions in El Castillo elsewhere (*e.g*. Ripoll-López et al., 2020). Instead, the Harris Matrix only served to represent the different superimpositions that exist within a panel.

## Results and Discussion

### The Polychrome Panel

**Fig. 2:**
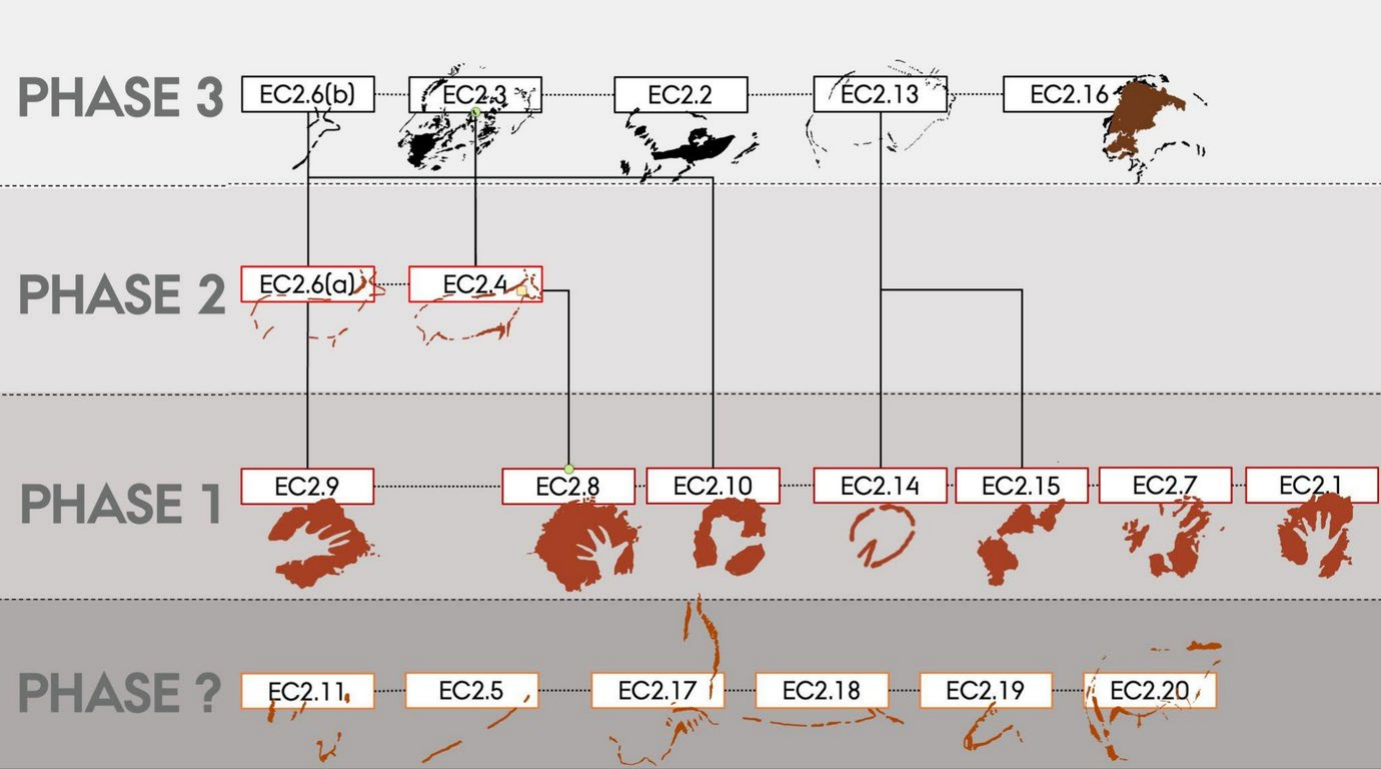
Harris Matrix for the *Polychrome Panel.* Solid lines represent superimpositions of depictions; dashed lines represent possible contemporaneous depictions based on style and pigment hue

**Fig. 3:**
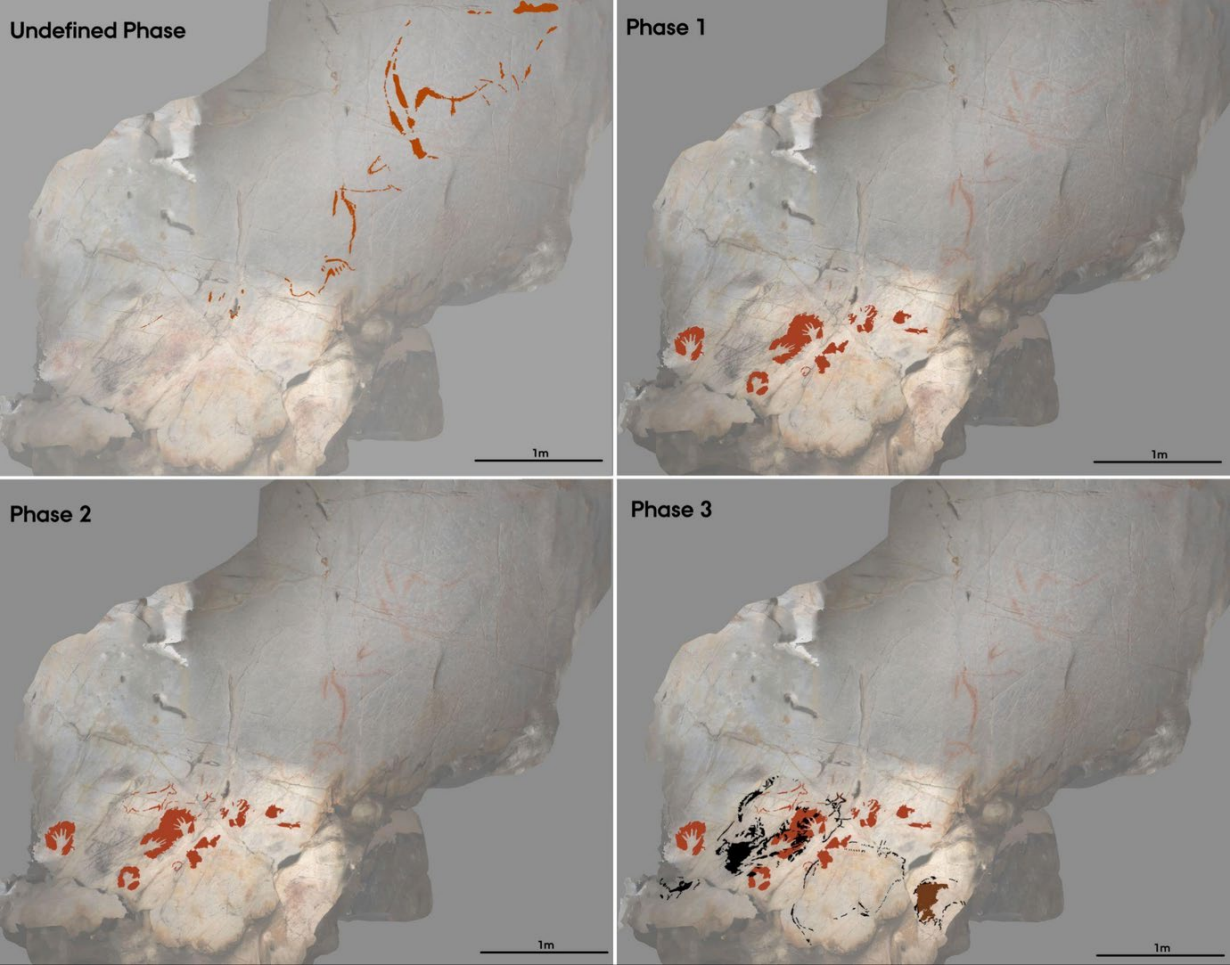
Digital tracings of the *Polychrome Panel*, visualising the four phases of the panel as distinguished by the Harris Matrix

For the *Polychrome Panel*, we documented 20 depictions, consisting of 10 figurative and 10 non-figurative, produced in a range of different ochre hues and charcoal pigments (Table 2). At least three, and possibly four, phases of art production are represented, as characterised by the Harris Matrix (Figs. 1 and 2; see supplementary information). This is a conservative estimate of the phases to this panel, and there may be additional phases to the palimpsest of this panel. Based on stylistic comparisons and AMS radiocarbon dating of the black depictions, it is likely that these phases are separated by considerable time depth. The earliest phase of hand-stencils is difficult to attribute to a particular period and may date to anywhere between the early Aurignacian and the Gravettian. U-Th dates of hand-stencils elsewhere in El Castillo suggest that the production of hand-stencils could have occurred during the early Aurignacian, with a minimum age of 37,300 BP. However, hand-stencils are also generally attributed to the Gravettian period, and it is possible that hand-stencils were produced during different chronological phases in El Castillo (for example, on the *Ceiling of the Hands*). The second phase consists of the two hind depictions, which appear to overlie the hand-stencils (Figs. 2 and 3). These cannot be dated directly but are attributed here to the Gravettian period through stylistic comparisons to portable art pieces. A portable art piece of an engraved hind, deriving from the nearby site of Antoliñako koba and dating to the Gravettian period (31,347–30,650 cal. BP), shares stylistic features with both the two hind depictions in this phase and a Gravettian portable art example from El Castillo (Aguirre & González Sainz, 2011; Ochoa et al., 2019). As Ochoa et al. (2019) note, some of the characteristic features of this art are a simple outline, anatomically incorrect positioning of limbs, and a static position of the animal with legs depicted rigidly. These features are all shared with the hind depictions on the *Polychrome Panel* and further share near-identical features in the rendering of the hind head; the top of the head is open, the ears are positioned in a v-formation, and the head is triangular in shape (see Aguirre & González Sainz, 2011 for the portable art example). The attribution of these hinds to the Gravettian period can be supported through this comparison. The third phase consists of a series of black, charcoal depictions of bison on the panel that can be attributed to the Magdalenian based on a series of AMS radiocarbon dates (Table 1; Valladas et al., 2001). Although these radiocarbon dates are variable, this may be a consequence of either contamination of the samples or poorer resolution, given that the radiocarbon dates were obtained over two decades ago and several samples from the same depiction yielded significantly different ages (see supplementary information). Nevertheless, these dates reflect a younger, possible mid-Magdalenian phase of the panel. There is another phase of depictions on the panel that cannot be placed chronologically due to the lack of superimpositions with the other depictions. This phase consists of the group of red–orange-hued depictions towards the upper right of the panel, and tentatively, based on their similar pigment hue, spatial proximity, and visual similarity in form and technique, these may all correspond to the same phase that is separate to the other three phases identified on this panel. The lack of distinctive stylistic features of these depictions and superimpositions means the chronological attribution of this phase cannot be confidently determined. These depictions are thus grouped as an undefined phase, given that they cannot be securely associated with the other phases identified through the superimpositions on this panel.

**Table 2:** Summary of the depictions on the Polychrome Panel, El Castillo

ID	Type	Description	Pigment	Height (cm)	Width (cm)
EC2.1	Non-figurative	Hand-stencil to the left of panel	Red ochre	27	22
EC2.2	Figurative	Black bison hidden within topography of panel	Charcoal	30	50
EC2.3	Figurative	Large black bison with legs curled under body and superimposed on EC2.3-EC2.8	Charcoal	90	120
EC2.4	Figurative	Red hind (leftmost), superimposed on EC2.5	Red ochre	32	40
EC2.5	Non-figurative	Orange lines underlying EC2.4	Orange-red ochre	1	20
EC2.6	Figurative	Red hind (rightmost), refreshed in black pigment	Red ochre and charcoal	27	41
EC2.7	Non-figurative	Faded hand-stencil (leftmost)	Red ochre	27	20
EC2.8	Non-figurative	Faded hand-stencil (uppermost)	Red ochre	30	28
EC2.9	Non-figurative	Faded hand-stencil (middle, underlying front leg of EC2.3)	Red ochre	43	35
EC2.10	Non-figurative	Faded hand-stencil (underlies rear leg of EC2.3)	Red ochre	22	19
EC2.11	Non-figurative	Smears of pigment to left and right of natural fissure (with ochre inside fissure)	Orange-red ochre	15	5
EC2.12	Non-figurative	Faded hand-stencil positioned underneath EC2.11	Red ochre	30	25
EC2.13	Figurative	Black bison depicted in outline with front legs extended, back legs curled	Charcoal	90	95
EC2.14	Non-figurative	Red oval sign	Red ochre	9	11
EC2.15	Non-figurative	Hand-stencil positioned above EC2.13	Red ochre	25	25
EC2.16	Figurative	Bison depicted with engraved and black pigment outline, partially infilled with brown–red pigment	Charcoal and brown–red ochre	60	50
EC2.17	Figurative	Large bison head in vertical orientation	Orange-red ochre	110	60
EC2.18	Figurative	Partial hind represented by cervical-dorsal line	Orange-red ochre	5	40
EC2.19	Figurative	Probable partial horse head	Orange-red ochre	20	30
EC2.20	Figurative	Large horse with rear, ventral and tail represented	Orange-red ochre	-	-

There are multiple temporal relationships within the palimpsest of this panel that appear to be represented in the superimpositions of depictions. The first pertains to the relationship between the undefined phase of the panel and phase 1–3. Regardless of the chronological relationship between the undefined phase and the rest of the panel, there are some features of the relationship in the palimpsest that can be discussed. The spatial separation of the undefined phase reflects either a deliberate separation of these depictions in this phase away from existing depictions in phases 1–3 or vice versa, representing a moment in time where the spatial—but not visual—separation of these depictions held importance. Despite the spatial separation of the undefined phase, it still is visually associated to the depictions in phases 1–3; the large vertical bison head, for example, is clearly visible when viewing any other depiction in the lower part of the panel. This may reflect a deliberate choice to preserve depictions by not superimposing them, but still maintaining an association between temporally separate depictions; akin to Bailey’s (2007) *spatial palimpsest*.

**Fig. 4:**
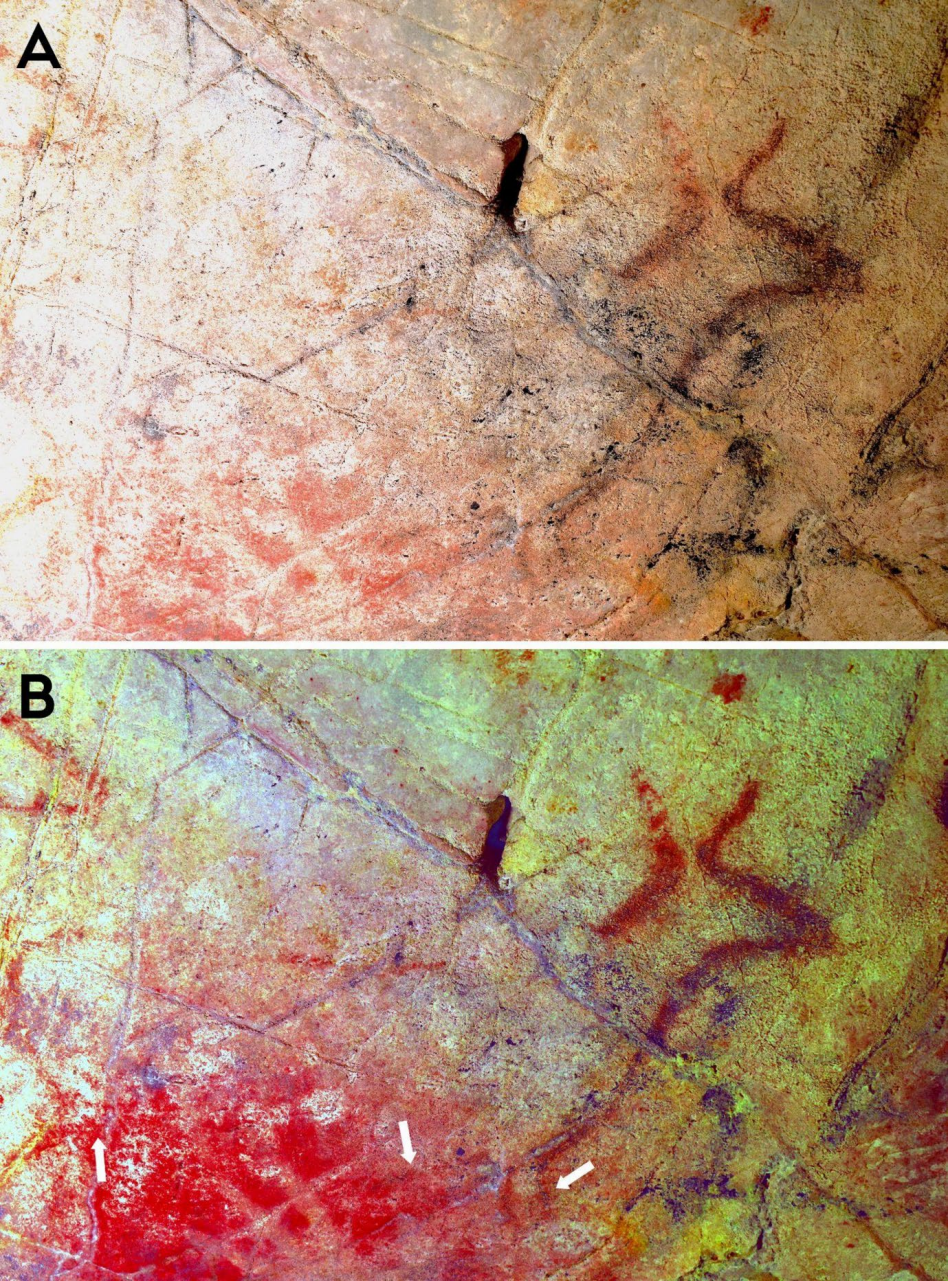
**A** Image of a hind depiction (EC2.6) and **B** DStretch manipulated image in the YRD colour matrix, with white arrows highlighting the superimposition of the ventral line and legs of the hind (EC2.6) on the hand-stencils (EC2.8). Image: Izzy Wisher, courtesy of the Gobierno de Cantabria

Within phases 1–3, there are more nuanced dialogical relationships across time that can be explored. The first relationship is expressed by the interaction between a depiction of a hind (EC2.6; phase 2) and a hand-stencil (EC2.8; phase 1). The placement of the hind depiction appears to intentionally intersect with the hand, with the dorsal line drawn over the fingers of the hand (Fig. 4). This evokes a tactile interaction, giving the impression that the hand is “touching” the hind and tentatively may reflect a desire to capture an engagement between a (possibly ancestral) hand-stencil depiction and the figurative representation of an animal.

**Fig. 5:**
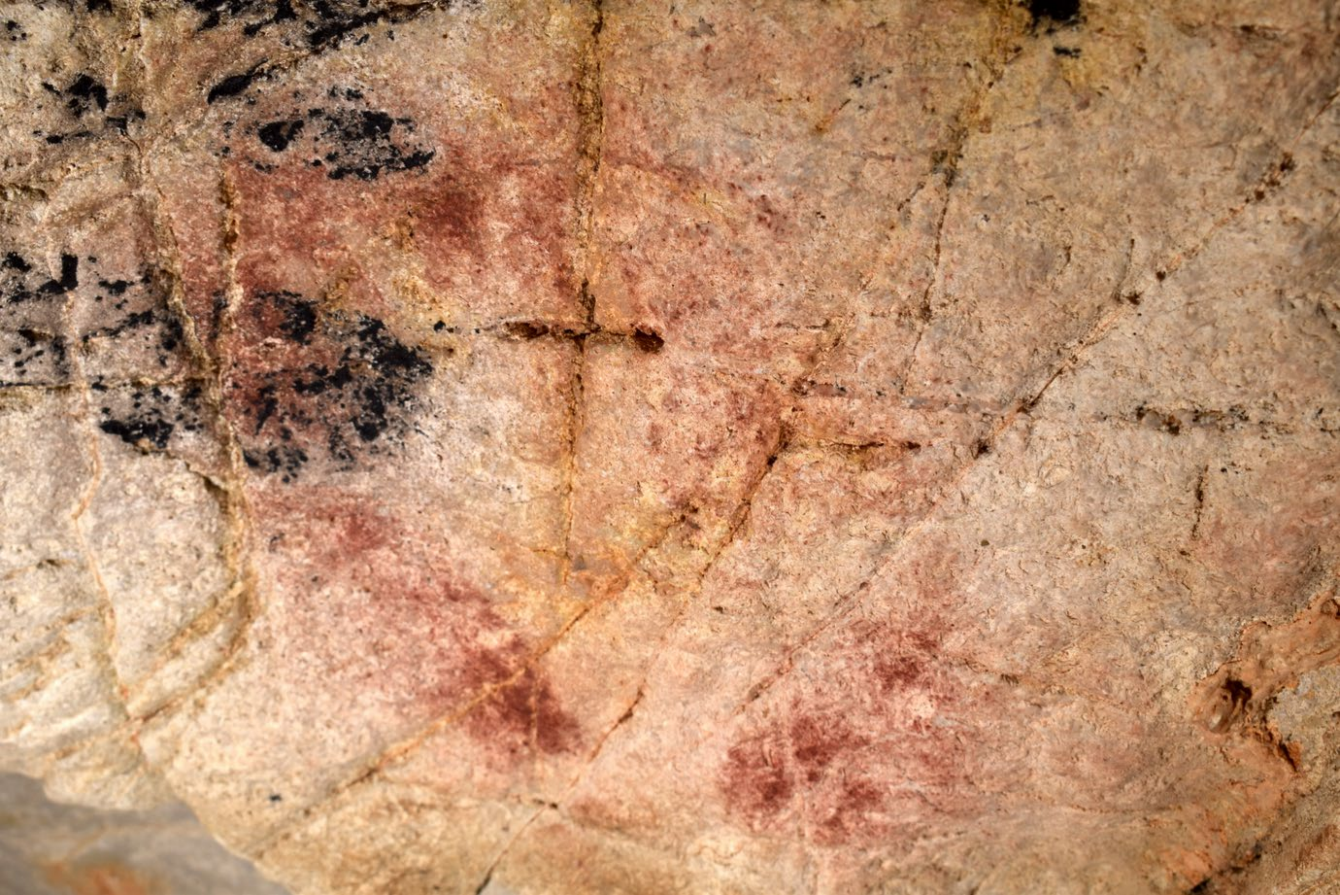
The rear hoof of EC2.3 partially superimposed over the fingers of hand-stencil EC2.10. Image: Izzy Wisher, courtesy of the Gobierno de Cantabria

**Fig. 6:**
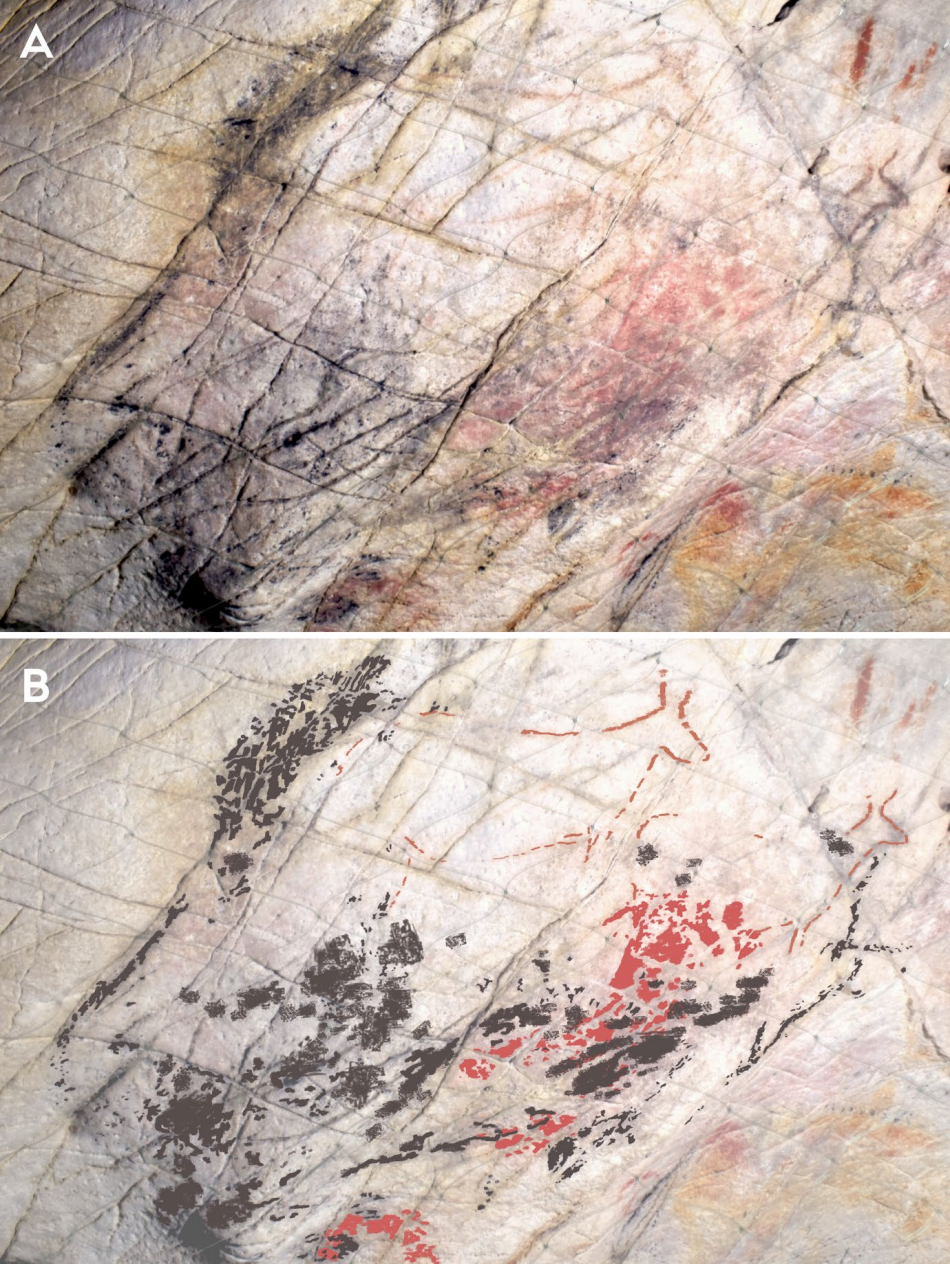
**A** Image and **B** tracing of a bison depiction with its legs curled under its body (EC2.3), and two hind depictions (EC2.4 and EC2.6) visible. Note how the dorsal line of EC2.3 is incomplete, to ensure the hind depictions are not obscured. Image: Izzy Wisher, courtesy of the Gobierno de Cantabria

The dialogical interactions continue with this depiction in the third phase, where a black bison depiction (EC2.3) was superimposed over the two hind depictions (EC2.5 and EC2.6; phase 2) and two hand-stencils (EC2.8 and EC2.9; phase 1) (Fig. 6). This palimpsest reflects a complexity of different relationships. The superimposition of the bison depiction on the two hand-stencils appears to intentionally obscure the hands; the clear visibility of the hands today, despite underlying the black pigment and degradation of the motifs, strongly implies they would have been visible in the Upper Palaeolithic. This intentional, complete obscuration of the hand-stencils may tentatively reflect a deliberate act of intending to imbue the bison depiction with the indexical representations of the past people that produced depictions on this panel or, more drastically, intending to visually “silence” the presence of these hand-stencils. The temporal separation of these depictions, with the hand-stencils being likely Aurigancian-Gravettian in age and the bison dating to the Magdalenian, further supports the direct superimposition of the bison over the hand-stencils was a reactionary and deliberate interaction. The individual(s) that produced the bison depiction would not have had a cultural context for why these hand-stencils were originally produced, but would have recognised the hand-stencils as representing that people were previously present in the cave. The ontological significance of this can only be speculative, but the deliberate interaction between these depictions reflects an intentional choice to fuse the hands within the bison. This relationship is further reflected in the placement of the hoof of the rear leg of the bison over another hand-stencil (EC2.10: Fig. 5). Here, the fingers appear to “touch” the hoof in a similar dialogical relationship to the ventral line of the hind depiction, EC2.6, interacting with another hand-stencil. In this sense, the bison is interacting with multiple hands of past people and integrating these hands within a visual assemblage that recontextualises the hand-stencils as actively fusing and engaging with the bison.

The placement of the bison depiction, EC2.3, to also superimpose the hind depictions may reflect a similar dialogical relationship (Fig. 6); whilst the dorsal line of the bison appears incomplete, it may have originally similarly covered depictions EC2.5 and EC2.6. However, and perhaps juxtaposed to this dialogue, the outline of EC2.6 appears to have been retraced in black pigment, possibly at a similar time as the production of EC2.3 given that the black pigment depictions derive from the same phase. Again, there is a similar separation in time between the hind depictions and the bison depiction, with the former likely deriving from the Gravettian and the latter dating to the Magdalenian, representing a significant temporal separation. The refreshing of the hind depiction in black thus cannot represent a continuation or maintenance of the depiction by the same cultural group; as with the hand-stencils, the original cultural context surrounding the production of the hind depictions would have been lost to the individual(s) producing the bison depiction. The Magdalenian artist(s) thus had no epistemological knowledge to contextualise the hinds, but would be aware that depictions had been produced in the same space by their predecessors and may even have their own cultural value attributed to depictions of hinds. Thus, this action of refreshing the depiction reflects an engagement with an enigmatic, existing depiction on the cave wall where the artist(s) felt compelled to redraw the hind and, in doing so, integrated it within another temporal context. This similarly fuses the hind depiction within the amalgamation of depictions represented by the stratigraphic relationship of bison depiction EC2.3, possibly reflecting an intention to embed ancestral depictions in the form of the bison.

**Fig. 7:**
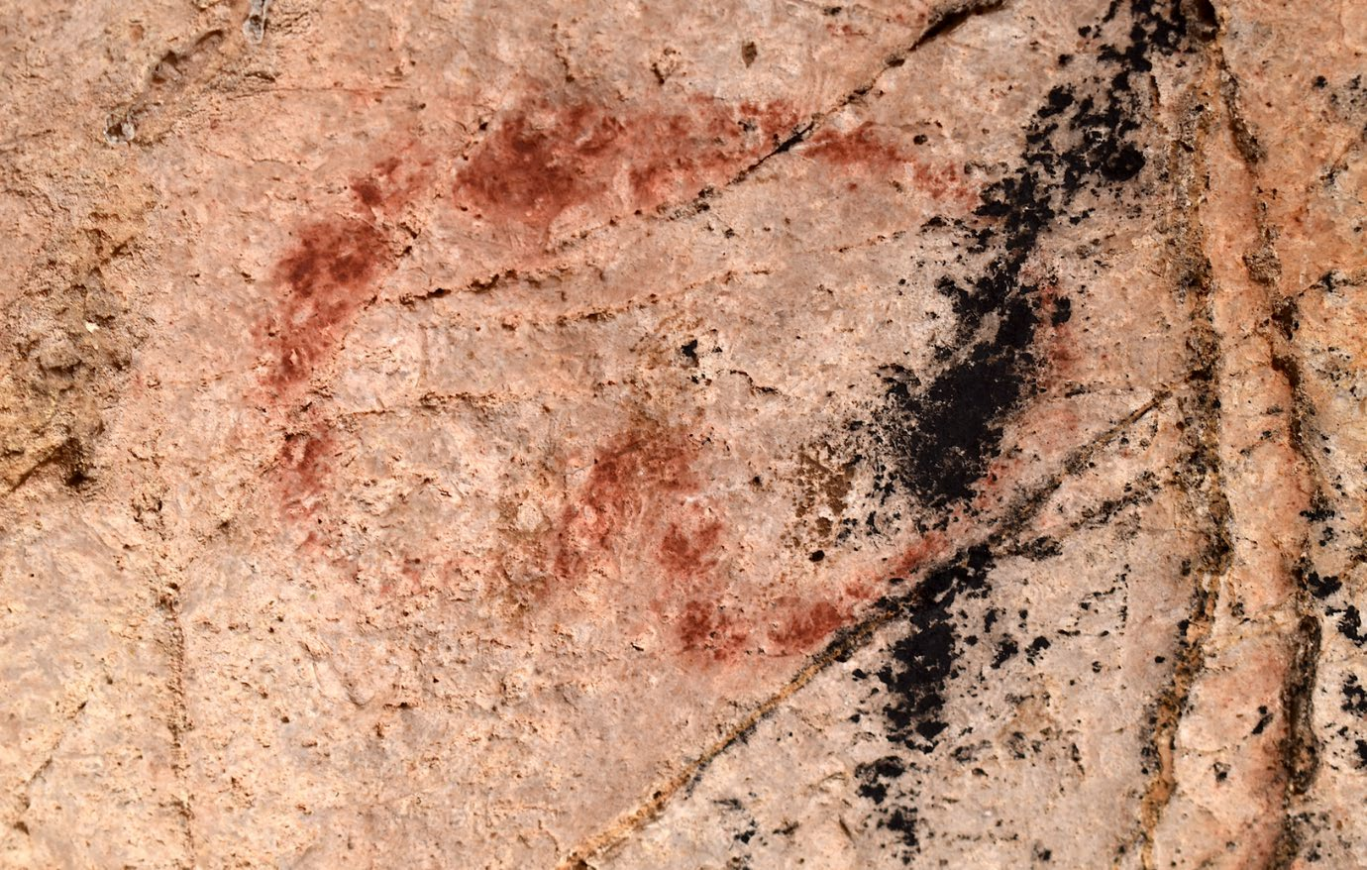
A macro-photograph of depiction EC2.14 and the charcoal dorsal line of EC2.13 as it intersects this depiction. Image: Izzy Wisher, courtesy of the Gobierno de Cantabria

The final relationship in this panel pertains to a small indented, circular motif (often referred to as a “vulva”), EC2.14. This motif appears to have been produced during the same phase as the hand-stencils and thus may similarly significantly predate the Magdalenian charcoal depictions. During the production of the bison depiction EC2.13, the dorsal line appears to have been drawn to deliberately partially intersect this motif, but to a degree where this does not obscure the form of the motif (Fig. 7). This careful interaction between the dorsal line of the bison and the curved outline of the non-figurative motif appears to intentionally associate the two. As with the other superimpositions on this panel, the artist(s) that produced the bison depiction was temporally separated from the original context and meaning that was associated with the non-figurative motif. The deliberate interaction of these two may thus reflect this motif taking on a new meaning, being redefined in association with the bison motif and becoming something new within this Magdalenian context.

### The Ceiling of the Hands

The *Ceiling of the Hands* has a more complex array of depictions which are dispersed across a large section of the cave wall and ceiling. As such, we focused only on areas of the panel that had clear examples of superimposed depictions and thus recorded 71 depictions for this study (Table 3; see supplementary information). This is fewer depictions than have been previously documented for this panel, including approximately only half of the number of hand-stencils than have been documented by the *HandPas* project (Collado Giraldo & Julio García Arranz 2018). However, the focus of the study was not to comprehensively document all depictions on the panel but rather to assess the relationships of the superimpositions, and thus the recorded depictions provide insight into the temporal relationships between depictions.

**Table 3:** Summary of the depictions on the Ceiling of the Hands, El Castillo

ID	Type	Description	Pigment	Height (cm)	Width (cm)
EC1.1	Figurative	Engraved hind with twisted perspective	N/a	31	64
EC1.2	Non-figurative	Engraved lines superimposed on EC1.3	N/a	10	15
EC1.3	Non-figurative	Open quadrangular sign	Red ochre	10	4
EC1.4	Non-figurative	Red vertical line	Red ochre	13	5
EC1.5	Non-figurative	Yellow curved sign positioned above EC1.1	Yellow ochre	7	27
EC1.6	Non-figurative	Red narrow vertical line	Red ochre	10	1
EC1.7	Non-figurative	Yellow quadrangular sign	Yellow ochre	10	32
EC1.8	Non-figurative	Red oval sign with three internal horizontal lines	Red ochre	18	7
EC1.9	Figurative	Engraved striated hind head	N/a	10	17
EC1.10	Non-figurative	Red diagonal line, underlying EC1.9	Red ochre	10	4
EC1.11	Non-figurative	Large red rectangular sign	Red ochre	28	8
EC1.12	Non-figurative	Striated narrow vertical lines in concavity of cave wall	Red ochre	28	9
EC1.13	Non-figurative	Red traces of approximately 8 horizontal lines	Red ochre	11	7
EC1.14	Non-figurative	Partial engraved hind	N/a	10	14
EC1.15	Non-figurative	Traces of three yellow dots	Yellow ochre	10	24 (> 5 cm per dot)
EC1.16	Non-figurative	Open rectangular L-shaped sign	Yellow ochre	20	10
EC1.17	Non-figurative	Long engraved striated lines overlying EC1.16	N/a	45	5
EC1.18	Non-figurative	Long red, diffuse vertical line	Red ochre	47	5
EC1.19	Non-figurative	Long rectangular red sign	Red ochre	50	10
EC1.20	Figurative	Engraved hind overlying EC1.18 and EC1.19	N/a	25	28
EC1.21	Non-figurative	Red dot positioned below EC1.18-EC1.20	Red ochre	2	2
EC1.22	Non-figurative	Yellow rectangular smear	Yellow ochre	15	10
EC1.23	Non-figurative	Engraved lines surrounding yellow rectangular sign	N/a	15	20
EC1.24	Non-figurative	Large composite red sign	Red ochre	35	60
EC1.25	Non-figurative	Engraved L-shaped line superimposed on EC1.24	N/a	27	21
EC1.26	Non-figurative	Faint red lines, possible sign	Red ochre	26	7
EC1.27	Non-figurative	Segmented quadrangular sign	Red ochre	30	7
EC1.28	Non-figurative	Cluster of finger dots	Red ochre	10	5
EC1.29	Non-figurative	Violet-red pigment smear, possible hand	Purple-red ochre	17	20
EC1.30	Non-figurative	Hand-stencil positioned to the right of EC1.24	Red ochre	23	18
EC1.31	Non-figurative	Large yellow faded depiction of a bison	Yellow ochre	55	100
EC1.32	Non-figurative	Hand-stencil to the right of EC1.31	Red ochre	23	24
EC1.33	Non-figurative	Yellow lines, possible sign	Yellow ochre	26	38
EC1.34	Figurative	Large yellow-orange depiction of a bison	Yellow-orange ochre	52	83
EC1.35	Non-figurative	Yellow lines, possible sign	Yellow ochre	18	12
EC1.36	Non-figurative	Yellow lines positioned beneath EC1.34	Yellow ochre	17	31
EC1.37	Figurative	Engraved hind head superimposed on EC1.39	N/a	7	7
EC1.38	Non-figurative	Hand-stencil (uppermost)	Red ochre	24	28
EC1.39	Non-figurative	Hand-stencil	Red ochre	15	29
EC1.40	Non-figurative	Hand-stencil	Red ochre	21	22
EC1.41	Non-figurative	Hand-stencil	Red ochre	27	26
EC1.42	Figurative	Faded yellow bison, facing left and underlying EC1.40–41	Yellow ochre	-	-
EC1.43	Non-figurative	Small red claviform sign	Red ochre	-	-
EC1.44	Non-figurative	Hand-stencil (right hand) in centre and underlying EC1.45	Red ochre	-	-
EC1.45	Figurative	Yellow bison facing left, underlying EC1-46	Yellow ochre	-	-
EC1.46	Figurative	Yellow bovid depiction overlying EC1.44–45	Yellow ochre	-	-
EC1.47	Non-figurative	Partial hand-stencil underlying dorsal line of EC1.45	Red ochre	-	-
EC1.48	Non-figurative	Partial hand-stencil underlying back leg of EC1.45	Red ochre	-	-
EC1.49	Non-figurative	Hand-stencil (leftmost) overlying EC1.45	Red ochre	-	-
EC1.50	Non-figurative	Hand-stencil (centre) overlying ventral line of EC1.45	Red ochre	-	-
EC1.51	Non-figurative	Hand-stencil (rightmost) overlying ventral line of EC1.45	Red ochre	-	-
EC1.52	Figurative	Faded yellow bison, facing right and overlying EC1.54	Yellow ochre	-	-
EC1.53	Figurative	Yellow horse head facing left, overlying EC1.54	Yellow ochre	-	-
EC1.54	Non-figurative	Red-violet hand-stencil, underlying EC1.52–53	Purple-red ochre	-	-
EC1.55	Non-figurative	Faded red-violet hand-stencil to left of EC1.52	Purple-red ochre	-	-
EC1.56	Non-figurative	Series of approx. 25 blown red-violet discs, in curved pattern	Purple-red ochre	-	-
EC1.57	Figurative	Yellow bison facing left overlying EC1.56	Yellow ochre	-	-
EC1.58	Non-figurative	Red hand-stencil to right of EC1.56	Red ochre	-	-
EC1.59	Non-figurative	Red-violet hand-stencil underlying tail of EC1.57	Purple-red ochre	-	-
EC1.60	Non-figurative	Red series of short vertical lines, overlying EC1.59 and EC1.57	Red ochre	-	-
EC1.61	Non-figurative	Two red finger dots above dorsal line of EC1.57	Red ochre	-	-
EC1.62	Non-figurative	Red-violet hand-stencil positioned below EC1.57	Purple-red ochre	-	-
EC1.63	Non-figurative	Red hand-stencil with short fingers	Red ochre	27	26
EC1.64	Non-figurative	4 dots superimposed on EC1.63	Red ochre	10	2
EC1.65	Non-figurative	Long vertical line of finger dots	Red ochre	26	2
EC1.66	Non-figurative	2 finger dots	Red ochre	5	2
EC1.67	Non-figurative	Yellow oval sign	Yellow ochre	14	29
EC1.68	Non-figurative	Possible hand-stencil	Red ochre	31	25
EC1.69	Non-figurative	Possible hand-stencil underlying yellow oval sign	Purple-red ochre	23	18
EC1.70	Non-figurative	Series of small violet-red dots	Purple-red ochre	-	-
EC1.71	Non-figurative	Violet-red hued hand-stencil underlying the head of EC1.52	Purple-red ochre	-	-

**Fig. 8:**
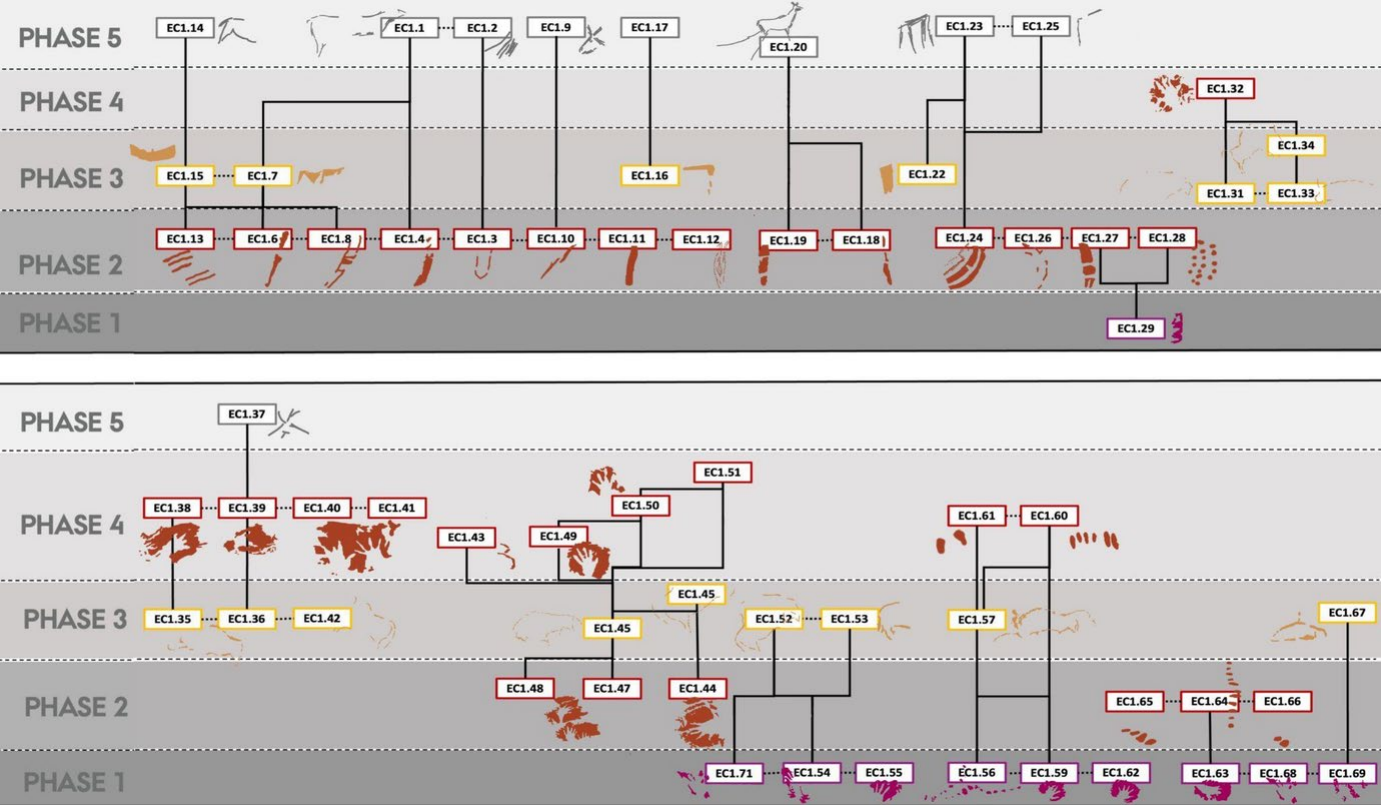
Harris Matrix for the *Ceiling of the Hands*. Superimpositions are represented by solid lines between depiction labels. Depictions that are of the same style and are depicted adjacent to each other are represented by a dashed line

**Fig. 9:**
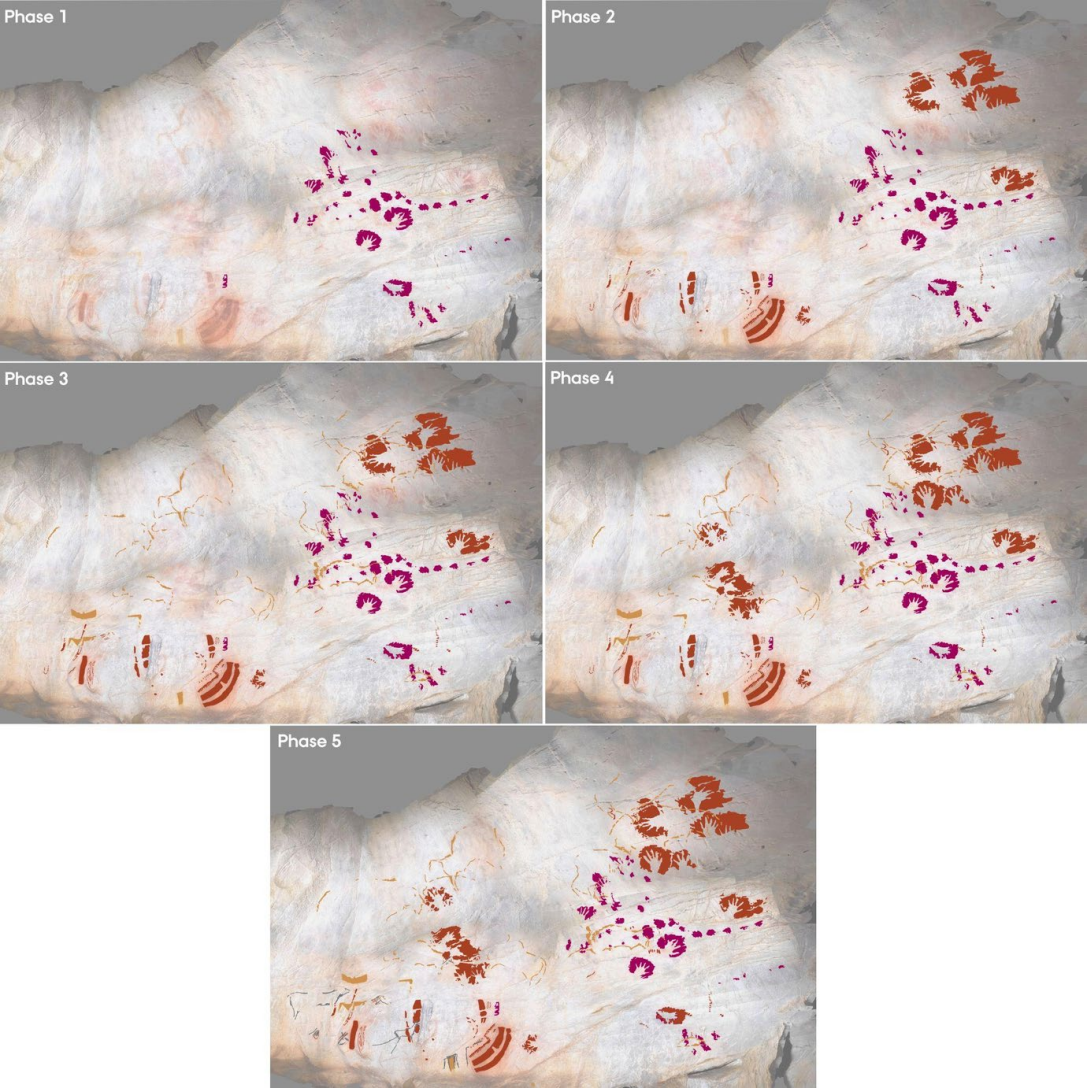
Digital tracings of the *Ceiling of the Hands*, visualising the five phases of the panel as distinguished by the Harris Matrix

The Harris Matrix represents a minimum of five phases to the superimpositions of this panel, identified through the stratigraphic order of the depictions (Figs. 8 and 9); as with the *Polychrome Panel*, this is a conservative estimate. The first phase represents the oldest graphic phase represented in El Castillo, consisting of hand-stencils and discs produced using a purple-red coloured ochre. These have been previously dated by U-Th to a minimum age of c.40,000 BP (Table 1; Pike et al., 2012). Whilst there are some doubts over the reliability of this dating (Sauvet et al., 2017), it is possible that this phase dates to the Aurignacian and, in any case, given that these purple-red depictions consistently underlie all other depictions, represents the oldest phase on the panel. The second, third, and fourth phases are difficult to attribute to specific periods, and it is likely that there is not an extensive time depth separating these phases. Both the second and fourth phases are characterised by the use of a vibrant red ochre hue and are considered separate phases through respectively under- or overlying the yellow motifs that characterise the third phase (see supplementary information). Further, there is a horse depiction close to the *Ceiling of the Hands* that utilises both red and yellow ochre in its outline, suggesting these two pigments may have been used contemporaneously, even if the use of these pigments is constrained to specific depictions, with no depiction appearing to be produced with more than one pigment colour on the *Ceiling of the Hands.* It is probable, therefore, that these three phases are more tightly constrained in time, particularly considering the presence of hand-stencils produced with a similar ochre hue in both the second and fourth phases. This would also align with Ruiz-Redondo’s (2012) assessment that there are only three phases represented on this panel; phases 2–4 were produced in a short time period representing one broad temporal phase. However, given that considering these three phases as one larger phase would subsume the superimposed relationships between depictions from these three phases on the panel, which are the central focus of the study, these were considered as separate here. Whilst it is difficult to attribute a chronological assessment to these phases, as suggested by Ripoll-López et al. (2020), the stylistic features and dynamicity of the figurative motifs and the complexity of non-figurative motifs are consistent with late Gravettian-early Solutrean art. The final phase of this panel consists of a series of small, engraved motifs, predominately depicting hinds. These are characteristic of the possible “striated” hind depictions that are generally attributed to the early Magdalenian (Ibero et al., 2024; Rivero et al., 2019) and, in any case, represent the final phase of the art. The *Ceiling of the Hands* thus appears to have an extensive temporal depth to its palimpsest of art, which may stretch from the early Aurignacian to the late Magdalenian.

The temporal depth and complexity of the panel’s palimpsest mean several different dialogical relationships between depictions across time are represented, occurring both at the macro- and micro-scale. At the most general scale, there appears to be a clear cumulative palimpsest that emerges through the addition of hand-stencils across three distinct phases. As characterised by Sapwell (2017), these kinds of patterns within palimpsests may reflect the continuation of an idea across time. The presence of one motif—a hand-stencil—encourages the addition of the same motif, ensuring that the “message” of the motif is maintained, but also bolstering its potency. The extensive temporal depth of the cumulative hand-stencil palimpsest—extending from the early Aurignacian to possibly the late Gravettian/early Solutrean—alludes to a cultural idea persisting in some regard across tens of thousands of years. Whilst the phases of this panel are not continuous and a significant time passed between phases 1 and 2, a hand-stencil is a recognisable and evocative motif with many immediate connotations; it is indexical of human presence, representing the near-literal embodiment of a person within a place. This idea of situating and embodying oneself within the same cave space is thus maintained across time through this aspect of the palimpsest, regardless of the particular nuanced ontological interpretations held by each individual as they added to this palimpsest.

**Fig. 10:**
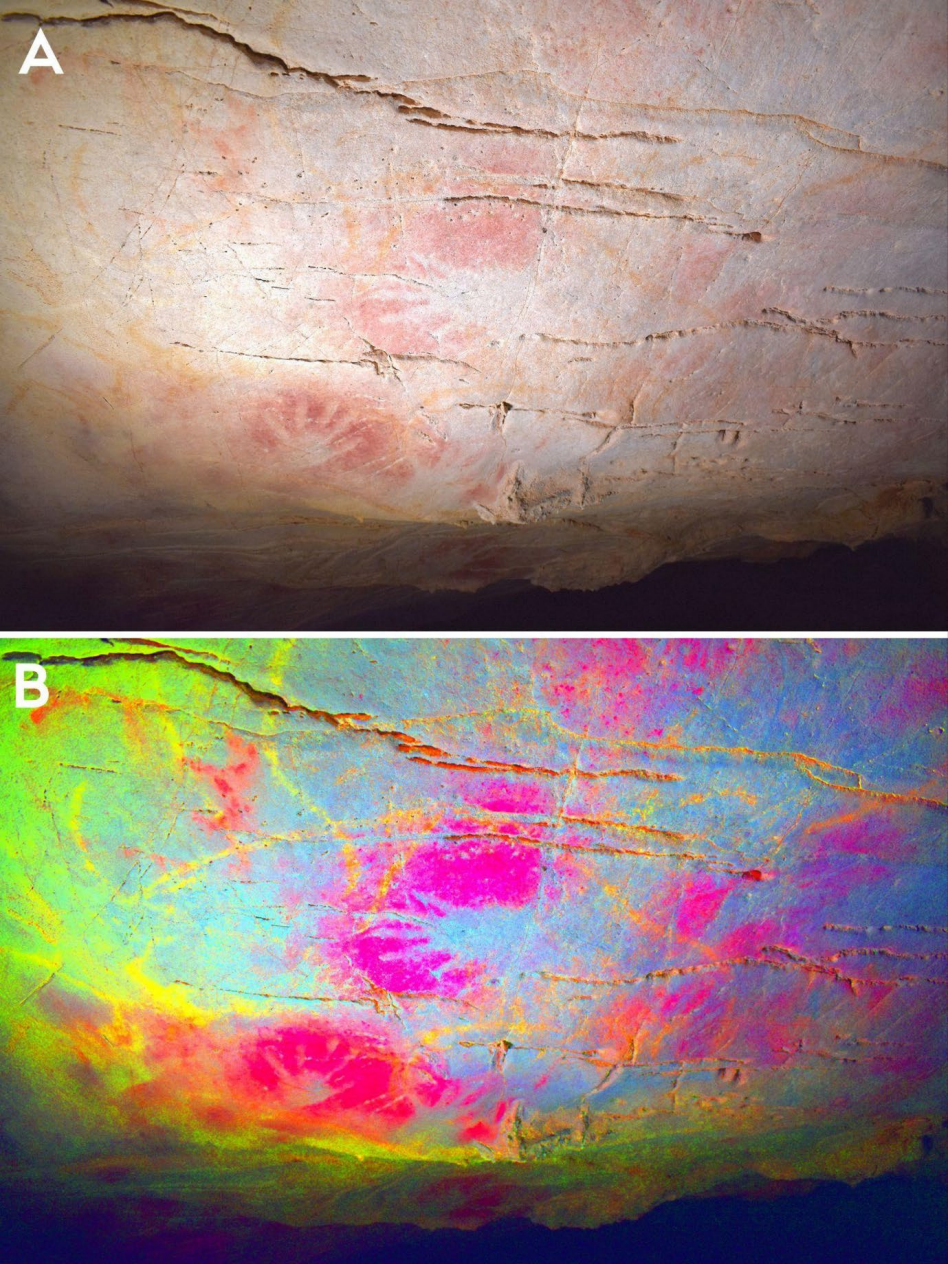
Depiction EC1.45 and the surrounding hand-stencils, exhibiting different stratigraphic relationships (both under- and overlying the bison depiction). **A** High-resolution image of the depiction. **B** DStretch manipulated image to better visualise the superimpositions. Image: Izzy Wisher, courtesy of the Gobierno de Cantabria

**Fig. 11:**
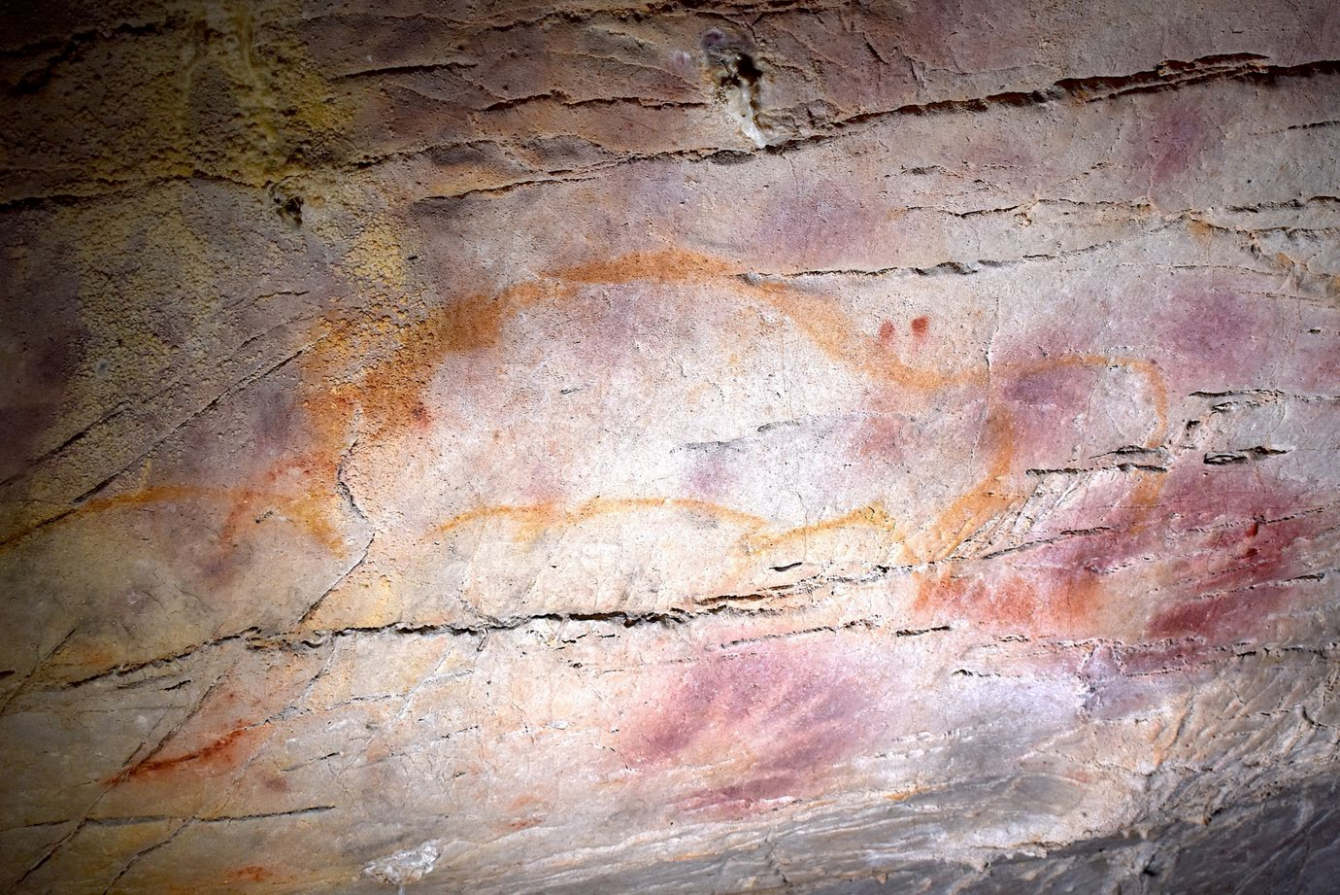
Bison depiction EC1.57 and tactile marks of hand-stencils and fingers associated with this outline. Note the two finger dots on the dorsal line, and the series of short lines made with the fingers towards the rear of the depiction. Image: Izzy Wisher, courtesy of the Gobierno de Cantabria

There are additional interactions between motifs across different phases in this panel that allude to more discrete dialogues occurring between old and new depictions. Akin to the superimposition of a hind depiction over a hand-stencil in the *Polychrome Panel*, there appear to be deliberate interactions between hand-stencils and animal outlines on the *Ceiling of the Hands* that are most clearly observed in the group of depictions to the upper right of the panel (Fig. 9)*.* The first phase within this group of depictions consists of red hand-stencils that may have been added to this area due to the presence of existing hand-stencils from the earliest phase of this panel. Yellow bison depictions seem to be intentionally superimposed over the hand-stencils from both phase 1 and phase 2 and thus may have been depicted to ensure an interaction between the bison and hand-stencils (Fig. 10): the dorsal line of EC1.52 traces over the fingers of EC1.54; the ventral line of EC1.45 is drawn over the thumb of EC1.44; and the rear of EC1.45 follows the shape of the hand of EC1.47. This idea is continued in the fourth phase, where hand-stencils are further added to the composition of hands and bison, superimposed over different features of the bison depictions. In the top right group of depictions, hand-stencil EC1.49 interacts with the bison depiction EC1.45; the hand is placed over the front leg of the bison and produced to not obscure the bison but add to this composition. Similarly, hand-stencil EC1.32 is placed over the front leg and head of the possible bison depiction EC1.33. This theme of associating hands with bison depictions both appears to persist across several phases of the palimpsest—though these may have occurred within a relatively constrained timespan—possibly reflecting some significance of tactilely interacting with the figurative motifs and preserving this interaction. This is further suggested by the addition of finger marks or dots over the dorsal line and close—possibly superimposed—to the rear of bison depiction EC1.57 (Fig. 11). The intentional additive relationships between hands, fingers, and bison thus suggest this was a deeply meaningful theme for the production of these motifs, whereby the bison motifs are represented as being engaged with by indexes of humans. In this sense, they evoke the blurring of animal-human boundaries; visually, human hands and bison profiles cannot be distinguished but fuse together.

**Fig. 12:**
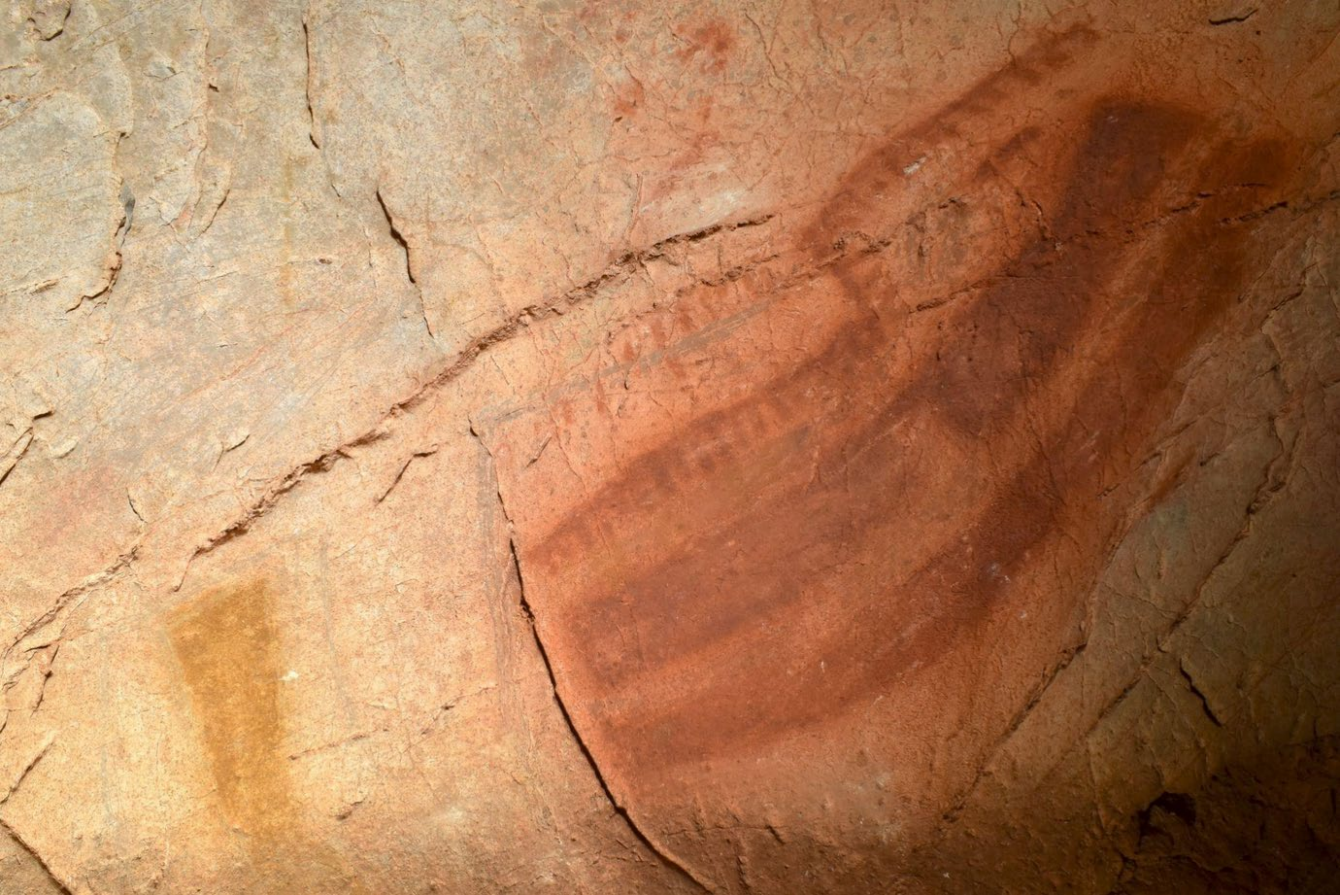
Depictions EC1.22–EC1.24. The large red composite figure is integrated with the yellow rectangular motif through the addition of engraved lines. Image: Izzy Wisher, courtesy of the Gobierno de Cantabria

Other temporal relationships between depictions can be observed within the lower left group of depictions on the panel. The engraved lines superimposed on a large, red non-figurative motif (EC1.24) allude to a reconceiving of pre-existing motifs, where the addition of new components to the motif both simultaneously brings the depiction into a present context and redefines it in a new way (Fig. 12). The engraved lines also appear to integrate EC1.24 with an adjacent yellow rectangular motif (EC1.22) that likely corresponds to a different phase of the palimpsest as identified by the Harris Matrix. However, the extent of the temporal separation of these elements in the palimpsest is unknown. The bringing together of these two depictions with the engraved lines may represent a new meaning being attributed to these motifs, either deliberately changing or manipulating a known and existing cultural meaning of the depictions or attributing a new meaning to the pre-existing motifs, whose original meaning may have been unknown to the artist producing the engraved lines. The dynamics within this group of depictions—regardless of the temporal separation of each element—thus reflect the shifting nature of meaning attributed to specific motifs on this panel.

**Fig. 13:**
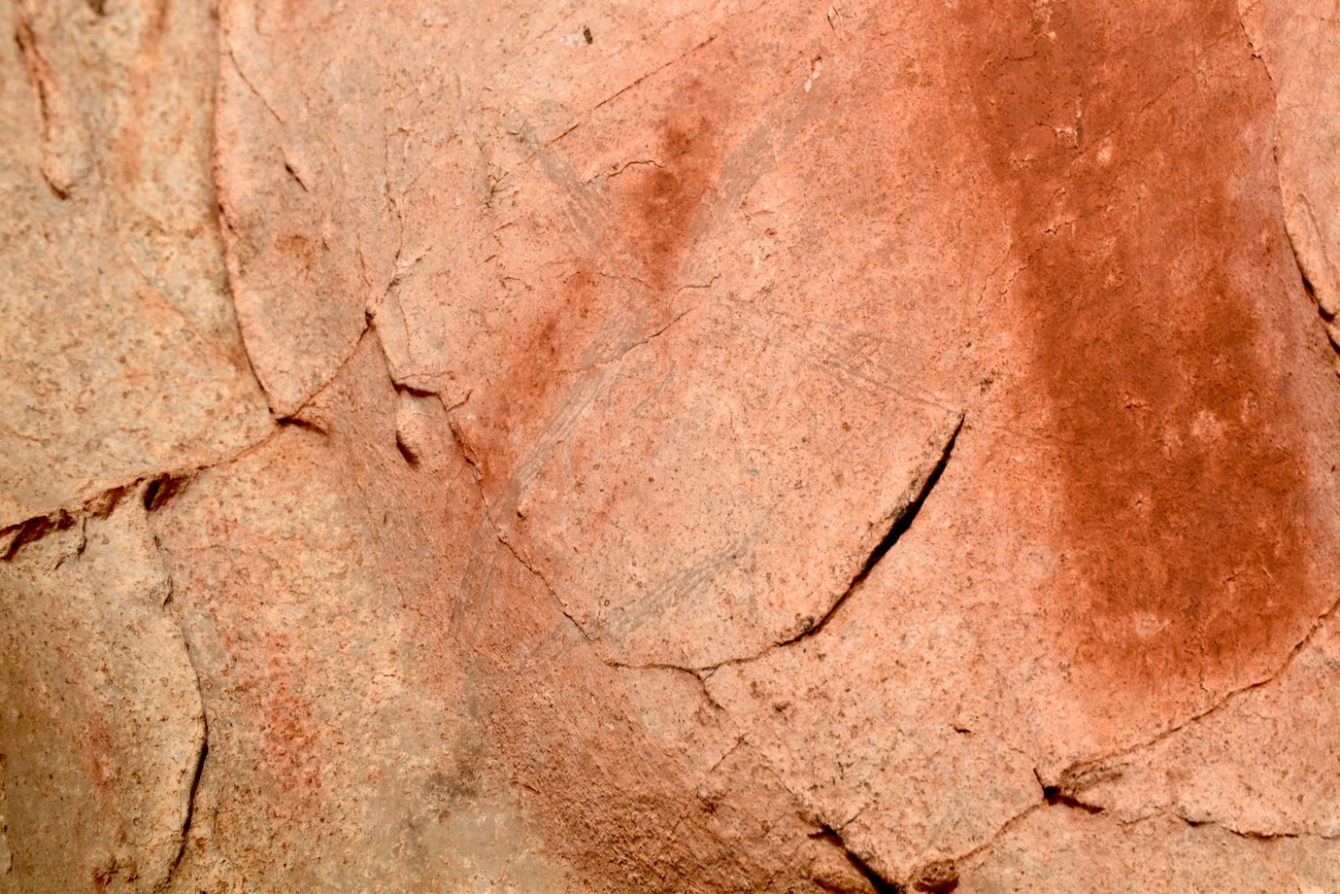
Depiction EC1.9 of an engraved hind head superimposed over two red, non-figurative lines (EC1.10). The dorsal line and ears of the engraved hind head appear to be shaped around the two red lines, following their curvature. Image: Izzy Wisher, courtesy of the Gobierno de Cantabria

**Fig. 14:**
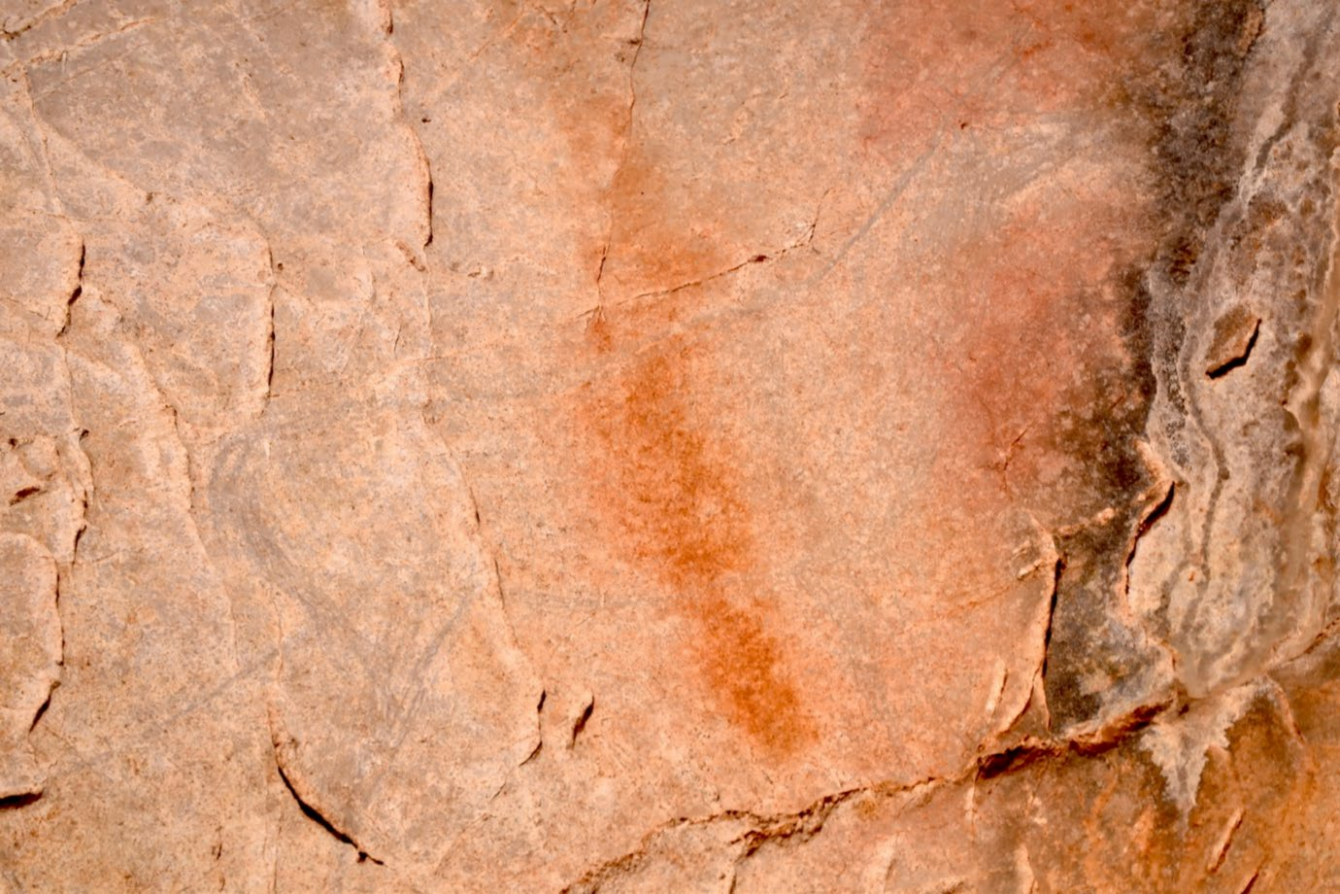
Depiction EC1.20 of an engraved hind outline, where the head appears to “fit” within a gap of the non-figurative motif EC1.19. Image: Izzy Wisher, courtesy of the Gobierno de Cantabria

There is also a close spatial relationship and superimposition of the engraved hind depictions that represent the final phase of the panel and the cluster of non-figurative motifs to the lower left of the panel. All of the engraved hinds are placed to overlie the red and yellow motifs but, by virtue of the technique chosen to render the hinds, do not appear to intentionally erase or obscure these motifs. The red and yellow non-figurative motifs are also clearly visible in the modern day and thus would have been similarly visible when the hinds were added. In this sense, the addition of the engraved hinds must reflect an intentional additive relationship, but one that appears perhaps more subtle or playful than other additive relationships explored in this panel and the *Polychrome Panel.* For example, two of the superimposed engraved hinds appear to follow or “fit” within the form of the underlying red motif (Figs. 13 and 14), suggesting a playful interaction where the engraved depictions were intended to visually appear as naturally following or blending with the non-figurative motif, akin to the engraved “additions” to the yellow and red motifs, to blur these depictions together as a larger composition.

## Conclusion

*“It is the durable properties of the material universe that give to human awareness a sense of time extending beyond individual lives and perceptions”* (Bailey, 2007).

The reception of existing motifs on panels in El Castillo provides insight into the ontological perception of the past by Upper Palaeolithic peoples. The active engagement and interaction with these motifs imply a presencing of the past, bringing pre-existing motifs into a new context and engaging with them in dialogue with other motifs. The majority of the relationships explored within the panels at El Castillo pertain to intentional additive relationships, where there is a deliberate association of new depictions to existing depictions. This often relates to the superimposition of motifs on hand-stencils, producing a visual assemblage that evokes a tactile engagement between the past and present motifs. Although speculative, the attraction to superimposing depictions over existing hand-stencils—that were likely produced in a temporally separate context—may relate to the indexical nature of the stencils; they represent the presence of past humans within the cave. Within the intentional additive relationships in the panels of El Castillo, there were also examples where depictions were incorporated into a new depiction, creating a composite motif that recontextualises the older motif on the panel. These reflect a deliberate and active engagement with the past by Palaeolithic artists and thus have implications for understanding the ontological context within which these motifs were produced over time. The relationships between superimposed motifs within these cave art palimpsests elucidate a persistence of certain ideas over thousands, or tens of thousands, of years: the significance of embodying oneself within place and the tactile relationship between hand-stencils and animals. Implicitly, the lack of relationships that seem to deliberately erase a pre-existing motif perhaps also reflects a desire to maintain the past and recontextualise it within the present.

The interaction of motifs within the rock art palimpsests of El Castillo cave thus provides nuanced insights into the way Palaeolithic artists conceived of and engaged with their own ancestral past, evidenced in the material traces of superimposed motifs. This reflects just one relational process that occurred in the multifaceted making of cave art. Other dimensions—the spatial context of the cave (Ochoa, 2017), psychological responses to suggestive natural features of cave walls (Hodgson, 2008; Wisher et al., 2023, 2024), acoustics (Fazenda et al., 2017; Till, 2014), and firelight (Wisher & Needham, 2023)—all coalesced and had an active role within cave art making. Exploring the dialogical relations between motifs in a palimpsest does not intend to diminish these dimensions, but provide one lens through which to understand the way Palaeolithic artists engaged and related to cave art.

This paper contributes to reconciling traditional approaches to rock art palimpsests, typically focused on identifying chronological phases, and a more recent theoretical shift that emphasises the active character and specific effects of individual action in the past. The framework presented in this paper thus facilitates the examination of relationships within cave art palimpsests, allowing for these to be understood in an active way as representing deliberate engagements Palaeolithic artists had with their own past. The presence of existing motifs on a cave wall appears to have been conceptualised by Palaeolithic artists in a way that these active engagements thus imply a persistence of certain ideas within the ontological meanings associated with producing cave art depictions. It is perhaps unsurprising that in the cases discussed in this paper, these often concerned hand-stencils; these are inherently indexical of humans and unequivocally represent the presence of other (ancestral) people within a place. Exploring relationships within cave art palimpsests in this way can thus facilitate interpretations that appreciate how the meaning of particular panels may have shifted over time and become recontextualised within new contexts.

## Supplementary Information

Below is the link to the electronic supplementary material.

ESM 1 (PDF 102 MB)

## Data Availability

No datasets were generated or analysed during the current study.

## References

[CR1] Aguirre, M., & González Sainz, C. (2011). Placa con grabado figurativo del Gravetiense de Antoliñako koba (Gautegiz-Arteaga, Bizkaia). *Kobie,**30*, 43–62.

[CR2] Alcalde del Río, H., Breuil, H., Sierra, L. (1911) *Les cavernes de la Région Cantabrique,* Mónaco.

[CR3] Anati, E. (1999). The rock art of the Negev Desert. *Near Eastern Archaeology,**62*(1), 22–34.10.2307/3210720

[CR4] Bailey, G. (2007). Time perspectives, palimpsests and the archaeology of time. *Journal of Anthropological Archaeology.,**26*(2), 198–223. 10.1016/j.jaa.2006.08.002

[CR5] Balbín Behrmann, R., & Alcolea González, J. J. (2013). Tito Bustillo en Fechas. In P. León Gasalla (Ed.), *Excavaciones arqueológicas en Asturias 2007–2012: En el centenario del descubrimiento de la caverna de la Peña de Candamo*(pp. 555–569). Oviedo, Principado de Asturia.

[CR6] Barachhini, L., & Monney, J. (2018). Past images, contemporary practices: Reuse of rock art images in contemporary San art of Southern Africa. In B. David & I. J. McNiven (Eds.), *The Oxford Handbook of the Archaeology and Anthropology of Rock Art* (pp. 1043–1065). Oxford University Press.

[CR7] Bayarri, V., Castillo, E., Ripoll, S., & Sebastián, M. A. (2021). Improved application of hyperspectral analysis to rock art panels from El Castillo Cave (Spain). *Applied Sciences,**11*, 1–18.

[CR8] Bednarik, R. G. (2010). Pleistocene rock art in Australia. *Anthropos,**105*, 1–10.

[CR9] Bednarik, R. G. (2014). *Pleistocene Paleoart of Europe. Arts,**3*(2), 245–278. 10.3390/arts3020245

[CR10] Bernaldo de Quirós, F., Maíllo-Fernández, J.-M., Castaños, P., & Neira, A. (2015). The Gravettian of El Castillo revisited (Cantabria, Spain). *Quaternary International,**359–360*, 462–478. 10.1016/j.quaint.2014.07.060

[CR11] Blundell, V., Woolagoodja, D., Oobagooma, J., & Umbagai, L. (2018). Visiting Gonjorong’s Cave. In B. David & I. J. McNiven (Eds.), *The Oxford handbook of the archaeology and anthropology of rock art* (pp. 1–29). Oxford University Press.

[CR12] Brady, L. M. (2016). Contemporary indigenous relationships to archaeological features: Agency, affect, and the social significance of rock art. *Heritage and Society,**9*(1), 3–24. 10.1080/2159032X.2016.1246153

[CR13] Brady, L. M., Bradley, J. J., & Kearney, A. J. (2016). Negotiating Yanyuwa rock art: Relational and affectual experiences in the southwest Gulf of Carpentaria, northern Australia. *Current Anthropology,**57*(1), 28–52. 10.1086/684683

[CR14] Breuil, H. (1952). *Quatre cents siècles d’art pariétal*. Montignac: Max Fourny.

[CR15] Breuil, H., & Obermaier, H. (1912). Les premiers travaux de l’Institut de Paléontologie Humaine. *L’anthropologie,**23*, 1–27.

[CR16] Breuil, H. & Obermaier, H. (1912b). Fouilles de la grotte du Castillo (Espagne). *XIV Congrés International d'Anthropologie et d'Archéologie Préhistorique* XIV session, t.I (Geneve 1912): 361–362.

[CR17] Breuil, H. &Obermaier, H. (1913). Institut de Paléontologie Humaine. Travaux exécutés en 1912. *L'Anthropologie XXIV*: 1–6.

[CR18] Breuil, H. & Obermaier, H. (1914) Institut de Paléontologie Humaine. Travaux de l'année 1913. Travaux en Espagne. *L'Anthropologie XXV:* 233–253.

[CR19] Breuil, H., & Obermaier, H. (1935). *La cueva de Altamira en Santillana del Mar*. Tipografía de Archivos.

[CR20] Breuil, H., Obermaier, H. & Alcalde del Río, H. (1913). *La Pasiega à Puente Viesgo (Santander) (Espagne)*. Chêne: Imprimerie Vve A.

[CR21] Cabrera Valdés, V. (1984). *El Yacimiento de La Cueva de El Castillo (Puente Viesgo, Santander)*. Conseio Superior de Investigaciones Científicas.

[CR22] Cabrera, V., Bernaldo De Quirós, F., Maíllo, J.M., Pike-Tay, A. & Garralda, M.D. (2005). Excavaciones en el Castillo: veinte años de reflexiones. In: R. Montes & J.A. Lasheras (eds.) *Actas de la reunión científica Neandertales cantábricos, estado de la cuestión. Monografías 20* (pp. 505–526)*.* Santander: Museo de Altamira.

[CR23] Carden, N., & Miotti, L. (2020). Unravelling rock art palimpsests through superimpositions: The definition of painting episodes in Los Toldos (southern Patagonia) as a baseline for chronology. *Journal of Archaeological Science: Reports,**30*, Article 102265. 10.1016/j.jasrep.2020.102265

[CR24] Cartailhac, E. & Breuil, H. (1906). *La Caverne d’Altamira à Santillane près Santander (Espagne)*. Chêne: Imprimerie de Monaco.

[CR25] Chapman, J. C. (1994). The living, the dead and the ancestors: Time, life cycles and the mortuary domain in later European prehistory. In J. Davies (Ed.), *Ritual and Remembrance: Responses to Death in Human Societies* (pp. 40–85). Sheffield Academic Press.

[CR26] Clottes, J. (2001). *La grotte Chauvet. L’art des origins*. Seuil, Paris.

[CR27] Clottes, J. (2012). Ritual cave use in European Paleolithic caves. In: H. Moyes (Ed). *Sacred darkness: A global perspective on the ritual use of caves*(pp. 15–26). University Press of Colorado, Boulder.

[CR28] Collado Giraldo, H. & Julio García Arranz, J. (2018) Cueva de El Castillo (Puente Viesgo). In: H. Collado (Ed.), *Handpas. Manos del pasado: Catálogo de representaciones de manos en el arte rupestre paleolítico de la Península Ibérica* (pp. 127–227). Merida: Junta de Extremadura.

[CR29] Colwell, C. (2022). A palimpsest theory of objects. *Current Anthropology,**63*(2), 129–157. 10.1086/719851

[CR30] Corchón, M. S., Garate, D. G., & Rivero, O. (2017). *La Caverna de la Peña de Candamo (Asturias): 100 años después de su descubrimiento*. Ediciones Universidad Salamanca.

[CR31] Davidson, I. (2023). Humans making history through continuities and discontinuities in art. *Cambridge Archaeological Journal,**33*(4), 637–654. 10.1017/S0959774323000057

[CR32] Díaz-Guardamino, M. (2020) Rock art as process: Iberian Late Bronze Age ‘warrior’ stelae in-the-making. In: I.M. Back Danielsson & A.M. Jones (eds.) *Images in-the-making: Art, process, archaeology (social archaeology and material worlds* (pp. *.* Manchester: Manchester University Press.&nbsp; 10.7765/9781526142856.00014

[CR33] Dibble, H. L., Holdaway, S. J., Lin, S. C., Braun, D. R., Douglass, M. J., Iovita, R., McPherron, S. P., Olszewski, D. I., & Sandgathe, D. (2017). Major fallacies surrounding stone artifacts and assemblages. *Journal of Archaeological Method and Theory,**24*, 813–851. 10.1007/s10816-016-9297-8

[CR34] Domingo, I., Smith, C., & May, S. (2017). Etnografía y Arte rupestre: Potencial, perspectivas y ética. *Complutum,**28*(2), 285–305. 10.5209/CMPL.58431

[CR35] Eisenberg-Degen, D., Nash, G. H., & Schmidt, J. (2016). Inscribing history: The complex geographies of bedouin tribal symbols in the Negev Desert, Southern Israel. In L. M. Brady & P. S. C. Tacon (Eds.), *Relating to Rock Art in the Contemporary World* (pp. 157–188). University Press of Colorado.

[CR36] Fazenda, B., Scarre, C., Till, R., Pasalodos, R. J., Guerra, M. R., Tejedor, C., Ontañón Peredo, R., Watson, A., Wyatt, S., García Benito, C., Drinkall, H., & Foulds, F. (2017). Cave acoustics in prehistory: Exploring the association of Palaeolithic visual motifs and acoustic response. *The Journal of the Acoustical Society in America,**142*(3), 1332–1349. 10.1121/1.4998721 10.1121/1.4998721 28964077

[CR37] Fergulio, V., Bourdier, C., Delluc, M., Mora, P., Aujoulat, N., & Jaubert, J. (2019). Rock art, performance and Palaeolithic cognitive systems. The example of the Grand Panel palimpsest of Cussac Cave, Dordogne. *France. Journal of Anthropological Archaeology,**56*, 101–104. 10.1016/j.jaa.2019.101104

[CR38] Fortea, J., De La Rassila, M. & Rodríguez Otero, V. (2004). L’art pariétal et la séquence archéologique paléolithique de la grotte de Llonín (Peñamellera Alta, Asturias, Espagne). *Préhistoire, art et sociétés. Bulletin de la Société préhistorique de l´Ariège 59,*7–29.

[CR39] Frieman, C., & May, S. K. (2020). Navigating contact: Tradition and innovation in Australian contact rock art. *International Journal of Historical Archaeology,**24*, 342–366. 10.1007/s10761-019-00511-0

[CR40] Fritz, C., & Tosello, G. (2007). The hidden meaning of forms: Methods of recording Paleolithic parietal art. *Journal of Archaeological Method and Theory,**14*, 48–80. 10.1007/s10816-007-9027-3

[CR41] Garate, D., Rivero, O., Intxaurbe, I., & Díaz-González, L. M. (2021). Back to the wall: An approach to the reuse of symbolic underground spaces during the Late Upper Palaeolithic on the Bay of Biscay seaboard. *Bulletin De La Société Préhistorique Française,**17*, 173–194.

[CR42] García Guinea, M.A. & González Echegaray, J. (1966) Découvertes de nouvelles représentations d'art rupestre dans la grotte del Castillo. *Bulletin de la société de préhistoire Ariège-Pyrénées XXI :* 29–34.

[CR43] García Díez, M., Garrido, D., Hoffmann, D.L., Pettitt, P.B., Pike, A.L.W., & Zilhão, J. (2015). The chronology of hand stencils in European Paleolithic rock art: Implications of new U-series results from El Castillo Cave (Cantabria, Spain). *Journal of Anthropological Science **93:*1–18.10.4436/jass.9300410.4436/jass.93004 10.4436/JASS.93004 25615428

[CR44] Gay, M., Plassard, F., Müller, K., & Reiche, I. (2020). Relative chronology of Palaeolithic drawings of the Great Ceiling, Rouffignac cave, by chemical, stylistic and superimposition studies. *Journal of Archaeological Science: Reports,**29*, 102006. 10.1016/j.jasrep.2019.102006

[CR45] Gell, A. (1998). *Art and agency: An anthropological theory*. Oxford University Press.

[CR46] González Echegaray, J. (1964) Nuevos grabados y pinturas en las cuevas del Monte del Castillo. *Zephyrus* XV: 27–35.

[CR47] González Echegaray, J. (1972) Notas para el estudio cronológico del arte rupestre de la Cueva del Castillo. *Santander Symposium* (Santander 1970), pp.409–420.

[CR48] González Echegaray, J. & Moure Romanillo, J.A. (1970) Figuras rupestres inéditas en la Cueva del Castillo (Puente Viesgo, Santander). *Boletín del Seminario de Estudios de Arte y Arqueología*: XXXVI: 441–446.

[CR49] González García, R. (2001). *Art et espace dans les grottes paléolithiques cantabriques*. Jérôme Millon.

[CR50] González Sainz C. (2003). El conjunto parietal paleolítico de la galería inferior de la Garma (Cantabria), Avance de su organización interna. In: P. Bueno, & R. de Balbín Behrmann (Eds.), *El Arte prehistórico desde los inicios del siglo XXI, Primer symposium internacional del arte prehistórico de Ribadesella* (pp. 201–222). Ribadesella: Asociación Amigos de Ribadesella.

[CR51] González Sainz, C. (2007). Dating Magdalenian art in North Spain: The current situation. In P. Pettitt, P. Bahn, & S. Ripoll (Eds.), *Palaeolithic Cave Art at Creswell Crags in European Context* (pp. 247–261). Oxford University Press.

[CR52] González Sáinz, C. & Ruiz Redondo, A. (2010). La superposición entre figuras en el arte parietal Paleolítico. Cambios temporales en la región Cantábrica. *Cuadernos de Arqueología-Universidad de Navarra 18:* 41‐61. 10.15581/012.18.4474

[CR53] González Sainz, C., Cacho Toca, R. & Fukazawa, T. (2013) *Introduction to Palaeolithic cave paintings in northern Spain.* Tokyo: Texnai.

[CR54] Gormley, A. (2004). Art is a process. In C. Renfrew, C. Gosden, & E. DeMarrais (Eds.), *Substance, memory and display* (pp. 131–152). McDonald Institute for Archaeological Research.

[CR55] Groenen, M. (2008). La Imagen en el Arte de las Cuevas del Monte del Castillo. *Espacio, Tiempo Uy Forma,**1*, 105–112.

[CR56] Groenen, M. (2014). Présences humaines dans la grotte ornée d’El Castillo (Cantabrie, Espagne): dépôts et traces d’actions. In: P. Paillet (ed.) *Les arts de la préhistoire: micro-analyses, mises en context et conservation. Actes du Colloque Micro-Analyses et Datations de l’Art Préhistorique dans son Contexte Archéologique.* Paris: MADAPCA (16 – 18 November 2011).

[CR57] Groenen, M. & Groenen, M-C. (2017) La grotte ornée d’El Castillo (Cantabrie, Espagne) et l’espace. In: M. Otte (ed.) *Vocation préhistoire. Hommage à Jean-Marie Le Tensorer.* Liège: ERAUL.

[CR58] Groenen, M., & Groenen, M.-C. (2019). Modes of space appropriation in the decorated caves of El Castillo and La Pasiega (Puente Viesgo, Cantabria, Spain). *Journal of Archaeological Science: Reports,**28*, 1–13. 10.1016/j.jasrep.2019.102055

[CR59] Groenen, M. & Groenen, M-C. (in press) *La grotte ornée d’El Castillo. Un sanctuaire exceptionnel de la préhistoire*. Grenoble: Jéôme Millon.

[CR60] Groenen, M., Groenen, M.-C., Ceballos del Moral, J. M., & González Echegary, J. (2013). Review of seven years of research in the decorated cave of El Castillo (Cantabria, Spain). *Palethnologie,**5*, 49–50. 10.4000/palethnologie.2229

[CR61] Harman, J. (2008). Using decorrelation stretch to enhance rock art images. (accessed 23^rd^ September 2024). http://www.dstretch.com/AlgorithmDescription.html

[CR62] Harris, E., & Gunn, R. G. (2018). The use of Harris matrices in rock art research. In B. David & I. J. McNiven (Eds.), *The Oxford handbook of the archaeology and anthropology of rock art* (pp. 911–926). Oxford University Press.

[CR63] Hernando Álvarez, C. (2010). Estudio del arte parietal paleolítico desde la perspectiva arqueológica: Viejos fantasmas/nuevos enfoques. *El Futuro Del Pasado**1*:125–141.10.14201/fdp.24500

[CR64] Hodgson, D. (2008). The visual dynamics of Upper Palaeolithic cave art. *Cambridge Archaeological Journal,**18*(3), 341–353. 10.1017/S0959774308000401

[CR65] Hoffmann, D. L., Standish, C. D., Garcia-Diez, M., Pettitt, P. B., Milton, J. A., Zilhao, J., Alcolea-Gonzalez, J. J., Cantalejo-Duarte, P., Collado, H., De Balbín Behrmann, R., Lorblanchet, M., Ramos-Muñoz, J., Weniger, G.-C.H., & Pike, A. W. G. (2018). U-Th dating of carbonate crusts reveals Neandertal origin of Iberian cave art. *Science,**359*, 912–915. 10.1126/science.aap7778 29472483 10.1126/science.aap7778

[CR66] Ibero, Á., García-Diez, M., & Ochoa, B. (2024). The diachronic construction of Paleolithic cave art: Striated hind heads of the Cantabrian region. *Journal of Paleolithic Archeology,**7*, 1–24. 10.1007/s41982-024-00191-1

[CR67] Jaubert, J., Verheyden, S., Genty, D., Soulier, M., Cheng, H., Blamart, D., Burlet, C., Camus, H., Delaby, S., Deldicque, D., Edwards, R. L., Ferrier, C., Lacrampe-Cuyaubère, F., Lévêque, F., Maksud, F., Mora, P., Muth, X., Régnier, E., Rouzaud, J. N., & Santos, F. (2016). Early Neanderthal constructions deep in Bruniquel Cave in southwestern France. *Nature,**534*(7605), 111–114. 10.1038/nature18291 27251286 10.1038/nature18291

[CR68] Keyser, J. D., & Whitley, D. S. (2006). Sympathetic magic in western North American rock art. *American Antiquity,**71*(1), 3–26. 10.2307/40035319

[CR69] Klassen, M.A. (2005) Áísínai’pi (Writing-on-Stone) in traditional, anthropological, and popular thought. In: Loendorf, L.L., Chippindale, C. and Whitley, D.S. (Eds).*Discovering North American rock art.* Tucson: University of Arizona Press.

[CR70] Laming-Emperaire, A. (1962). *La signifcation de l’Art rupestre Paléolithique*. A. & J. Picard, Paris.

[CR71] Langley, M. C., & Taçon, P. S. C. (2010). The age of Australian rock art: A review. *Australian Archaeology,**71*(1), 70–73. 10.1080/03122417.2010.11689386

[CR72] Leroi-Gourhan, A. (1965). *Préhistoire de l’art occidental*. Mazenod.

[CR73] Leroi-Gourhan, A. (1982). *The dawn of European art. An introduction to Palaeolithic cave painting*. Cambridge University Press.

[CR74] Lorblanchet, M. (1994). *Cougnac. International Newsletter on Rock Art,**7*, 6–7.

[CR75] Lorblanchet, M. (2010). *Art pariétal: grottes ornées du Quercy.* Rodez: Éditions de Rouergue.

[CR76] Maillo-Fernández, J., Marín, J., Martín Perea, D., Garralda, M., Abellán Beltrán, N., Solano-Megías, I., González Molina, I., Herrero, D., Sistiaga, A., Álvarez-Vena, A., Asiain, R., Moral-Rodríguez, M., Rodríguez-Robredo, D., Luzón-Ruiz, S., Gómez-Fernández, A., Neira, A. & Bernaldo de Quirós, F. (2023) Ocupaciones humanas en la cueva de El Castillo (Puente Viesgo, Cantabria). In S.D. Domínguez-Solera (Ed.) *Cuando empezábamos a ser nosotr@s: Curso sobre el Paleolítico Inferior y Medio a nivel mundial*&nbsp; (Vol. IV. pp.85–106).

[CR77] Martinón-Torres, M., Garate, D., Herries, A. I. R., & Petraglia, M. D. (2024). No scientific evidence that *Homo naledi* buried their dead and produced rock art. *Journal of Human Evolution,**195*, 103464. 10.1016/j.jhevol.2023.103464 37953122 10.1016/j.jhevol.2023.103464

[CR78] Marquet, J. C., Freiesleben, T. H., Thomsen, K. J., Murray, A. S., Calligaro, M., Macaire, J. J., Robert, E., Lorblanchet, M., Aubry, T., Bayle, G., Bréhéret, J. G., Camus, H., Chareille, P., Egels, Y., Gullard, E., Guérin, G., Gautret, P., Liard, M., O’Farrell, & M., Jaubert, J. (2023). The earliest unambiguous Neanderthal engravings on cave walls: La Roche-Cotard, Loire Valley. *France. PLOS One,**18*(6), e0286568. 10.1371/journal.pone.0286568 37343032 10.1371/journal.pone.0286568 PMC10284424

[CR79] Medina-Alcaide, M.-A., Garate, D., Intxaurbe, I., Sanchidrián, J. L., Rivero, O., Ferrier, C., Mesa, M. D., Pereña, J., & Libano, I. (2021). The conquest of the dark spaces: An experimental approach to lighting systems in Paleolithic caves. *PLoS ONE,**16*(6), e0250497. 10.1371/journal.pone.0250497 34133423 10.1371/journal.pone.0250497 PMC8208548

[CR80] Merion Jones, A. (2017). Rock art and ontology. *Annual Review of Anthropology,**46*, 167–181. 10.1146/annurev-anthro-102116-041354

[CR81] Moro Abadía, O., & Porr, M. (Eds.). (2022). *Ontologies of rock art: Images, relational approaches, and indigenous knowledges*. London: Routledge.

[CR82] Morphy, H. (1991). *Ancestral connections: Art and an aboriginal system of knowledge*. University of Chicago Press.

[CR83] Morphy, H. (1999). Encoding the Dreaming -A theoretical framework for the analysis of representational processes in Australian Aboriginal art. *Australian Archaeology,**49*, 13–22. 10.1080/03122417.1999.11681648

[CR84] Morphy, H. (2003). *Aboriginal Art*. Phaidon.

[CR85] Moure, A., González Sainz, C., Bernaldo de Quirós, F., & Cabrera Valdés, V. (1996). Dataciones absolutas de pigmentos en cuevas cantábricas: Altamira, El Castillo, Chimeneas y Las Monedas. In A. Moure (Ed.), *El Hombre fósil 80 años después* (pp. 295–324). Universidad de Cantabria. Santander.

[CR86] Moyes, H. (2012). *Sacred darkness: A global perspective on the ritual use of caves*. University Press of Colorado.

[CR87] O’Connor, S., Barham, A., & Woolagoodja, D. (2008). Painting and repainting in the west Kimberley. *Australian Aboriginal Studies,**2008*(1), 22–38.

[CR88] Ochoa, B. (2017).*Graphic space, visibility and cave transit. The use of caves with Palaeolithic art in the Cantabrian Region*. Oxford: BAR Publishing.

[CR89] Ochoa, B., García-Diez, M., Maíllo-Fernández, J.-M., Arrizabalaga, A., & Pettitt, P. (2019). Gravettian figurative art in the Western pyrenees: Stratigraphy, cultural context and chronology. *European Journal of Archaeology,**22*(2), 168–184. 10.1017/eaa.2018.31

[CR90] Ontañón, R. & Teira, L. (2016). Arte esquemático en la cueva de El Castillo (Puente Viesgo). In: M. L. Serna Gancedo, A. Martínez Velasco & V. Fernández Acebo (eds.), *Después de Altamira: arte y grafismo rupestre post-paleolítico en Cantabria* (pp.233–246). Santander. ACANTO-ACDPS-Gobierno de Cantabria.

[CR91] Ortega, A. I., García-Díez, M., & Martín Merino, M. A. (2020). Palaeolithic creation and later visits of symbolic spaces: Radiocarbon AMS dating and cave art in the Sala de las Pinturas in Ojo Guareña (Burgos, Spain). *Archaeological and Anthropological Sciences,**12*(240), 1–15. 10.1007/s12520-020-01208-w

[CR92] Ortega-Martínez, P., & Ruiz-Redondo, A. (2018). An approach for understanding site location preferences on Pas River Basin during Late Magdalenian. Landscape analysis of Las Monedas cave. *Journal of Archaeological Science: Reports,**19*, 804–810. 10.1016/j.jasrep.2017.08.003

[CR93] Palacio-Pérez, E. (2024). The UNESCO World Heritage list in a globalized world: The case of the Paleolithic caves of northern Spain (1985–2008). In: O. Moro Abadía, M.W. Conkey, M.W. & McDonald J. (Eds.) *Deep-time images in the age of globalization. Rock art in the 21st century* (pp. 207–217)*.* Cham: Springer Nature.

[CR94] Pastoors, A., & Weniger, G.C. (2011). Cave art in context: Methods for the analysis of the spatial organization of cave sites. *Journal of Archaeological Research,**19*, 377–400. 10.1007/s10814-011-9050-5

[CR95] Pearce, D. (2008). A comment on Swart’s rock art sequences and use of the Harris Matrix in the Drakensberg. *Southern African Humanities,**18*(2), 173–188.

[CR96] Pettitt, P. (2016). Darkness visible. Shadows, art and the ritual experience of caves in Upper Palaeolithic Europe. In: M. Dowd & R. Hensey. (Eds) *The Archaeology of Darkness* (pp. 11–23). Oxford: Oxbow Books.

[CR97] Pettitt, P. (2002). The Neanderthal dead: Exploring mortuary variability in Middle Palaeolithic Eurasia. *Before Farming,**4*, 1–26. 10.3828/bfarm.2002.1.4

[CR98] Pettitt, P. (2022) Did*Homo naledi*dispose of their dead in the Rising Star cave system?*South African Journal of Science*1*18(11/12):*1–3.10.17159/sajs.2022/15140

[CR99] Pike, A. W. G., Hoffmann, D. L., García-Diez, M., Pettitt, P. B., Alcolea, J., De Balbín, R., González-Sainz, C., De Las Heras, C., Lasheras, J. A., Montes, R., & Zilhão, J. (2012). U-series dating of Paleolithic art in 11 caves in Spain. *Science,**336*(6087), 1409–1413. 22700921 10.1126/science.1219957

[CR100] Porr, M. (2018). Country and relational ontology in the Kimberley, Northwest Australia: Implications for understanding and representing archaeological evidence. *Cambridge Archaeological Journal,**28*(3), 395–409. 10.1017/S0959774318000185

[CR101] Prijatelj, A., Skeates, R. (2018). Caves as vibrant places: A theoretical manifesto. In: L. Büster, E., spsampsps Warmenbol D., Miekuz (Eds.) *Between worlds: Understanding ritual cave use in late prehistory.* Cham: Springer. 10.1007/978-3-319-99022-4_2

[CR102] Randolph-Quinney, P.S. (2015). A new star rising: Biology and mortuary behavior of *Homo naledi. South African Journal of Science**111*(9/10):1–4. 10.17159/SAJS.2015/A0122

[CR103] Rifkin, R. F. (2012). Processing ochre in the Middle Stone Age: Testing the inference of prehistoric behaviours from actualistically derived experimental data. *Journal of Anthropological Archaeology,**31*(2), 174–195. 10.1016/j.jaa.2011.11.004

[CR104] Ripoll, E. (1956). Nota acerca de algunas nuevas figuras rupestres de las cuevas de El Castillo y La Pasiega (Puente Viesgo, Santander). *Actas del IV Congreso Internacional de Ciencias Prehistóricas y Protohistóricas (Madrid 1954)* (pp. 301–310). Zaragoza.

[CR105] Ripoll, E. (1972). Un palimpsesto rupestre de la Cueva del Castillo. *Santander Symposium (Santander 1970)* (pp.457–464). Santander.

[CR106] Ripoll-López, S., Bayarri Cayón, V., Castillo López, E., Latova-Fernández Luna, J., & Muñoz Ibáñez, F. J. (2020). A chronological proposal for El Castillo Cave (Puente Viesgo, Cantabria) based on its iconographic stratigraphy. *Boletín del Seminario de Estudios de Arte y Arqueología*, *85/86:* 149–176.10.24197/ba.0.2020.149-176

[CR107] Rivero, O., Garate, D., Salazar, S., & Intxaurbe, I. (2019). The Cantabrian Lower Magdalenian striated hinds on scapulae: Towards a new definition of a graphic morphotype. *Quaternary International,**506*, 69–79. 10.1016/j.quaint.2019.01.037

[CR108] Ruiz Redondo, A. (2012). Una nueva revisión del Panel de las Manos de la cueva de El Castillo (Puente Viesgo, Cantabria). *Munibe Antropologia-Arkeologia,**61*, 17–27.

[CR109] Santos, A. T., Barbosa, A. F., Luís, L., Silvestre, M., & Aubry, T. (2021). Dating the Côa Valley rock art 25 years later: An archaeological and geoarchaeological approach. In T. Aubry, A. T. Santos, & A. Martins (Eds.), *Côa Symposium: Novos olhares sobre a Arte Paleolítica* (pp. 94–127). Associação dos Arqueólogos Portugueses e Fundação Côa-Parque.

[CR110] Sapwell, M. (2017). Understanding palimpsest rock art with the art as agency approach: Gell, Morphy, and Laxön, Nämforsen. *Journal of Archaeological Method and Theory,**24*, 352–376. 10.1007/s10816-015-9270-y

[CR111] Sauvet; G., Bourrillon, R., Conkey, M., Fritz, C., Gárate-Maidagan, D., Rivero Vilá, O., Tosello, G. & White, R. (2017) Uranium-thorium dating method and Palaeolithic rock art. *Quaternary International 432 (B):* 86 – 92. 10.1016/j.quaint.2015.03.053

[CR112] Schaafsma, P. (2014). *Images and power: Rock art and ethics*. Springer.

[CR113] Schaafsma, P. & Tsosie, W. B. (2009). Xeroxed on stone: Times of origin and the Navajo holy people in canyon landscapes. In: J.J. Christie (Ed.)*Landscapes of origin in the Americas, edited by Jessica Joyce Christie*(pp. 15–31). Tuscaloosa: University of Alabama Press.

[CR114] Swart, J. (2004). Rock art sequences in uKhahlamba-Drakensberg Park, South Africa. *Southern African Humanities,**16*, 13–35.

[CR115] Till, R. (2014). Sound archaeology: Terminology, Palaeolithic cave art and the soundscape. *World Archaeology,**46*(3), 292–304. 10.1080/00438243.2014.909106

[CR116] Valladas, H., Quiles, A., Delque-Kolic, M., Kaltnecker, E., Moreau, C., Pons-Branchu, E., Vanrell, L., Olive, M., & Delestre, X. (2017). Radiocarbon dating of the decorated Cosquer cave (France). *Radiocarbon,**59*(2), 621–633. 10.1017/RDC.2016.87

[CR117] Valladas, H., Tisnérat-Laborde, N., Cachier, H., Arnold, M., Bernaldo De Quirós, F., Cabrera-Valdés, V., Clottes, J., Courtin, J., Fortea-Pérez, J., Gonzáles Sainz, C., & Moure-Romanillo, A. (2001). Radiocarbon AMS dates for Paleolithic cave paintings. *Radiocarbon,**43*(2B), 977–986. 10.1017/S0033822200041643

[CR118] Velliky, E. C., Porr, M., & Conard, N. J. (2018). Ochre and pigment use at Hohle Fels cave: Results of the first systematic review of ochre and ochre-related artefacts from the Upper Palaeolithic in Germany. *PLoS ONE,**13*(2), e0209874. 10.1371/journal.pone.0209874 30589914 10.1371/journal.pone.0209874 PMC6307870

[CR119] Warzée, N., Groenen, M., Rosoux, J., Debeir, O., Ercek, R. and Reichling, C. (2007) Numérisation 3D de la grotte d’El Castillo. *Archéologie et réalité virtuelle, Colloque Virtual Retrospect, Bordeaux&nbsp; november 2007, 14–16.*

[CR120] White, R., Bosinski, G., Bourrillon, R., Clottes, J., Conkey, M. W., Corchón Rodriguez, S., Cortés-Sánchez, M., de la Rasilla Vives, M., Delluc, B., Delluc, G., Fergulio, V., Floss, H., Foucher, P., Fritz, C., Fuentes, O., Garate, D., González Gómez, J., González-Morales, M. R., González-Pumariega Solis, M., & Willis, M.D. (2020). Still no archaeological evidence that Neanderthals created Iberian cave art. *Journal of Human Evolution,**144*, Article 102640. 10.1016/j.jhevol.2019.102640

[CR121] Wisher, I., & Needham, A. (2023). Illuminating palaeolithic art using virtual reality: A new method for integrating dynamic firelight into interpretations of art production and use. *Journal of Archaeological Science: Reports,**50*, Article 104102. 10.1016/j.jasrep.2023.104102

[CR122] Wisher, I., Pettitt, P., Kentridge, R. (2023). The deep past in the virtual present: Developing an interdisciplinary approach towards understanding the psychological foundations of palaeolithic cave art. *Nature: Scientific Reports 13:* 19009. 10.1038/s41598-023-46320-8 10.1038/s41598-023-46320-8 PMC10624876 37923922

[CR123] Wisher, I., Pettitt, P., & Kentridge, R. (2024). Conversations with caves: The role of pareidolia in the Upper Palaeolithic figurative art of Las Monedas and La Pasiega (Cantabria, Spain). *Cambridge Archaeological Journal,**34*(2), 315–338. 10.1017/S0959774323000288

