# Supplementary material for: Dialogues Across Time? Conceptualising the Temporal Relationships of Palimpsests in the Upper Palaeolithic Cave Art of El Castillo (Cantabria, Spain)
Source: J Archaeol Method Theory. 2025 Jun 12;32(3):49. doi: 10.1007/s10816-025-09717-5 (PMC12162723; doi:10.1007/s10816-025-09717-5)
Supplement: Supplementary file 1 — (PDF 102 MB) [file 10816_2025_9717_MOESM1_ESM.pdf]

Dialogues Across Time? Conceptualising the Temporal Relationships of Palimpsests in the Upper Palaeolithic Cave Art of El Castillo (Cantabria, Spain)

Izzy Wisher[1,2] and Eduardo Palacio-Pérez[3]

*Corresponding author. Email: izzywisher@cas.au.dk

[1] Department of Archaeology and Heritage Studies, Aarhus University, Denmark.

[2] Department of Linguistics, Cognitive Science and Semiotics, Aarhus University, Denmark.

[3] Center of Prehistoric Caves, Government of Cantabria, Spain.

Supplementary Information

The supplementary information here details the assessments of superimpositions made to support the manuscript and includes additional photographic documentation to support these assessments.

1. The Polychrome Panel

Observations made during fieldwork, close-range macro-photos and DStretch imaging were used to clarify the superimpositions of the Polychrome Panel. Here, we provide details of each superimposition and how these were utilised to produce the Harris Matrix for the panel.

1.1. Undefined Phase

There were several depictions on the *Polychrome Panel* that do not have a clear stratigraphic order, due to a lack of superimposition between them and other depictions. Generally, these depictions appear to be produced with the same hue of pigment, a red-orange ochre, which may tentatively suggest they were produced using the same material. These depictions are also constrained to a different spatial position on the panel, placed high to the upper-right where presumably the artist climbed on an adjacent rockfall to produce the depictions (Figure S1). These two factors suggest, albeit are not enough to confirm, these depictions may correspond to the same phase of artistic activity.

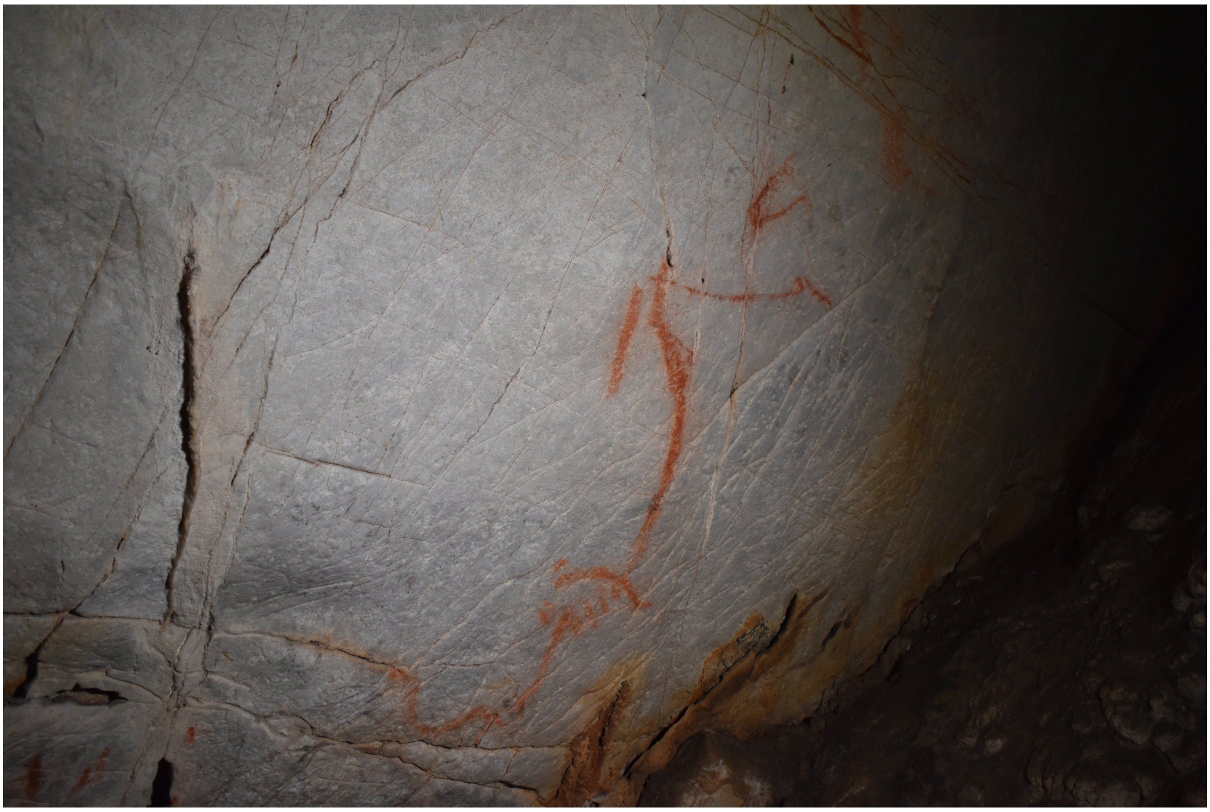

Figure S1. Depictions EC.2.17, EC2.18. EC2.19 and the rear leg of EC2.20. Note the similarity in colour hue, close spatial positioning and the rockfall in front of the panel which likely aided in the production of these depictions.

1.2. Superimpositions: Phase 1

The first phase of artistic activity that was defined for this panel is characterised by a series of red ochre hand-stencils and one small non-figurative sign, that all consistently underlie the figurative depictions on the panel. This phase was established by evaluating the stratigraphic order of these depictions in relation to other depictions on the panel, where a natural grouping of these depictions as the earliest defined phase on the panel emerged.

Depictions EC2.8, EC2.9 and EC2.10 are hand-stencils that underlie the large black bison, EC2.3 (Figure S2; Figure S3). Similarly, the non-figurative sign EC2.14 and the hand-stencil EC2.15 underlie the black bison depiction EC2.13 (Figure S4). This clearly indicates the red hand-stencils and non-figurative sign consistently predate the black bison depictions on the panel. The consistency in theme (hand-stencils/non-figurative motifs), the use of red ochre, and the stratigraphic order of these depictions under the black depictions on the panel all suggest these depictions should be grouped as one phase.

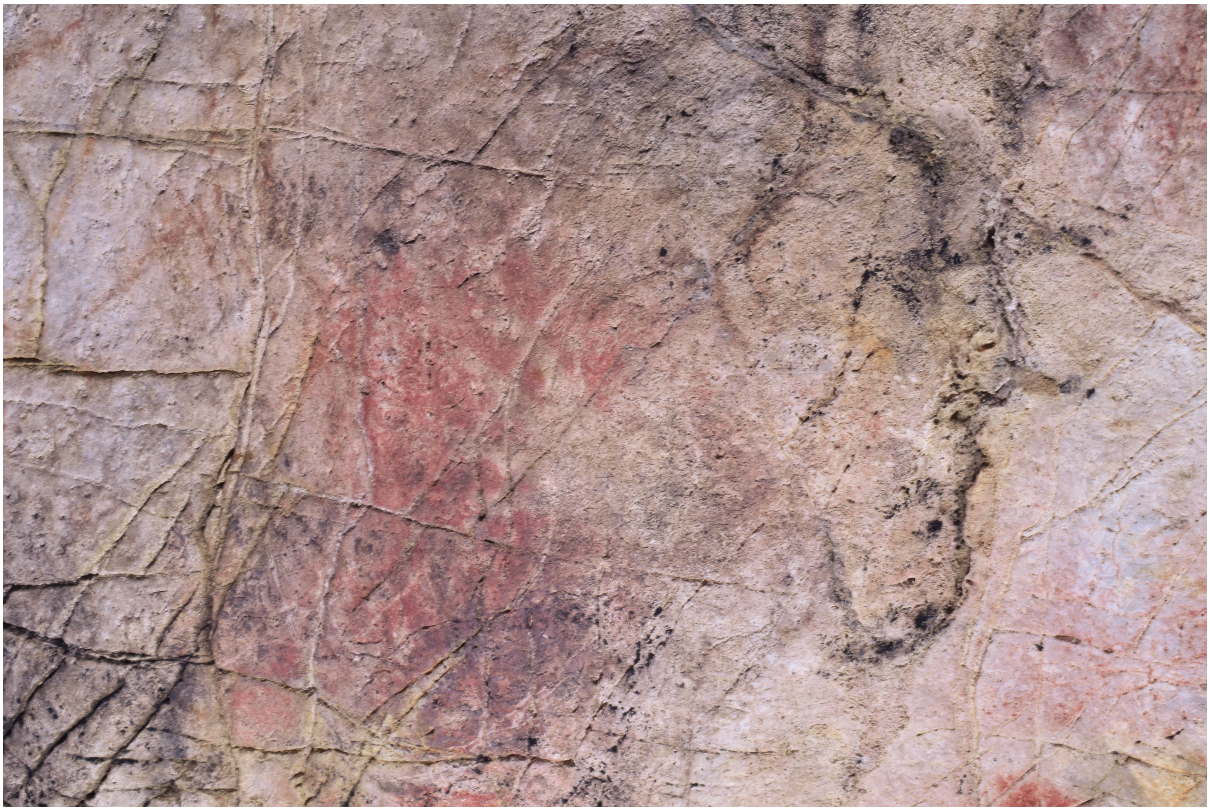

Figure S2. Hand-stencils EC2.8 and EC2.9 underlying the black charcoal infill of depiction EC2.3.

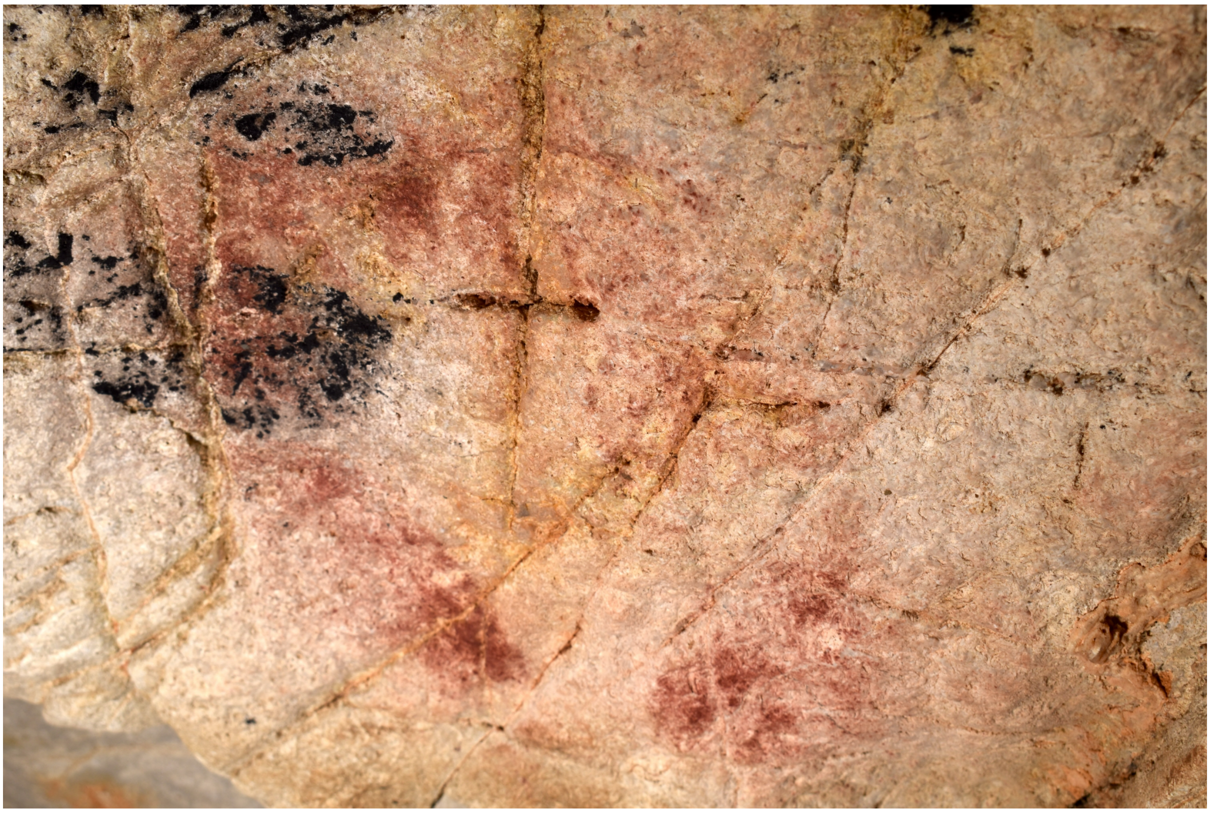

Figure S3. Hand-stencil depiction EC2.10 underlying the hoof of the rear leg of EC2.3.

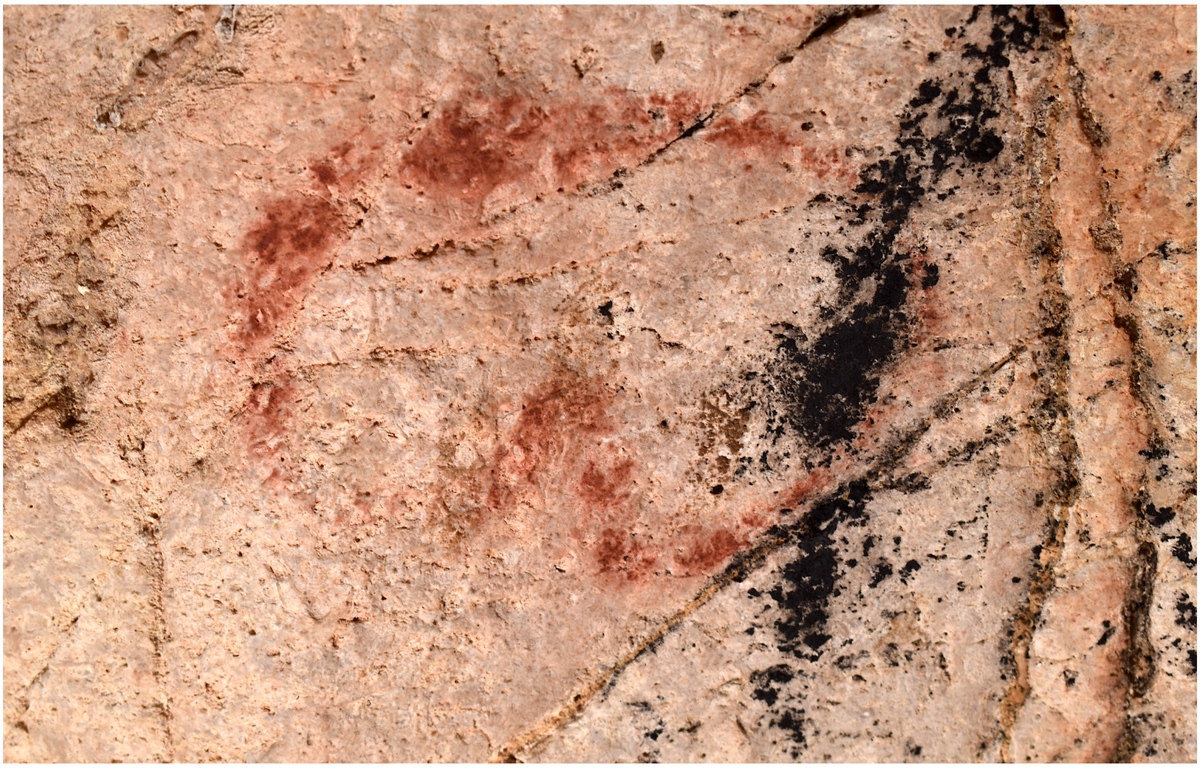

Figure S4. Macro-photo of depiction EC2.14, underlying the dorsal line of EC2.13.

The second indication of the stratigraphic order of this group of depictions derives, albeit more tentatively, from a superimposition of a hind depiction (EC2.6) over the hand-stencil depictions EC2.9. This is difficult to visually distinguish, but through the use of DStretch and as has been suggested by previous evaluations of the chronology of this panel, the hand-stencil appears to underlie the ventral line and legs of the hind depiction (Figure S5). This further places this group of depictions as possibly predating the hind depictions, although given that this is based primarily on one superimposition, it must be noted that this evaluation is tentative.

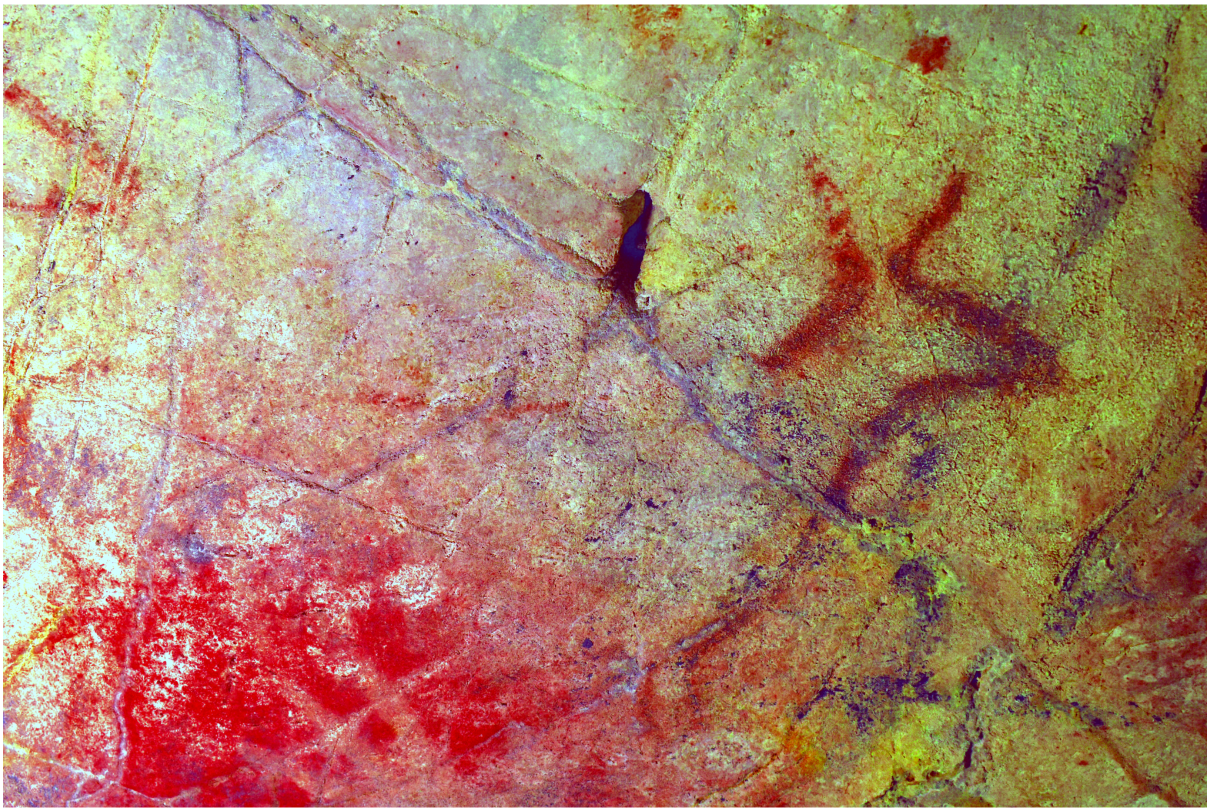

Figure S5. DStretch image of depiction EC2.6. Although difficult to distinguish, the front ventral line and front legs of hind depiction EC2.6 appear to overlie the hand-stencil EC2.9.

1.3. Superimpositions: Phase 2

The superimposition of the hind depiction EC2.6 thus defines this depiction as belonging to another stratigraphic phase in the palimpsest. The other hind depiction EC2.4 is grouped as belonging to this phase too, due to the close similarity in the style and technique used to produce both of these hind depictions; in all likelihood, these depictions were produced at the same time, possibly even by the same artist. Depiction EC2.6 shows some evidence of being refreshed at a latter date, and additionally clearly underlies depiction EC2.3 (Figure S6). The refreshing of the depiction in black can be visually distinguished, and appears to correspond to the final phase of graphic activity of the panel which consists of a series of black depictions.

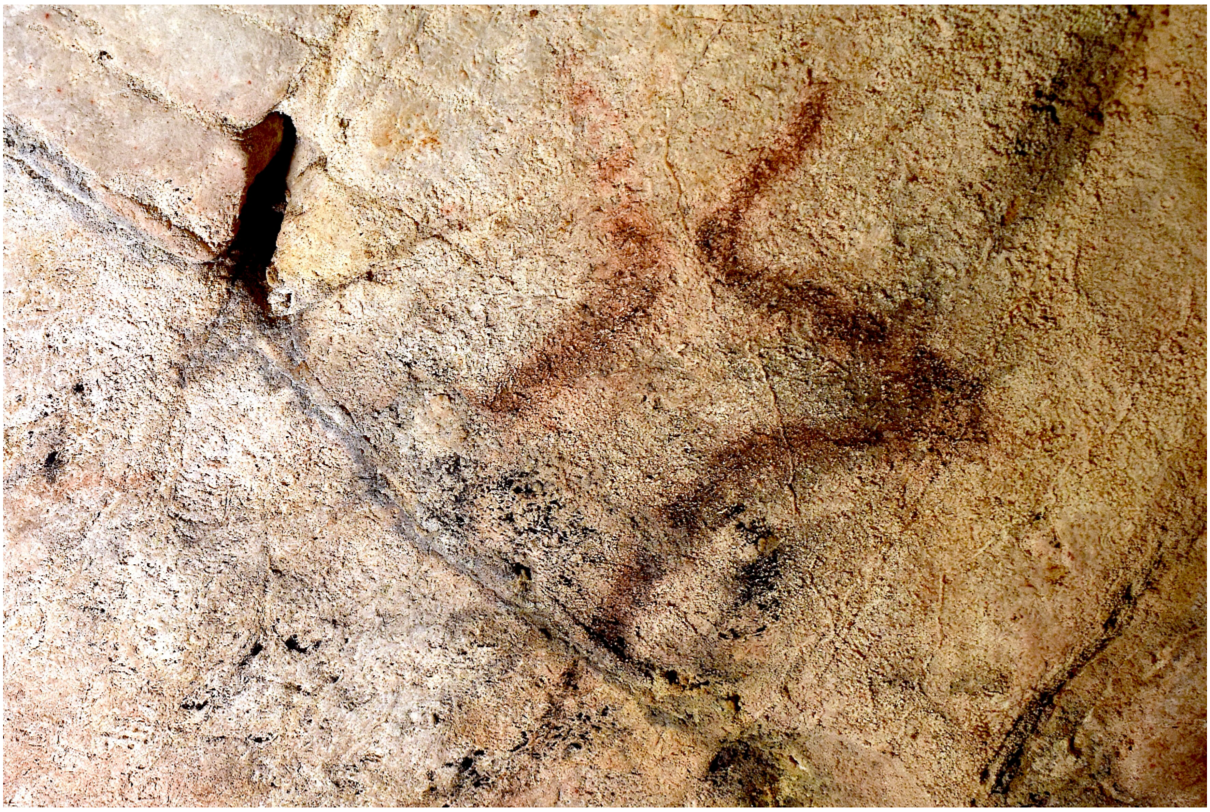

Figure S6. Head of depiction EC2.6, which shows the underlying original red outline and the retraced outline in black.

1.4. Superimpositions: Phase 3

The final phase of this panel appears to be a series of depictions representing bison, produced in black charcoal with some engraving to enhance the outline of the depictions. Although AMS radiocarbon dating conducted on these depictions nearly 25 years ago (Valladas et al. 2001) provides differing ages for these bison, it is possible that there has been some recent contamination - particularly for the bison depiction on a small boulder feature (EC2.16) - that resulted in young ages being obtained for some of these depictions. This is further suggested by there being several samples taken for AMS radiocarbon dating from the same depiction by Valladas et al. (2001) that are not internally consistent in age (i.e., different samples from the same depiction do not overlap in age: Table S1).

Table S1. AMS radiocarbon dates for the four charcoal bison depictions on the Polychrome Panel. Note the high variability in dates from the same depiction, and that several dates do not statistically overlap despite being from the same depiction.

Depiction	Uncal. date	Calibrated date	Reference
Bison 18a (EC2.3)	12,629 ± 110	15,332 - 14,352	Valladas et al., 2001
	13,060 ± 200	16,237 - 15,098	Valladas et al., 2001
	13,520 ± 130	16,721 - 15,898	Valladas et al., 2001

Bison 18b (EC2.13)	12,910 ± 180	16,020 - 14,827	Valladas et al., 2001
Bison 18c (EC2.16)	10,510 ± 100	12,690 - 12,080	Valladas et al., 2001
	11,270 ± 80	13,296 - 12,996	Valladas et al., 2001
	10,720 ± 100	12,790 - 12,518	Valladas et al., 2001
	10,740 ± 100	12,895 - 12,592	Valladas et al., 2001
	12,390 ± 190	15,220 - 14,005	Valladas et al., 2001
Bison 19 (EC2.2)	13,530 ± 120	16,695 - 15,920	Valladas et al., 2001
	13,570 ± 130	16,810 - 15,981	Valladas et al., 2001
	13,710 ± 140	17,016 - 16,163	Valladas et al., 2001
	14,090 ± 150	17,557 - 16,635	Valladas et al., 2001
	13,510 ± 190	16,926 - 15,769	Valladas et al., 2001

Given these issues in the AMS radiocarbon dates, and the similarity in technique used across three of the bison depictions (EC2.3, EC2.13, and EC2.16) where short vertical lines are used to represent the hair on the dorsal line of the bison and engraving is used after the application of charcoal to enhance features of the depiction, these depictions are considered as belonging to the same phase (Figure S7; Figure S8). It is possible that this "phase" represents several iterations of graphic activity across a number of years but, as with other phases on this panel and the *Ceiling of the Hands,* a more coarse-scale, conservative approach is taken here to distinguishing different phases of superimposition. As discussed in the previous sections, where these black depictions are in a stratigraphic relationship with other depictions, they consistently overlie these depictions indicating that this is the youngest phase in the panel.

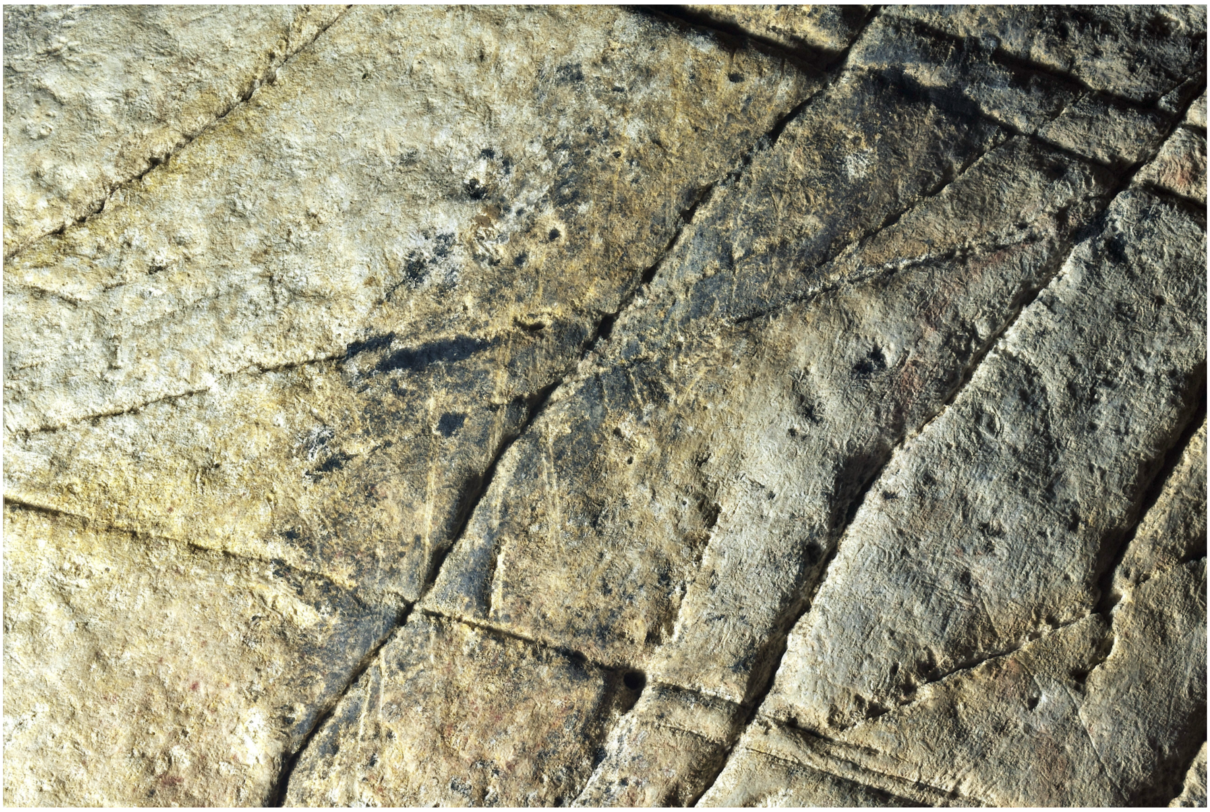

Figure S7. Vertical engraved lines on the dorsal line of depiction EC2.3, to add details of hair to the depiction.

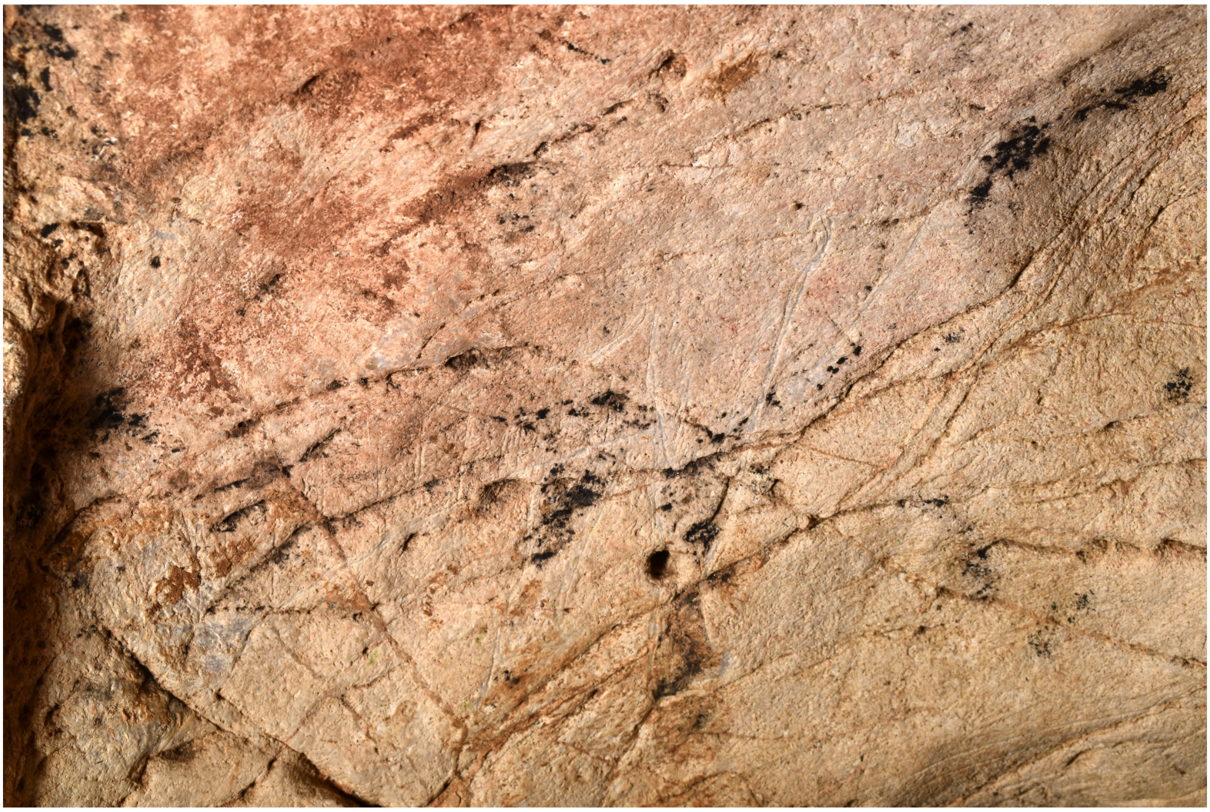

Figure S8. Engraved features used to add details and enhance the charcoal outline on the front legs and chest of depiction EC2.16.

2. The Ceiling of the Hands

As with the *Polychrome Panel*, observations made during fieldwork, macro-photos and DStretch imaging were used to clarify the superimpositions. The full extent of the ceiling was not assessed, with the focus of the study instead being on the areas where there are superimpositions between depictions. As a result, fewer depictions (e.g. hand-stencils) were recorded in this study than has been previously documented for this panel. Given that the focus of this research was not a comprehensive documentation of the depictions on the panel, but rather characterising the relationships of the superimpositions, it was felt that a comprehensive analysis was not strictly required, particularly as the depictions on this panel have been thoroughly analysed and documented by previous studies (Ripoll-López et al. 2020). Further, it is likely that this focus on superimpositions has resulted in fewer phases being accounted for in our study than previously suggested. Again, given that this research concerns the superimpositions, rather than establishing the chronological phases of the panel *per se,* it was felt that a more comprehensive analysis of the chronology was not required in order to evaluate the relationships between superimposed depictions.

The *Ceiling of the Hands* has a more complex palimpsest than the *Polychrome Panel,* with many different superimpositions between depictions that needed to be carefully considered and resolved to distinguish different phases to the graphic activity. A conservative approach was taken here, where depictions that were produced using the same coloured pigment were grouped into the same phase unless they showed a stratigraphic relationship that necessitated them being placed in a separate phase. Only stratigraphic relationships between superimposed depictions were used to distinguish different phases, to ensure that a conservative approach was taken to assessing the number of possible phases in this panel.

2.1. Superimpositions: Phase 1

The oldest phase on the *Ceiling of the Hands* appears to be a series of blown discs and hand-stencils (EC1.29, EC1.54, EC1.55, EC1.56, EC1.59, EC1.62, EC1.63, EC1.68, EC1.69, EC1.71) produced in a purple-red hued ochre pigment. While overlying calcite may affect the perception of the colour of depictions, there were several depictions that had minimal overlying calcite and still exhibited the distinctive purple-red hue, which appears to be distinct from the other red hued ochre depictions on the panel (Figure S9; Figure S10; Figure S11; Figure S12).

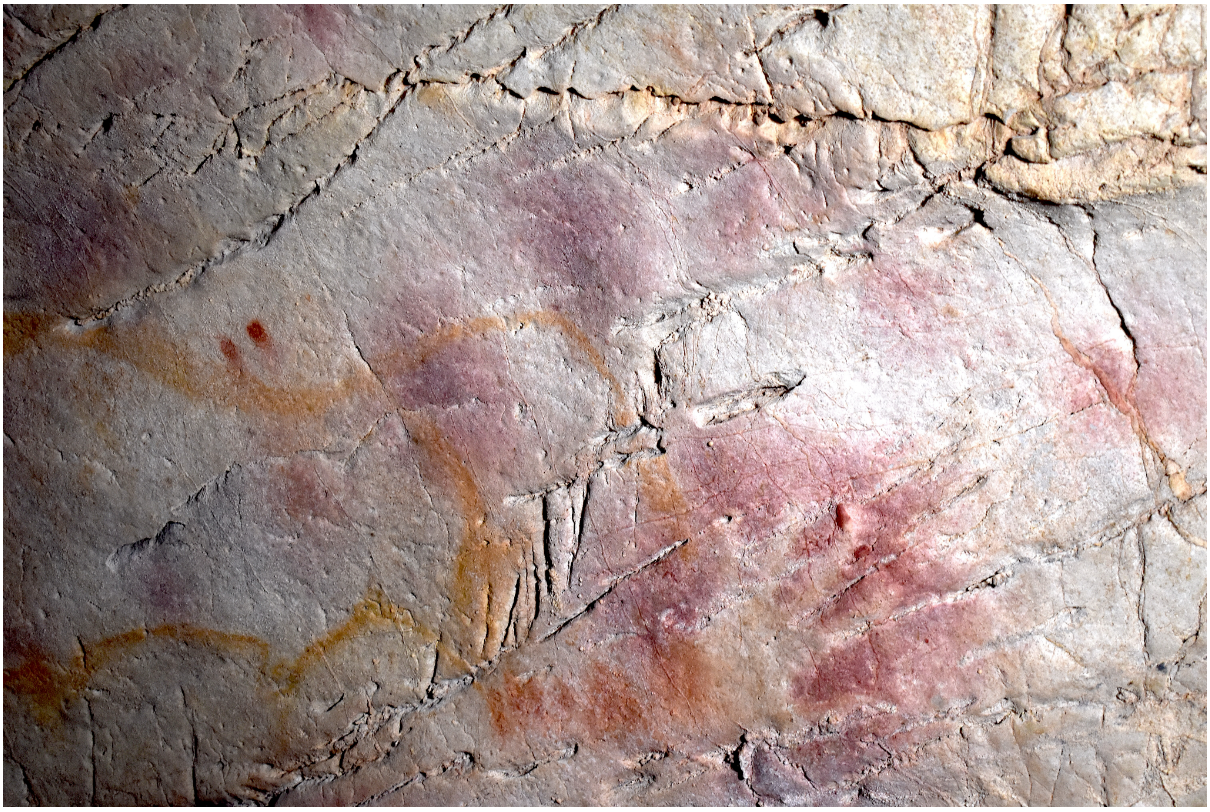

Figure S9. An example of the blown discs (EC1.56) and a hand-stencil (EC1.59) that exhibit the purple-red hued ochre pigment. Note that there appears to be no calcite overlying these depictions, and the colour is clearly distinct from the yellow-hued and more vibrant red-hued ochre also visible in the image.

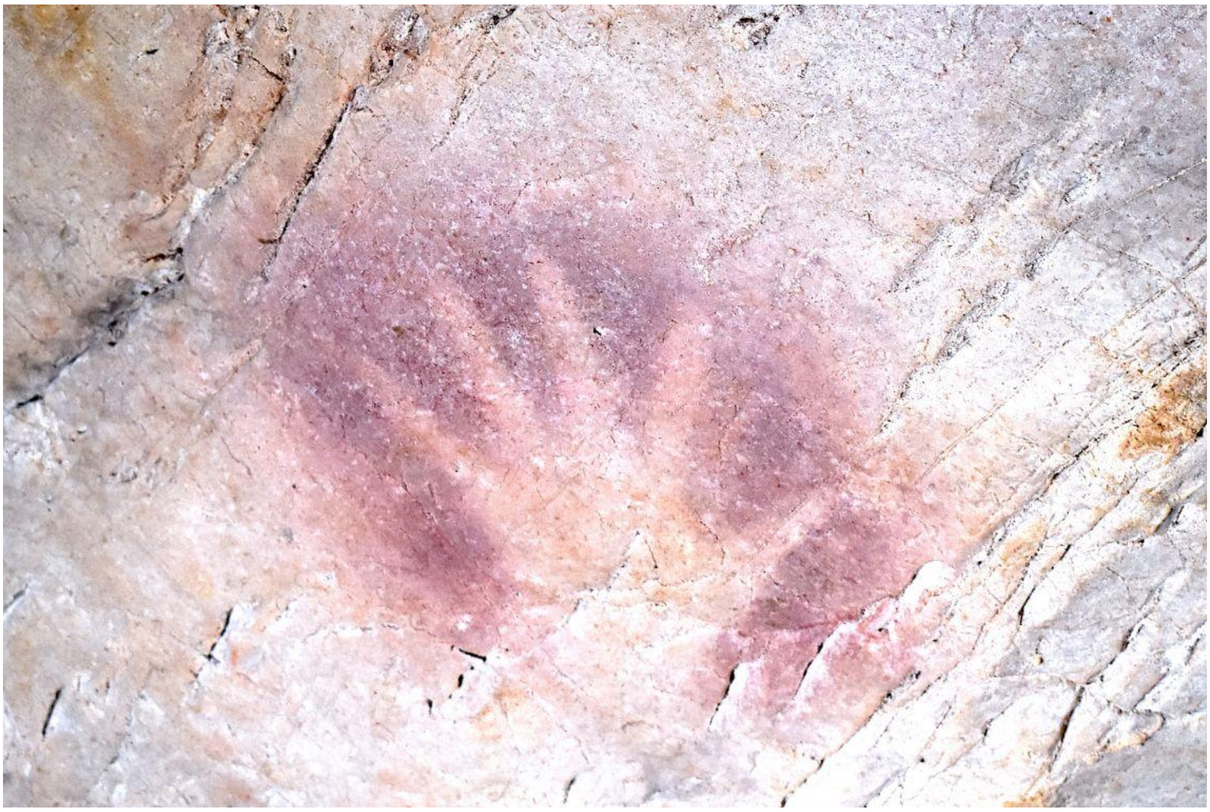

Figure S10. Depiction EC1.62 that exhibits the purple-red hued ochre pigment and similarly has minimal to no calcite formation that may distort the perception of the pigment hue.

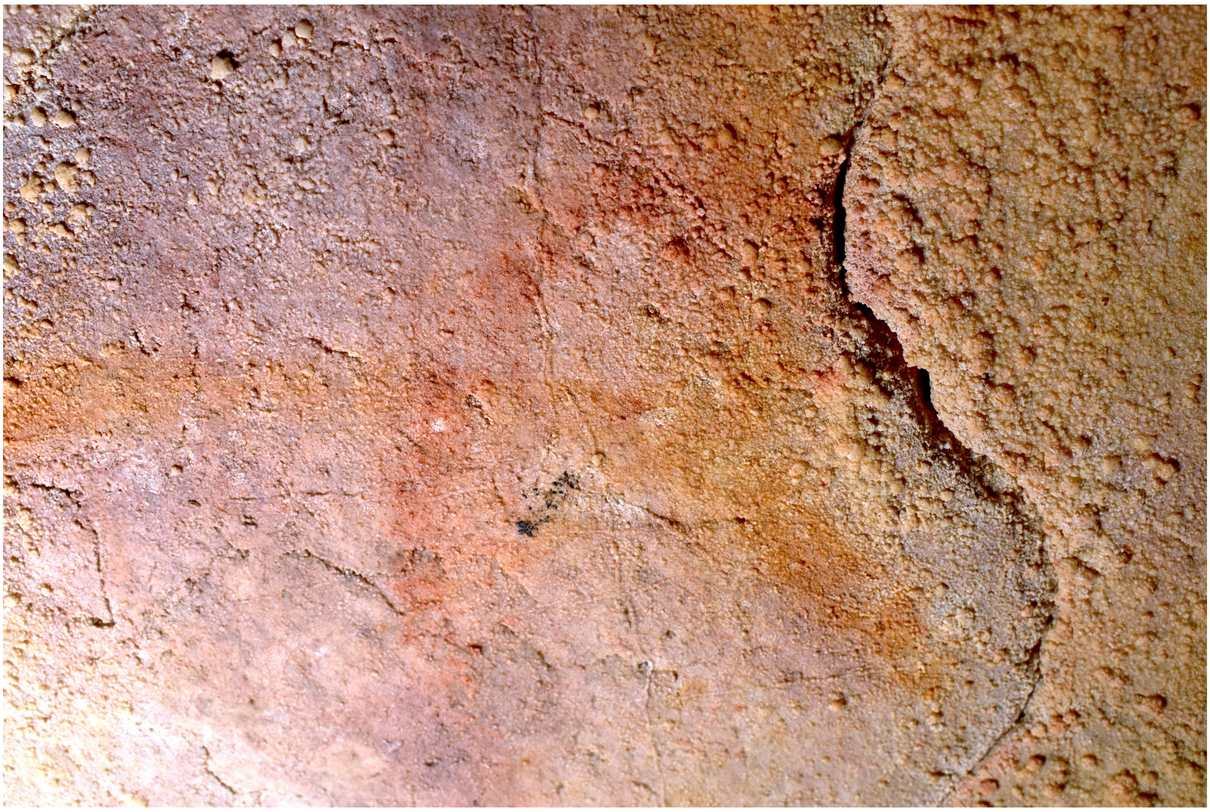

Figure S11. Macro-photo of superimposed lines corresponding to the front of EC1.57 (the curved yellow line represents the horn of this depiction) as it intersects with a blown purple-red disc. A vibrant red line can also be observed superimposed over the yellow and purple-red pigments.

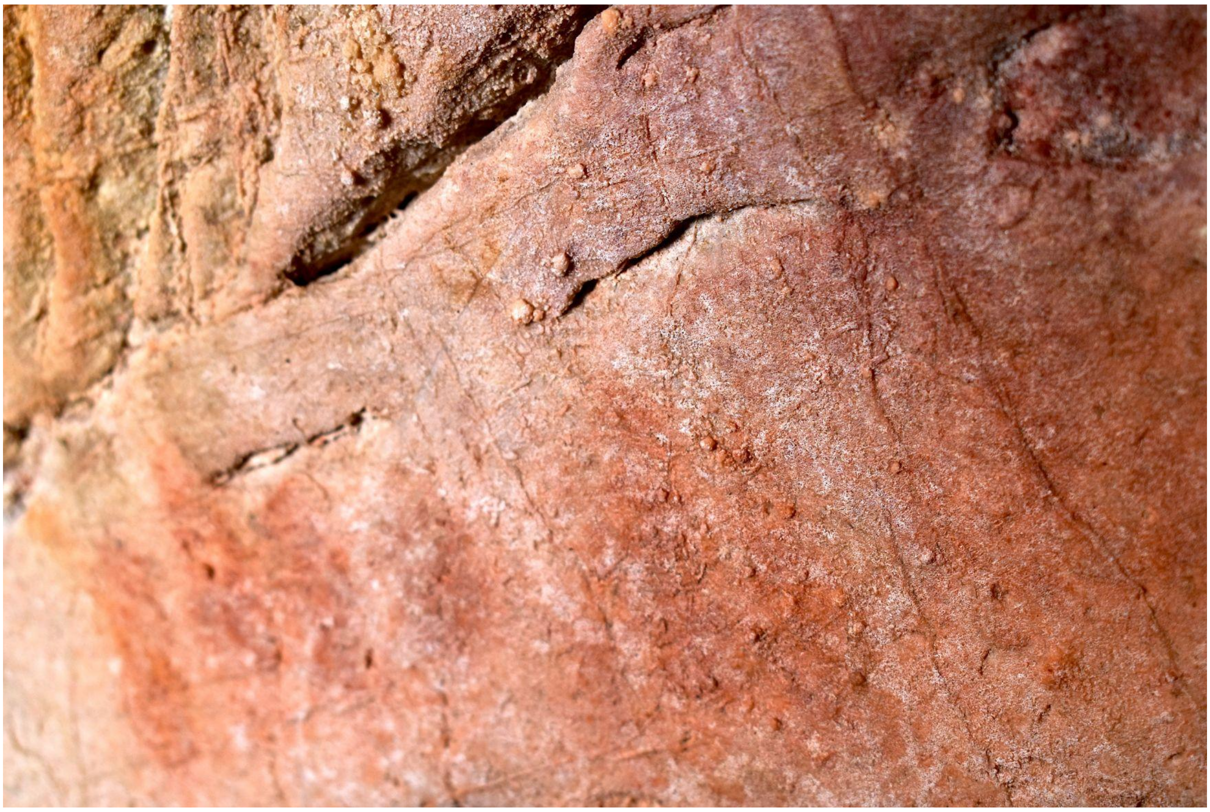

Figure S12. Macro-photo showing the purple-red hued pigment of a hand-stencil underlying the more vibrant, red pigment of a series of vertical lines, adjacent to depiction EC1.57.

This has also been noted by other previous studies that have assessed the chronology of this panel, and noted the purple-red colour of these depictions. A previous study using hyperspectral imaging also appeared to confirm that this colour relates to a genuine difference in pigments used for certain depictions (Ripoll-López et al. 2020; Ripoll et al. 2021). One of these depictions had been previously indirectly dated using U-Th dating (Pike et al., 2012), and had a minimum age that placed it as belonging to the early Aurignacian. While there are concerns over this dating technique (Sauvet et al., 2017), the purple-red hued depictions appear to consistently underlie all other depictions on the panel (Figure S13; Figure S14) and thus they are considered as representing the oldest phase of depictions on the *Ceiling of the Hands*.

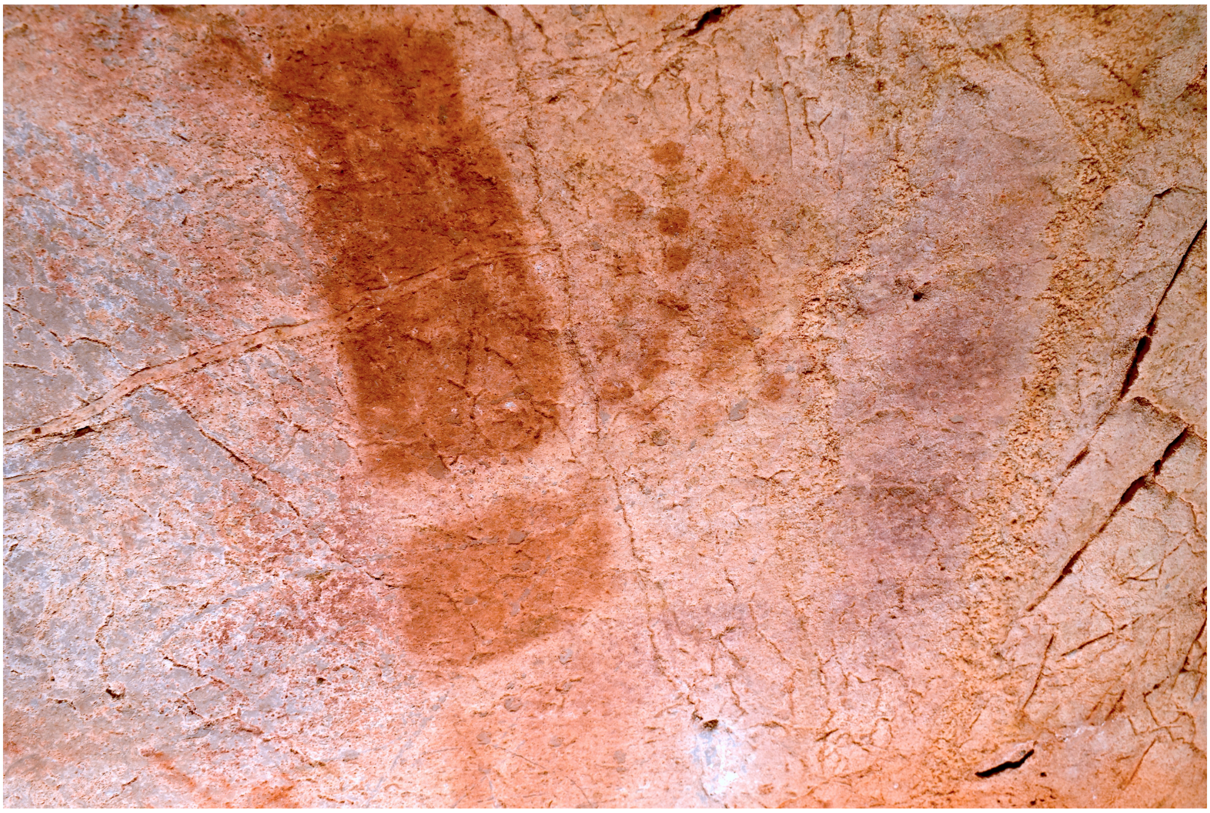

Figure S13. Purple-red hand-stencil underlying a series of red finger dots and a red non-figurative motif.

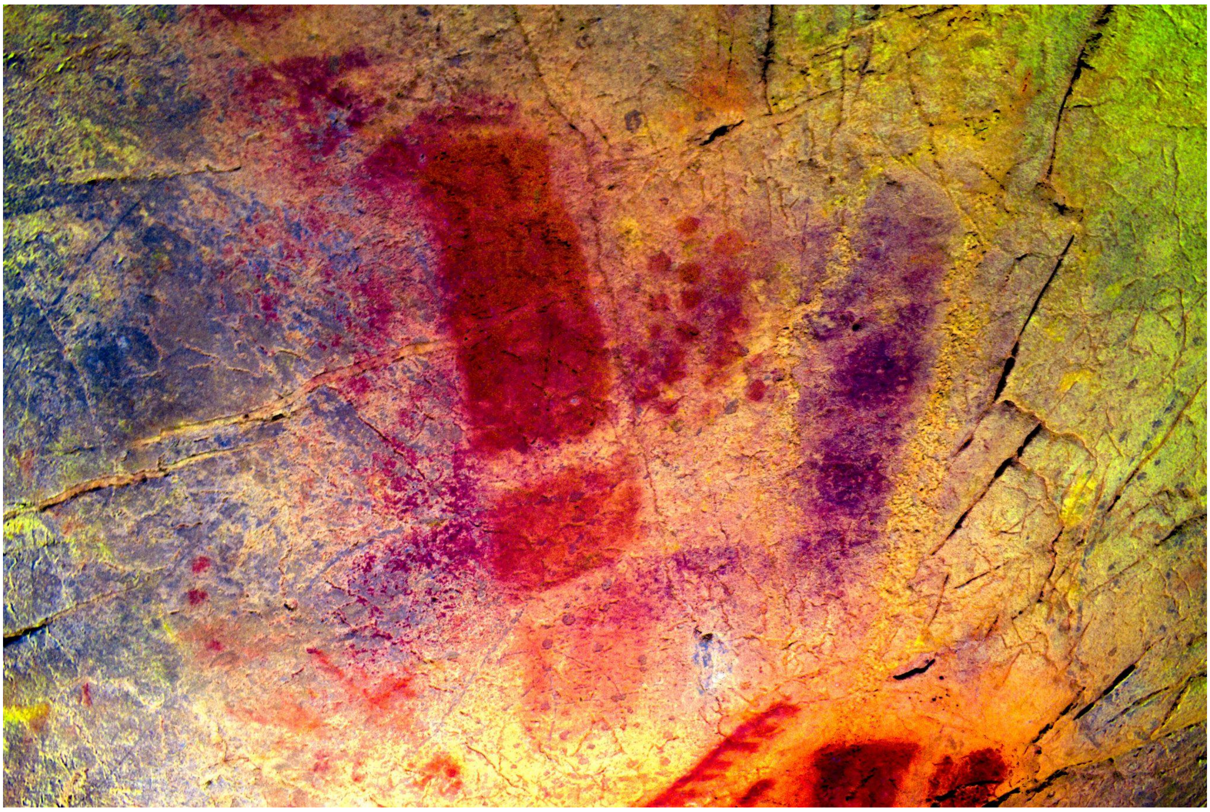

Figure S14. DStretch image of the same motifs as Figure S12, that more clearly visualises the underlying purple-red coloured hand-stencil.

2.2. Superimpositions: Phase 2

The second phase of depictions identified on the Ceiling of the Hands comprises a series of red depictions, hand-stencils and non-figurative motifs, that appears to overlie the purple-red hued depictions of Phase 1 (e.g., Figure S13; Figure S14) but underlie the yellow figurative depictions on the panel (Phase 3). The key characteristic that defined whether depictions were attributed to Phase 2 was whether they underlay yellow depictions, with any depictions that overlay the yellow depictions being grouped within Phase 4. For several depictions, particularly the non-figurative depictions towards the lower-left section of the panel, the grouping in Phase 2 was determined by their close spatial proximity and similarity in theme/form to other non-figurative depictions that did underlie yellow depictions (Figures S15-S17).

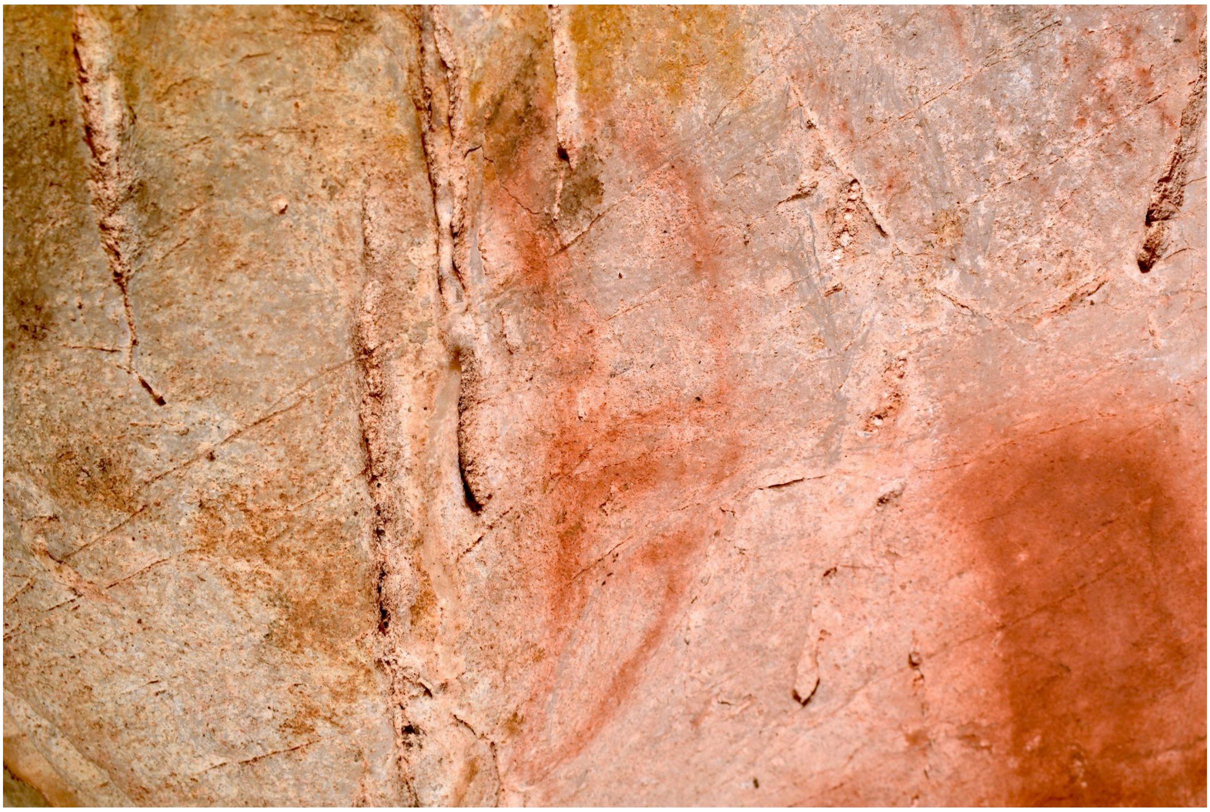

Figure S15. A yellow motif, towards the top of the image, appears to partially overlie the red sign.

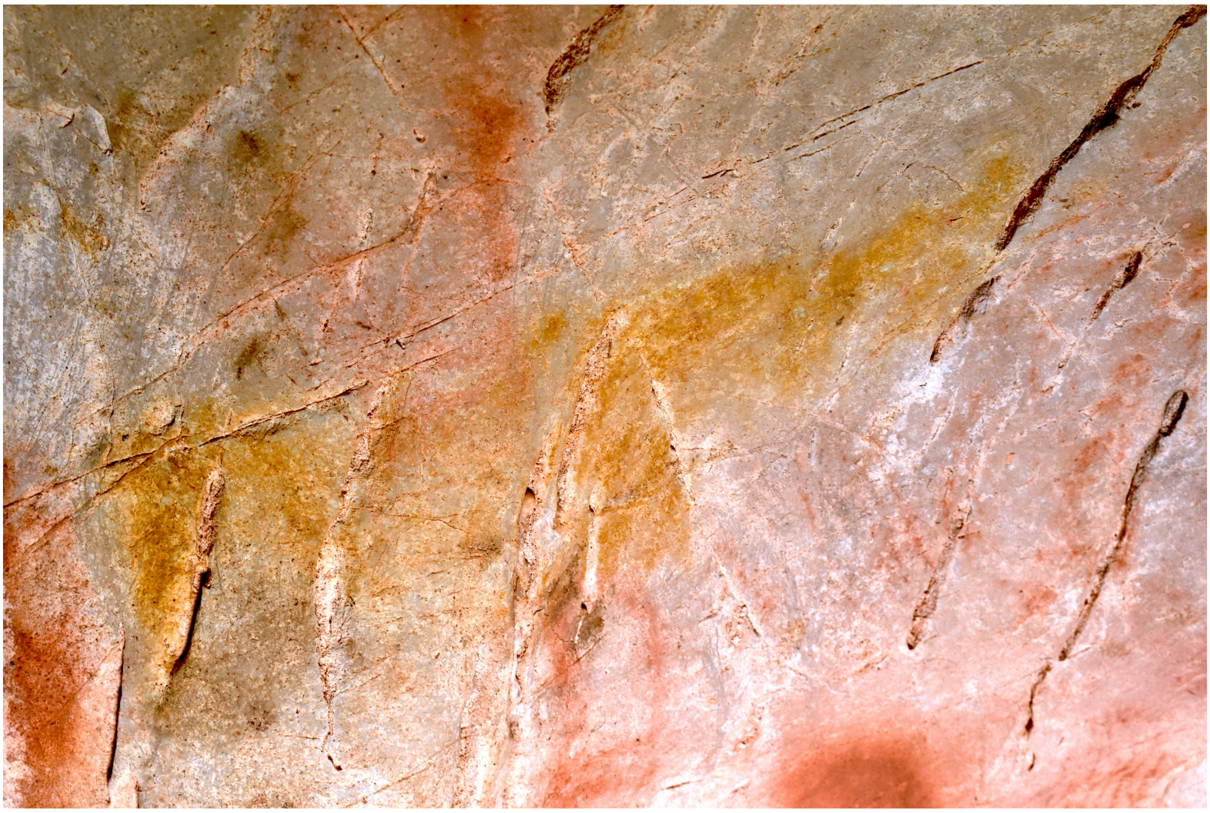

Figure S16. The same yellow motif from Figure S15 may additionally, albeit tentatively, overlie a vertical red line above.

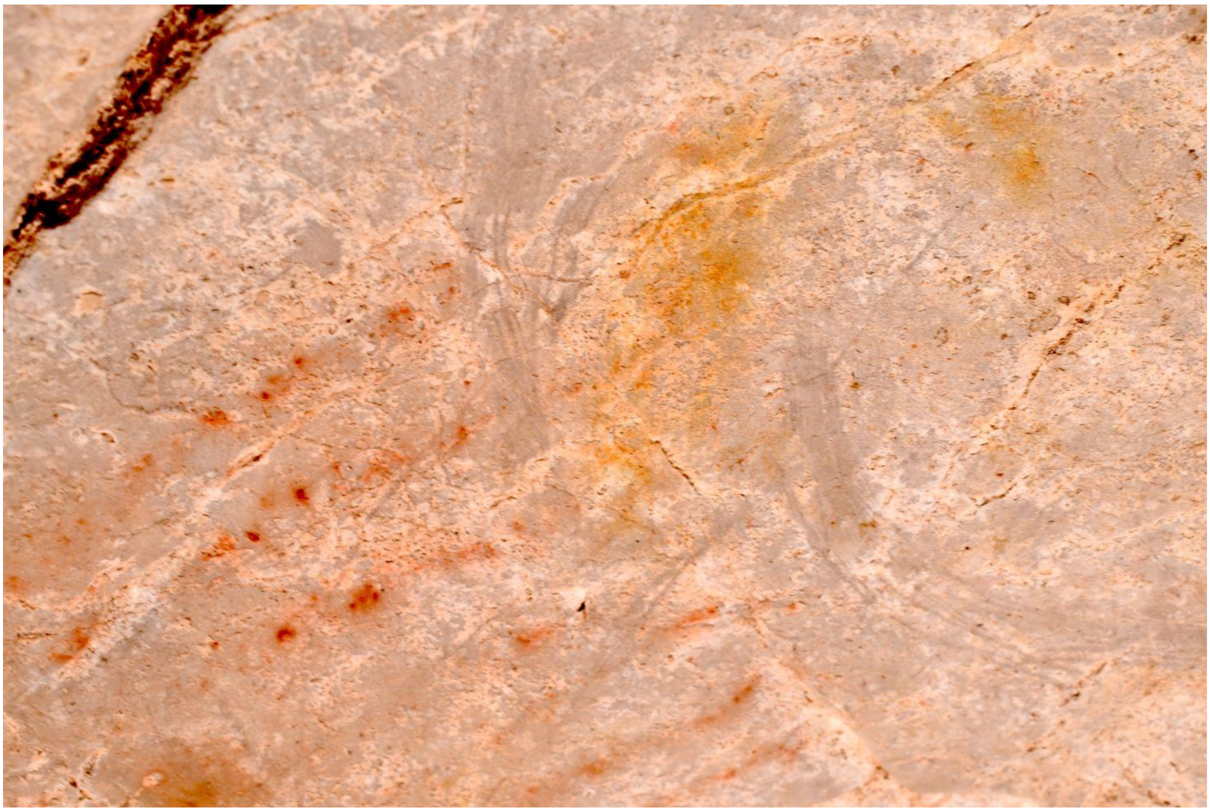

Figure S17. A macro-photo showing traces of yellow pigment that are superimposed over a series of thin horizontal red lines.

2.3. Superimpositions: Phase 3

The third phase of depictions was identified as being the series of yellow depictions that both under- and over-lie the red depictions on the panel (Phase 2 and 4 respectively). These yellow depictions consistently overlie the purple-red depictions characterised as Phase 1 in this panel, but have a mixed stratigraphic relationship to the other red motifs; both appearing to over- and under- lie these motifs. As such, these yellow depictions were considered to belong to one phase that separates a series of red depictions. As discussed previously and in the main manuscript, given this stratigraphic relationship between the yellow and red motifs, and the presence of a horse depiction close to the *Ceiling of the Hands* that has an outline produced with both red and yellow hued ochre, it is likely that these two pigments may have been used contemporaneously with Phases 2-4 perhaps representing one artistic phase of production. However, given the importance of exploring the relationship between superimpositions, it was necessary to separate these depictions into the three phases to allow for the different stratigraphic relationships between the red and yellow motifs to be fully characterised and explored.

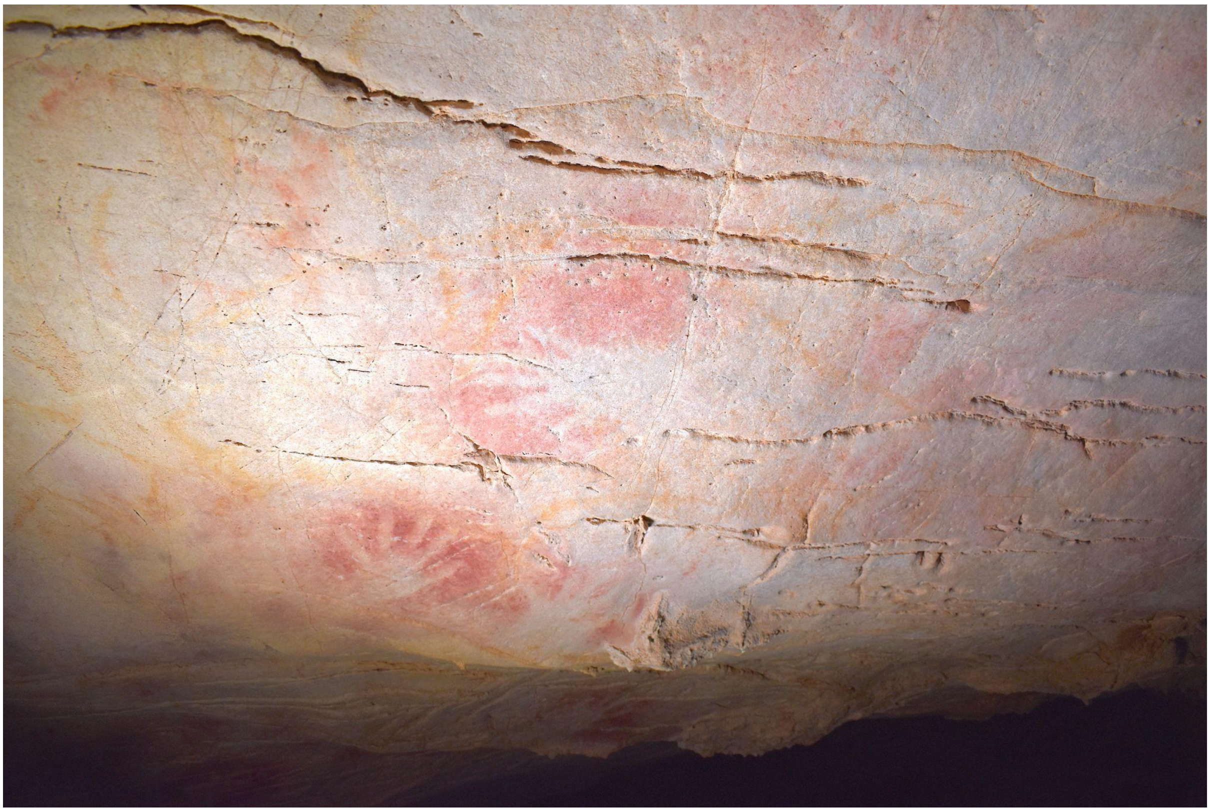

Figure S18. Depiction EC1.45 (yellow bison) that appears to have hand-stencils that both overlie and underlie the outline.

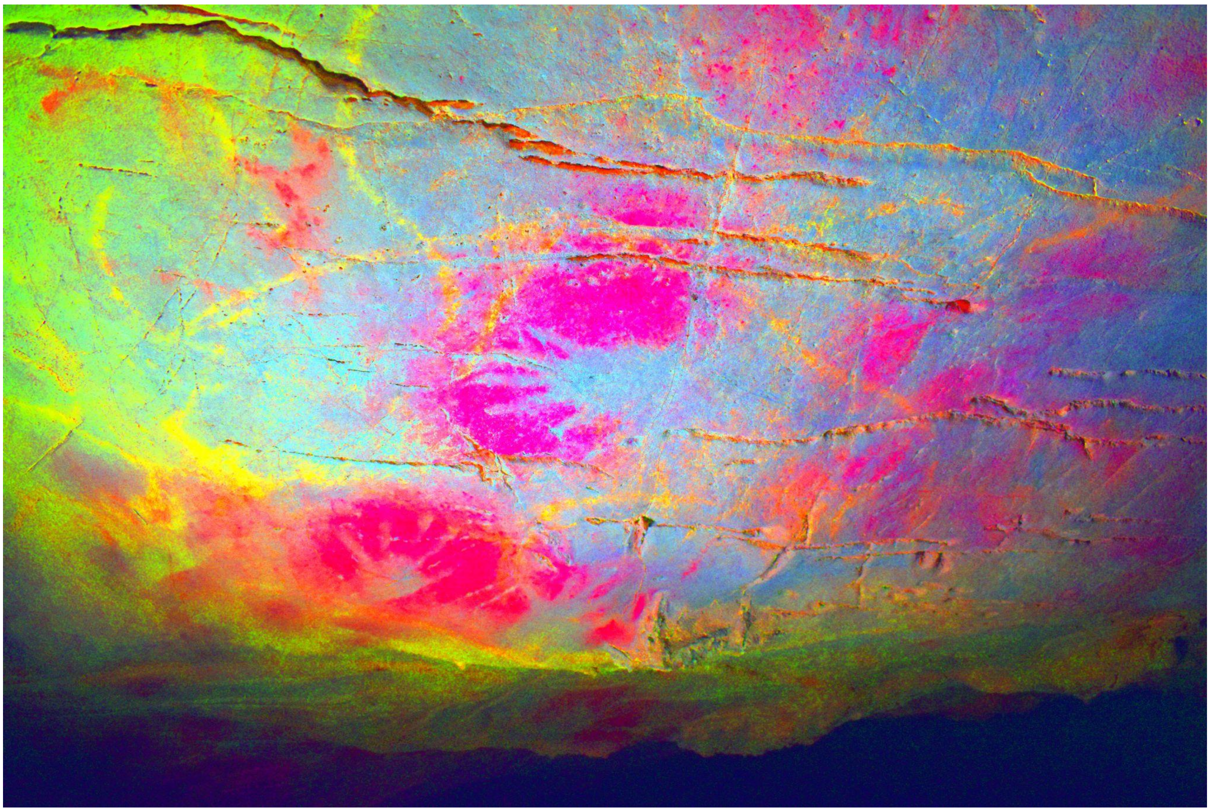

Figure S19. DStretch manipulated image of Figure S18 that more clearly visualises the different stratigraphic relationships of the hand-stencils associated with depiction EC1.45. Note the hand-stencils

in the centre and towards the rear of the depiction appear to underlie the outline, while the hand-stencil on the ventral line of the depiction appears to overlie the outline.

2.4. Superimpositions: Phase 4

As with Phase 2, this phase similarly consists of a series of red motifs, predominantly hand-stencils and finger marks. These are characterised as a separate phase due to the stratigraphic relationship to the yellow figurative depictions; these depictions *overlie* the yellow figurative depictions and thus must be considered as a separate phase than the red depictions of Phase 2 (Figure S11; Figure S12; Figures S20-S24). Given the similarity in theme, form, and technique of these depictions to Phase 2, it is proposed that the phases 2-4 may be closely temporally related, and may even be considered as one larger phase as proposed previously by (Ruiz-Redondo 2012). However, given that considering these three phases as one larger phase would subsume the superimposed relationships between depictions on the panel, which are the central focus of the study, these were considered as separate phases here.

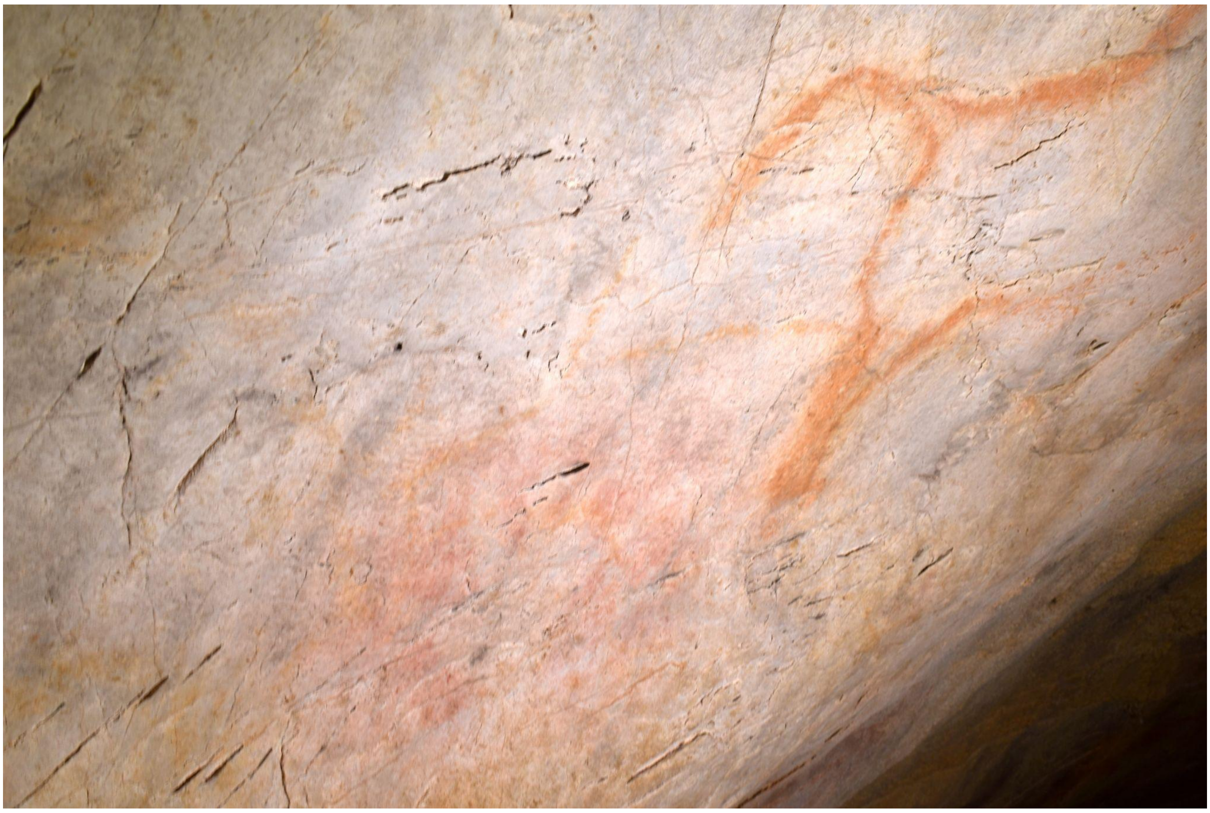

Figure S20. Faded figurative yellow and red hand-stencil motifs, whose stratigraphic order is better visualised through the application of DStretch (Figure S21).

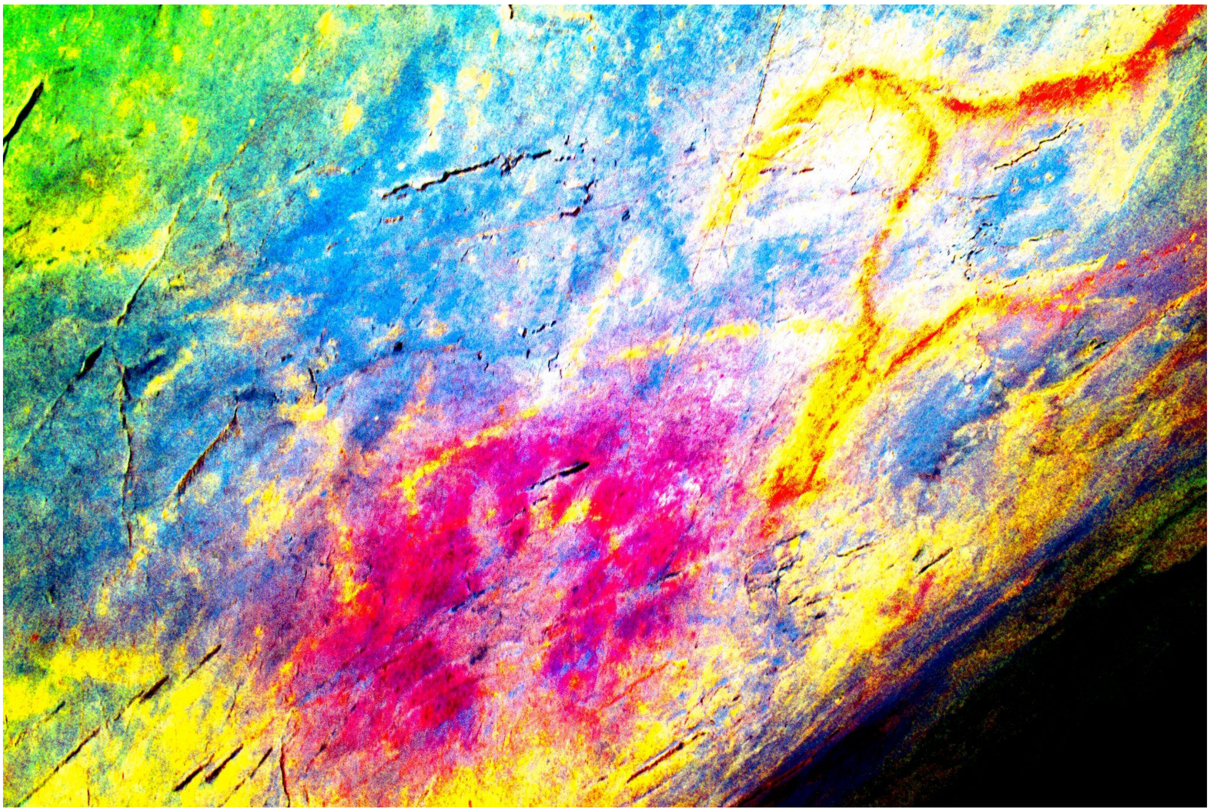

Figure S21. A DStretch manipulated image of the same motifs as in Figure S20, that more clearly visualises the stratigraphic relationship. Here, the hand-stencil appears to clearly overlie the yellow lines of an indeterminate figurative motif, where the yellow colouration of the lines can be viewed in the blank spaces of the fingers.

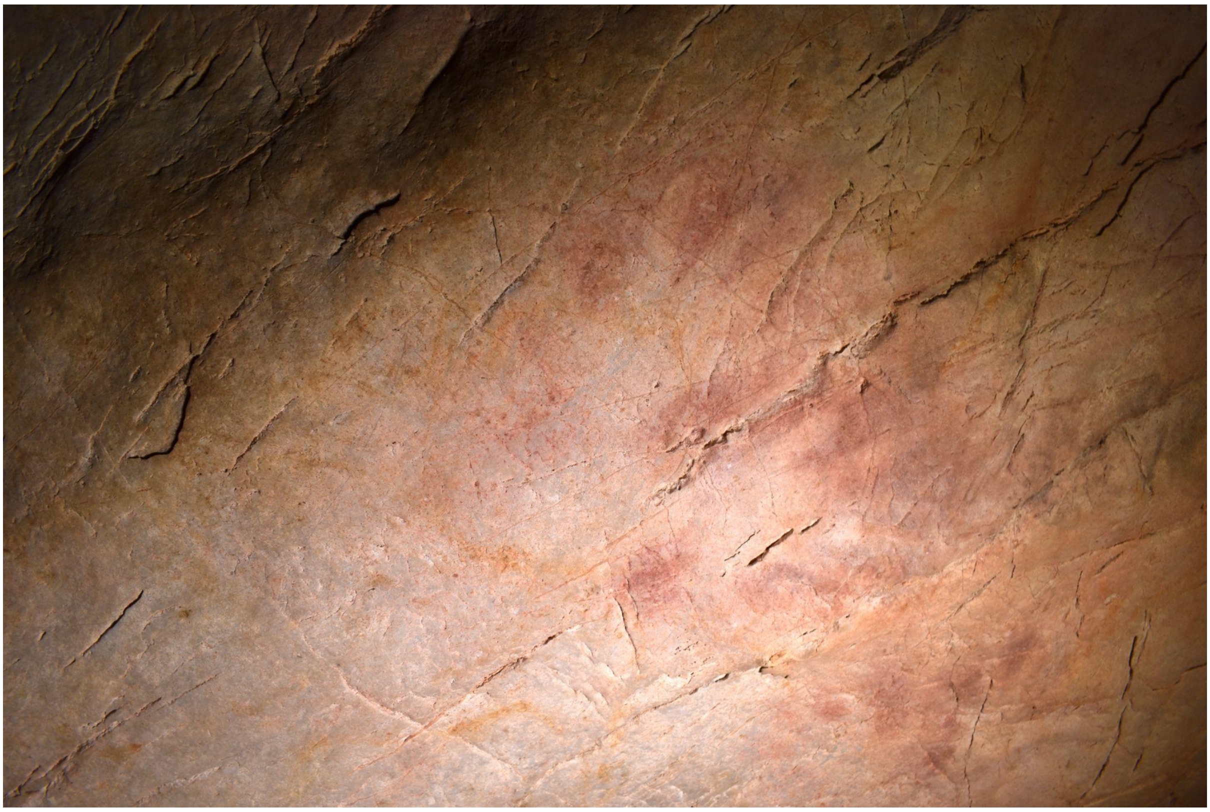

Figure S22. Two hand stencils (EC1.38 and EC1.39) that appear to be superimposed over a yellow motif.

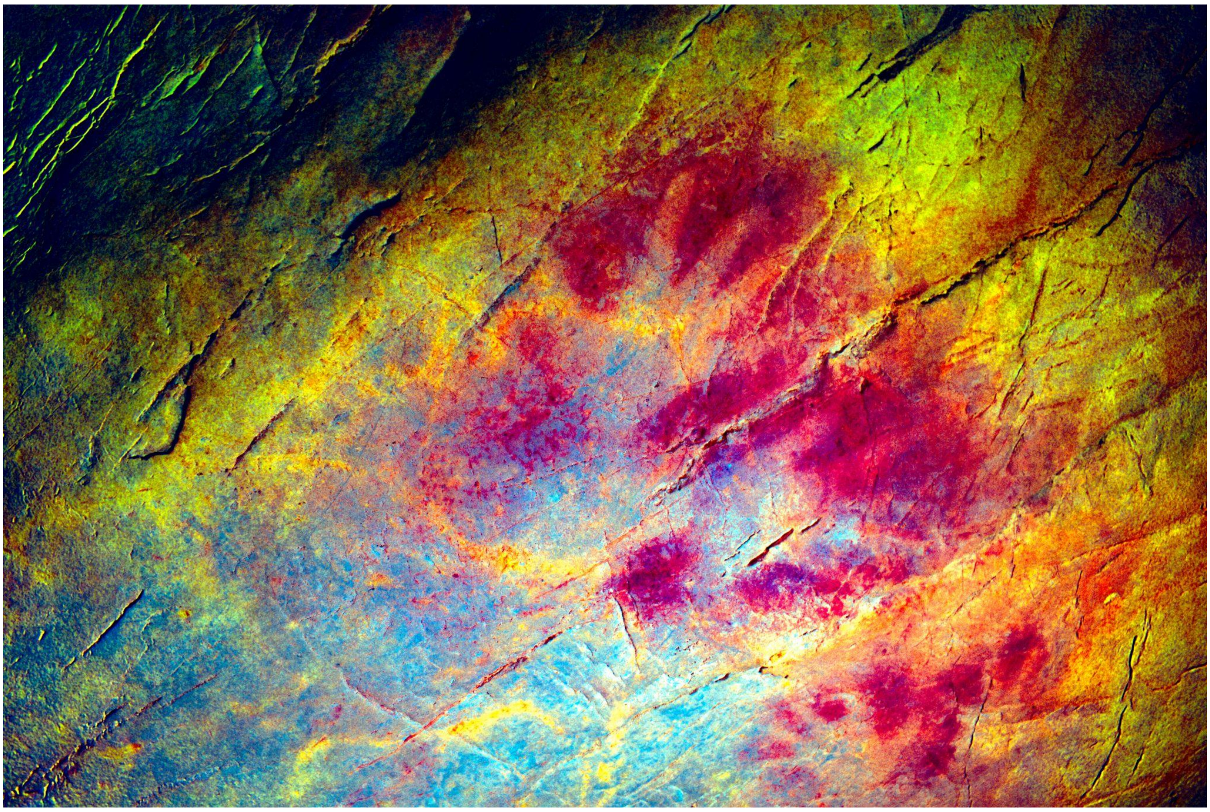

Figure S23. A DStretch manipulated image of the same depictions as in Figure S22, that more clearly visualises the stratigraphic relationship of the depictions.

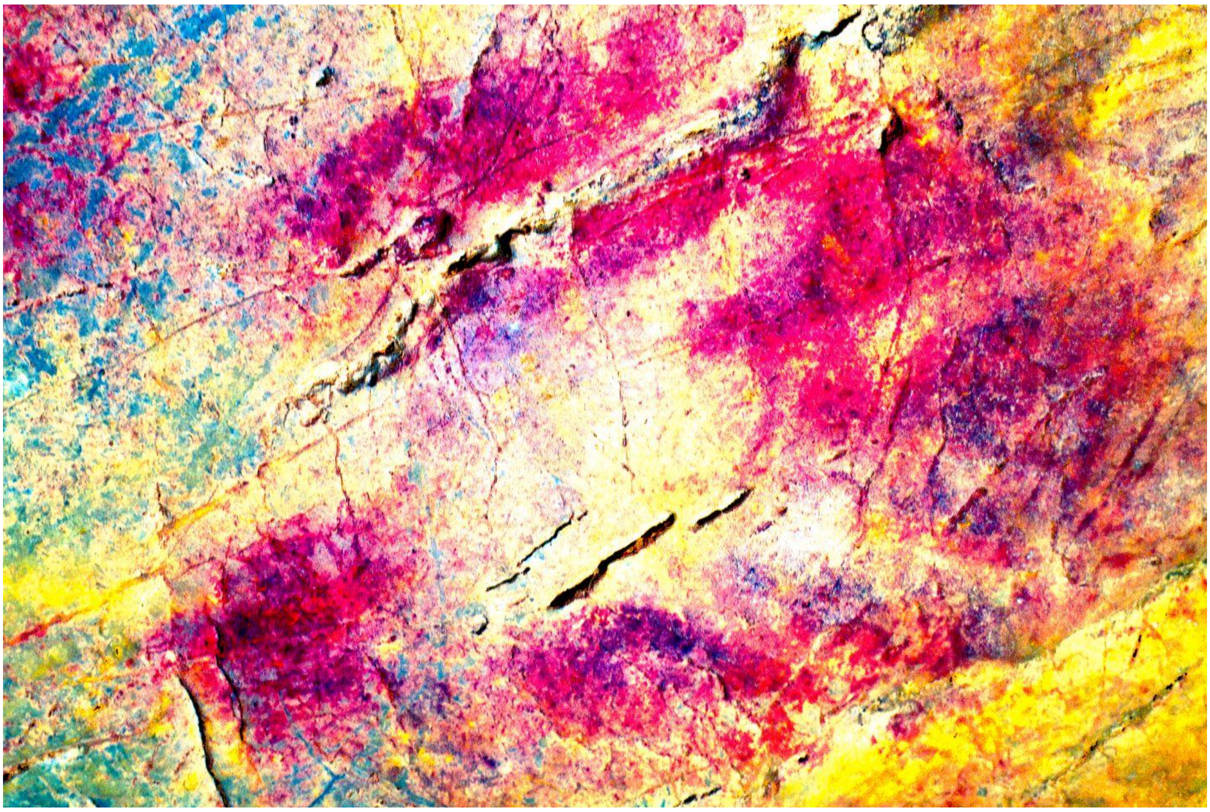

Figure S24. A macro-photo of depiction EC1.39 (see Figure S22 and S23) that further demonstrates the superimposition of this depiction over the yellow depiction.

2.5. Superimpositions: Phase 5

The final phase of graphic activity identified on the Ceiling of the Hands is a series of small engravings of both hinds (Figure S25; Figure S26 and non-figurative lines (Figure S27). These all consistently overlie the other depictions on the panel, and are predominantly constrained to the lower left portion of the panel. These thus represent the youngest phase of depictions on the panel. At least two of the engraved hinds appear to share features of the so-called "striated hinds" that have been generally attributed to the Lower Magdalenian (Ibero et al., 2024; Rivero et al., 2019: Figure S25; Figure S26). Tentatively, these depictions may therefore date to this period based on the presence of this characteristic trait.

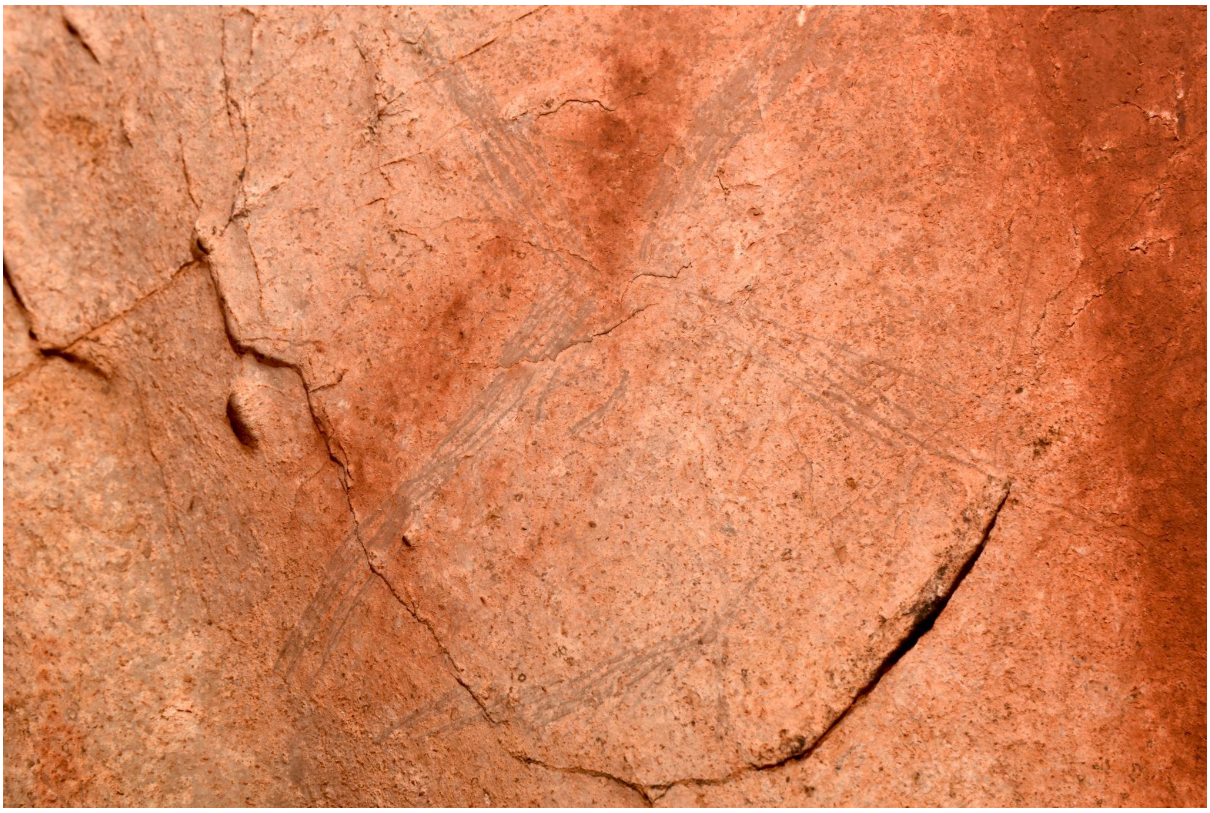

Figure S25. A hind head superimposed on a non-figurative red sign. The striated nature of the outline is similar to so-called striated hinds from the Lower Magdalenian.

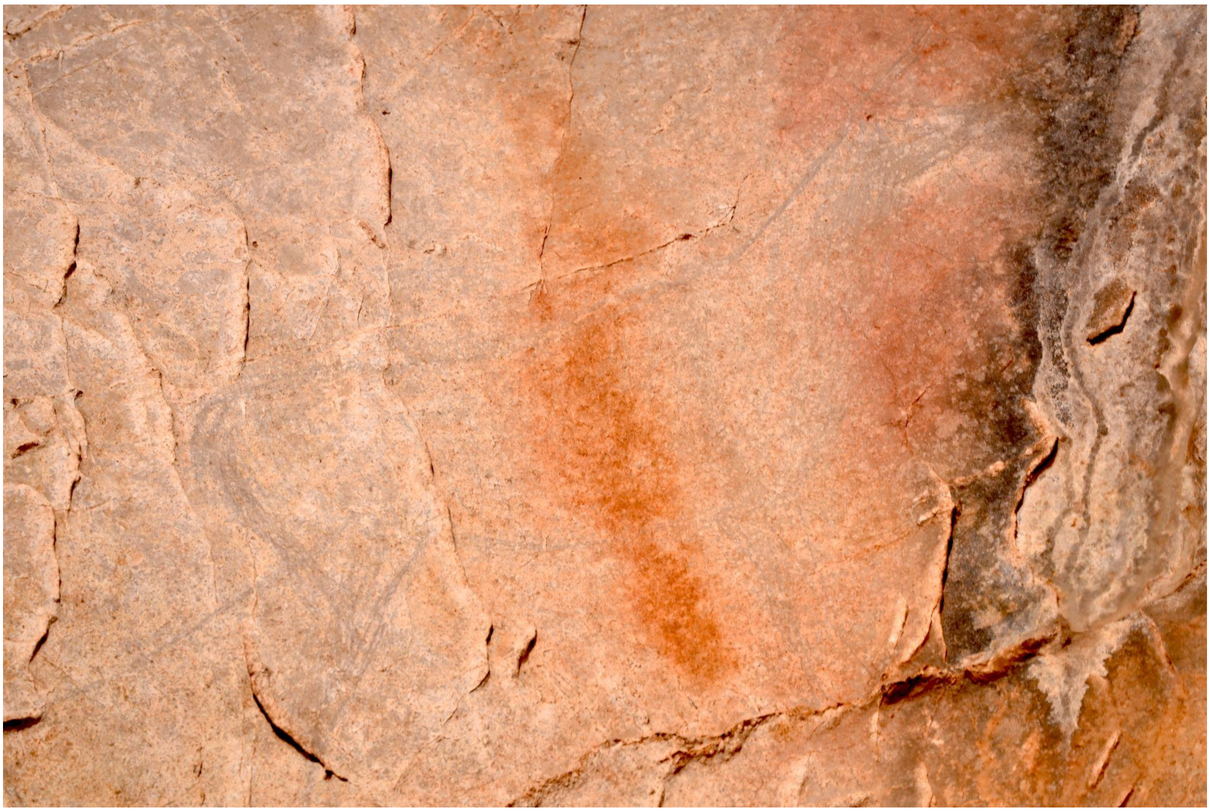

Figure S26. A complete engraved outline of a hind, superimposed over two red motifs. The head and rear of the depiction are infilled with striated engraved lines, consistent with the so-called "striated hinds" of the Lower Magdalenian.

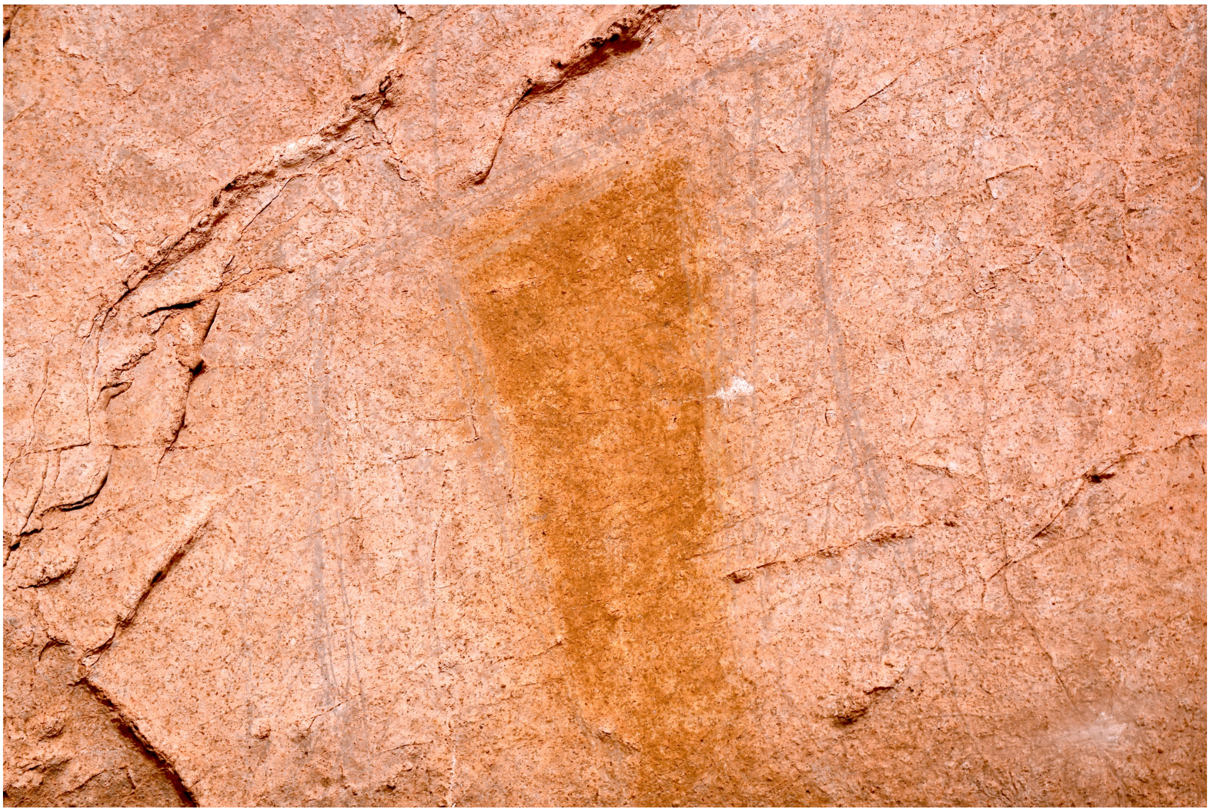

Figure S27. Non-figurative engraved lines that are superimposed and surround a yellow non-figurative motif.

Bibliography

Collado Giraldo, H. & Julio García Arranz, J. (2018) Cueva de El Castillo (Puente Viesgo). In: H. Collado (ed.), *Handpas. Manos del pasado: catálogo de representaciones de manos en el arte rupestre paleolítico de la Península Ibérica* (pp. 127-227). Merida: Junta de Extremadura.

Ibero, Á., García-Diez, M. & Ochoa, B. (2024) The Diachronic Construction of Paleolithic Cave Art: Striated Hind Heads of the Cantabrian Region. *Journal of Paleolithic Archeology 7:* 1-24. https://doi.org/10.1007/s41982-024-00191-1

Pike, A.W.G., Hoffmann, D.L., García-Diez, M., Pettitt, P.B., Alcolea, J., De Balbín, R., González-Sainz, C., De Las Heras, C., Lasheras, J.A., Montes, R. & Zilhão, J. (2012) U-Series Dating of Paleolithic Art in 11 Caves in Spain. *Science 336(6087):* 1409-1413

Rivero, O., Garate, D., Salazar, S. & Intxaurbe, I. (2019). The Cantabrian Lower Magdalenian striated hinds on scapulae: Towards a new definition of a graphic morphotype. *Quaternary International* 506: 69-79. https://doi.org/10.1016/j.quaint.2019.01.037

Ruiz Redondo, A. (2012) Una nueva revisión del Panel de las Manos de la cueva de El Castillo (Puente Viesgo, Cantabria). *Munibe Antropologia-Arkeologia 61*:17-27

Valladas, H., Tisnérat-Laborde, N., Cachier, H., Arnold, M., Bernaldo De Quirós, F., Cabrera-Valdés, V., Clottes, J., Courtin, J., Fortea-Pérez, J., Gonzáles Sainz, C. & Moure-Romanillo, A. (2001). Radiocarbon AMS dates for Paleolithic cave paintings. *Radiocarbon 43 (2B):* 977-986. https://doi.org/10.1017/S0033822200041643
